# Integrated Proteogenomic Characterization of Clear Cell Renal Cell Carcinoma

**DOI:** 10.1016/j.cell.2019.10.007

**Published:** 2019-10-31

**Authors:** David J. Clark, Saravana M. Dhanasekaran, Francesca Petralia, Jianbo Pan, Xiaoyu Song, Yingwei Hu, Felipe da Veiga Leprevost, Boris Reva, Tung-Shing M. Lih, Hui-Yin Chang, Weiping Ma, Chen Huang, Christopher J. Ricketts, Lijun Chen, Azra Krek, Yize Li, Dmitry Rykunov, Qing Kay Li, Lin S. Chen, Umut Ozbek, Suhas Vasaikar, Yige Wu, Seungyeul Yoo, Shrabanti Chowdhury, Matthew A. Wyczalkowski, Jiayi Ji, Michael Schnaubelt, Andy Kong, Sunantha Sethuraman, Dmitry M. Avtonomov, Minghui Ao, Antonio Colaprico, Song Cao, Kyung-Cho Cho, Selim Kalayci, Shiyong Ma, Wenke Liu, Kelly Ruggles, Anna Calinawan, Zeynep H. Gümüş, Daniel Geiszler, Emily Kawaler, Guo Ci Teo, Bo Wen, Yuping Zhang, Sarah Keegan, Kai Li, Feng Chen, Nathan Edwards, Phillip M. Pierorazio, Xi Steven Chen, Christian P. Pavlovich, A. Ari Hakimi, Gabriel Brominski, James J. Hsieh, Andrzej Antczak, Tatiana Omelchenko, Jan Lubinski, Maciej Wiznerowicz, W. Marston Linehan, Christopher R. Kinsinger, Mathangi Thiagarajan, Emily S. Boja, Mehdi Mesri, Tara Hiltke, Ana I. Robles, Henry Rodriguez, Jiang Qian, David Fenyö, Bing Zhang, Li Ding, Eric Schadt, Arul M. Chinnaiyan, Zhen Zhang, Gilbert S. Omenn, Marcin Cieslik, Daniel W. Chan, Alexey I. Nesvizhskii, Pei Wang, Hui Zhang, Clinical Proteomic Tumor Analysis Consortium

**Affiliations:** 1 Department of Pathology, Johns Hopkins University, Baltimore, MD 21231, USA; 2 Department of Pathology, University of Michigan, Ann Arbor, MI 48109, USA; 3 Department of Genetics and Genomic Sciences and Icahn Institute for Data Science and Genomic Technology, Icahn School of Medicine at Mount Sinai, New York, NY 10029, USA; 4 Department of Population Health Science and Policy, Icahn School of Medicine at Mount Sinai, New York, NY 10029, USA; 5 Tisch Cancer Institute, Icahn School of Medicine at Mount Sinai, New York, NY 10029, USA; 6 Lester and Sue Smith Breast Center, Baylor College of Medicine, Houston, TX 77030, USA; 7 Urologic Oncology Branch, Center for Cancer Research, National Cancer Institute, National Institutes of Health, Bethesda, MD 20892, USA; 8 Washington University School of Medicine, St. Louis, MO 63110, USA; 9 Department of Public Health Sciences, University of Chicago, Chicago, IL 60637, USA; 10 Department of Translational Molecular Pathology, MD Anderson Cancer Center, Houston, TX 77030, USA; 11 Department of Public Health Sciences, University of Miami Miller School of Medicine, Miami, FL 33136, USA; 12 Institute for Systems Genetics and Department of Biochemistry and Molecular Pharmacology, New York University School of Medicine, New York, NY 10016, USA; 13 Department of Medicine, New York University School of Medicine, New York, NY 10016, USA; 14 Department of Computational Medicine and Bioinformatics, University of Michigan, Ann Arbor, MI 48109, USA; 15 Departments of Medicine and Cell Biology and Physiology, Washington University School of Medicine, St. Louis, MO 63110, USA; 16 Department of Biochemistry and Cellular Biology, Georgetown University, Washington, DC 20007, USA; 17 Brady Urological Institute and Department of Urology, Johns Hopkins University, Baltimore, MD 21231, USA; 18 Sylvester Comprehensive Cancer Center, University of Miami Miller School of Medicine, Miami, FL 33136, USA; 19 Department of Surgery, Urology Service, Memorial Sloan Kettering Cancer Center, New York, NY 10065, USA; 20 Department of Urology, Poznań University of Medical Sciences, Szwajcarska 3, Poznań 61-285, Poland; 21 Department of Medicine, Washington University School of Medicine, St. Louis, MO 63110, USA; 22 Sloan Kettering Institute, Memorial Sloan Kettering Cancer Center, New York, NY 10065, USA; 23 Department of Genetics and Pathology, Pomeranian Medical University, Szczecin 71-252, Poland; 24 International Institute for Molecular Oncology, Poznań 60-203, Poland; 25 Poznań University of Medical Sciences, Poznan 60-701, Poland; 26 Office of Cancer Clinical Proteomics Research, National Cancer Institute, Bethesda, MD 20892, USA; 27 Frederick National Laboratory for Cancer Research, Frederick, MD 21702, USA; 28 Department of Ophthalmology, Johns Hopkins University, Baltimore, MD 21231, USA; 29 Department of Molecular and Human Genetics, Baylor College of Medicine, Houston, TX 77030, USA; 30 Sema4, Stamford, CT 06902, USA; 31 Department of Internal Medicine, Human Genetics, and School of Public Health, University of Michigan, Ann Arbor, MI 48109, USA; 32 These authors contributed equally; 33 These authors contributed equally; 34 Lead Contact

## Abstract

To elucidate the deregulated functional modules that drive clear cell renal cell carcinoma (ccRCC), we performed comprehensive genomic, epigenomic, transcriptomic, proteomic, and phosphoproteomic characterization of treatment-naive ccRCC and paired normal adjacent tissue samples. Genomic analyses identified a distinct molecular subgroup associated with genomic instability. Integration of proteogenomic measurements uniquely identified protein dysregulation of cellular mechanisms impacted by genomic alterations, including oxidative phosphorylation-related metabolism, protein translation processes, and phospho-signaling modules. To assess the degree of immune infiltration in individual tumors, we identified microenvironment cell signatures that delineated four immune-based ccRCC subtypes characterized by distinct cellular pathways. This study reports a large-scale proteogenomic analysis of ccRCC to discern the functional impact of genomic alterations and provides evidence for rational treatment selection stemming from ccRCC pathobiology.

## In Brief

Comprehensive proteogenomic characterization in 103 treatment-naive clear cell renal cell carcinoma patient samples highlights tumor-specific alterations at the proteomic level that are unrevealed by transcriptomic profiling and proposes a revised subtyping scheme based on integrated omics analysis.

## Graphical Abstract

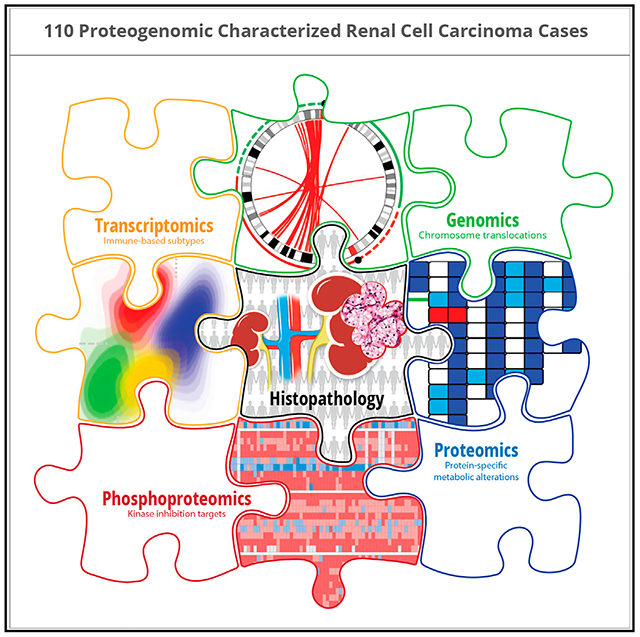

## INTRODUCTION

Renal cell carcinoma (RCC) is among the top ten most commonly diagnosed cancers worldwide (Siegel et al., 2018). Clear cell renal cell carcinoma (ccRCC) is the predominant histology of renal cell carcinoma, representing 75% of all cases and the majority of cancer-associated deaths (Hsieh et al., 2017). To understand the underlying molecular alterations that drive ccRCC oncogenesis, The Cancer Genome Atlas (TCGA) has performed extensive genomic, epigenomic, and transcriptomic profiling, identifying discriminating features of ccRCC that include loss of various tumor suppressor genes (Creighton et al., 2013; Ricketts et al., 2018). Aberrant dysregulation of the *VHL* gene is a nearly universal founding event. Subsequent genomic alterations involving *PBRM1, SETD2, KDM5C*, or *BAP1* are required for disease progression and are associated with aggressive phenotypes (Hakimi et al., 2013; Kapur et al., 2013). These studies have highlighted the value of molecular characterization, in addition to histological assessment, to stratify ccRCC patients, while identifying genomic features unique to ccRCC tumorigenesis (Chen et al., 2016a).

Historically, ccRCC has been considered resistant to conventional chemotherapy and radiotherapy, with surgical resection as the primary treatment for localized tumors (Blanco et al., 2011; Diamond et al., 2015). Despite several Food and Drug Administration (FDA)-approved agents that target cellular pathways prioritized by genomic analyses, response of ccRCC patients to these treatments has been limited (Hsieh et al., 2018a). These results illustrate the complexity of tumorigenesis processes and suggest that genomic, epigenomic, and transcriptomic profiling alone may be insufficient to interrogate this cancer type fully for identifying effective curative treatments. In this study, the Clinical Proteomics Tumor Analysis Consortium (CPTAC) has performed a comprehensive proteogenomic characterization of treatment-naive tumors and paired normal adjacent tissues (NATs) to elucidate the impact of genomic alterations driving phenotypic perturbations and to delineate the mechanisms of ccRCC pathobiology for prospective exploration of personalized, precision-based clinical care.

## RESULTS

### Proteogenomic Analyses of Tumor and NAT Specimens

In this study, 110 treatment-naive RCC and 84 paired-matched NAT samples were analyzed using a proteogenomic approach wherein each tissue was homogenized via cryopulverization and aliquoted to facilitate genomic, transcriptomic, and proteomic analyses on the same tissue sample (STAR Methods). Patient characteristics, including age, gender, race, and tumor grade and stage, were recorded for all cases and summarized in Table S1. Proteomics and phosphoproteomics analyses identified a total of 11,355 proteins and 42,889 phosphopeptides, respectively, of which 7,150 proteins and 20,976 phosphopeptides were quantified across all samples (STAR Methods). To enable multi-omics data integration and proteogenomic analysis, whole genome sequencing (WGS), whole exome sequencing (WES), and total RNA sequencing (RNA-seq) were performed for all 110 tumor samples, while 107 tumor samples had quality DNA methylation profiling data (Figure S1A; Table S1). NAT samples with mRNA of sufficient quality were subjected to total RNA-seq (n = 75). One NAT sample that displayed discordant proteogenomic profiles was found to contain significant histological evidence of tumor tissue and was excluded from downstream analyses (Figure S1A; Table S1). In addition to the initial pathological diagnosis, we leveraged the molecular information available for RCCs by TCGA and others to verify further the histological classification of tumor samples (STAR Methods; Creighton et al., 2013; Davis et al., 2014; Mehra et al., 2016, 2018; Linehan et al., 2016). Sample-wise assessment of genomic profiles identified seven tumors with molecular aberrations atypical for ccRCC, such as lacking the characteristic bi-allelic loss of tumor suppressor genes on 3p (Figures S1B–S1D; Table S2). While these seven non-ccRCC samples and their corresponding NATs (n = 3) were excluded from most subsequent analyses, the non-ccRCC samples served as useful controls to highlight ccRCC-specific features. Overall, data from 103 ccRCC and 80 NAT tissue samples (with RNA-seq profiles available for 72 samples) were examined for comprehensive proteogenomic characterization (Table S1).

### Genomic Landscape of the CPTAC ccRCC Cohort

**Figure 1.**
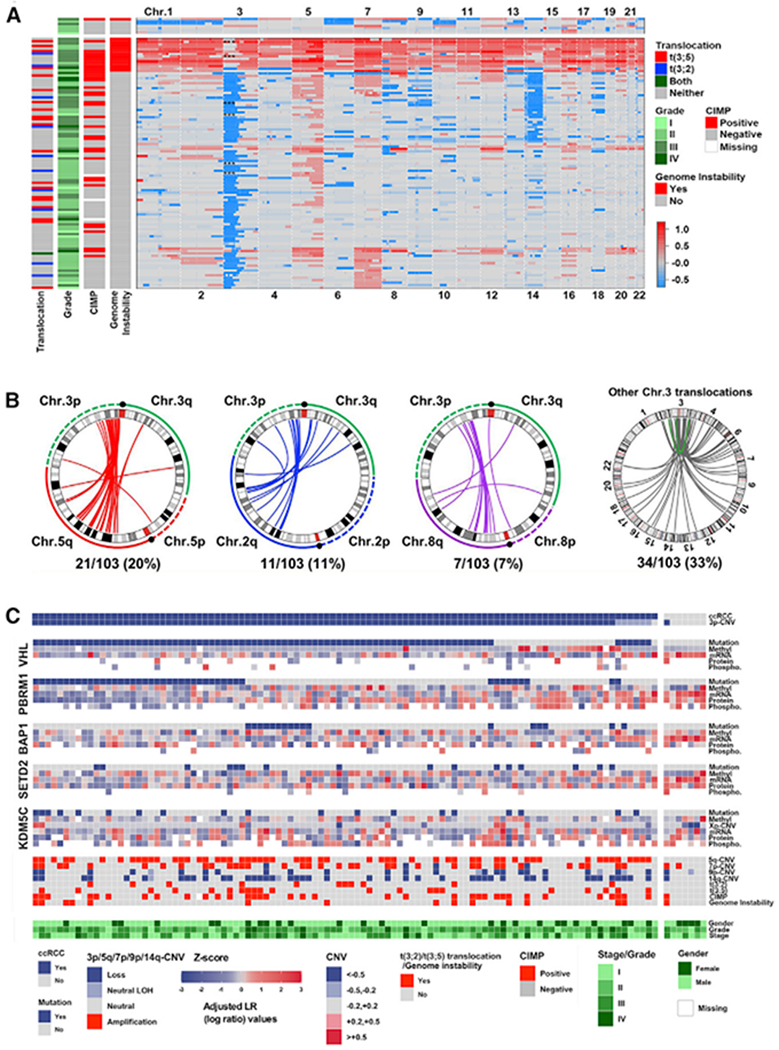
Genomic Alterations and their Associations with mRNA, Protein, and Phosphoprotein Abundances (A) Profiling of absolute copy number estimates observed in the CPTAC cohort. Genomically defined non-ccRCC tumors are above ccRCC tumors; translocation event, grade, CpG island methylator phenotype (CIMP) status, genome instability, and CNV loss/gain are indicated by color coding. ccRCC tumors with evidence of 3p loss of heterozygosity (LOH) are indicated by three asterisks (***). (B) Circos plots of translocation events involving chromosomes 3 and either chromosomes 5 (red), 2 (blue), 8 (purple) or all other chromosomes (gray), including chromosomal inversion within chromosome 3 (green). Percentage of involved tumors with re-arrangement for each chromosome is annotated below each plot. (C) Heatmap of multi-omic data for the five key tumor suppressor genes (*VHL, PBRM1, BAP1, SETD2*, and *KDM5C*) (n = 103). Tumor samples were ordered by 3p CNV alteration (loss to neutral). Non-ccRCC tumors are separated (right). CNV event, *Z* score, CNV loss/gain, translocation status, CpG island methylator phenotype (CIMP) status, genome instability, grade, and gender are indicated by color coding (bottom). See also Figure S1 and Tables S1 and S2.

Our study represents a large WGS analysis of ccRCC, revealing arm-level loss of chromosome 3p as the most frequent event (93%), followed by chromosome 5q gain (54%), chromosome 14q loss (42%), chromosome 7 gain (34%), and chromosome 9 loss (21%) (Figure 1A; Table S2). Strikingly, we observed fourteen tumors in our cohort displayed extensive CNVs across all chromosomes, indicating a high degree of genomic instability. A molecular subset with these characteristics was not identified in the initial TCGA ccRCC study, possibly due to the limited number of tumors examined via WGS (Creighton et al., 2013). Moreover, a recent pan-cancer analysis of three common RCC subtypes and studies investigating ccRCC tumor ploidy via an intra-tumor heterogeneity approach showed a significant association between high genome aneuploidy and poor prognosis (Chen et al., 2016a; Turajlic et al., 2018a, 2018b), which is consistent with the enrichment of genomically instable high grade tumors identified in our study (Figure 1A).

A previous report identified chromosome translocation as a mechanism of concurrent 3p loss and 5q gain in ccRCC (Mitchell et al., 2018). Utilizing the WGS data in our study, we investigated the frequency and types of chromosomal rearrangements present in our cohort. We observed that 61% of ccRCC cases displayed one or more translocation events, predominantly involving the chromosome 3p locus and chromosomes 5 (20%), 2 (11%), and 8 (7%) among others (Figure 1B; Table S2). The novel chromosome t(3:2) rearrangement, largely observed in cases with concurrent 3p loss and 2q gain, was the second most common translocation event and was nearly mutually exclusive with t(3:5) (Figure 1B). We also detected chromosomal inversion within chromosome 3 (n = 2) that resulted in concurrent 3q gain and 3p loss (Figure 1B). A previous study suggested chromothripsis as a likely mechanism of concurrent 3p loss and 5q gain in ccRCC (Mitchell et al., 2018); we noted a similar genomic feature of oscillating copy number patterns near the breakpoint (data not shown). In addition, 3p loss of heterozygosity (LOH) was observed in six tumors (Table S2). In total, 102 of 103 ccRCC tumors in the CPTAC cohort showed evidence of 3p dysregulation.

The profile of somatic mutations in the CPTAC ccRCC cohort was reflective of that previously described by TCGA (Creighton et al., 2013). Dysregulation of *VHL* was the most frequent alteration and was observed in 85% of tumors. *PBRM1, BAP1, KDM5C*, and *SETD2* followed with mutation rates of 43%, 17%, 18%, and 16%, respectively (Figure 1C). We further examined the impact of mutation and methylation of these genes on their respective levels of mRNA and protein. Overall, inactivating genomic events resulted in reduced expression of mRNA and protein (Figure 1C), indicating loss-of-function and supporting the classification of these genes as ccRCC tumor suppressors (Frew and Moch, 2015; Liao et al., 2015). Unique to SETD2 was the relationship of gene inactivation due to t(3:2), with a higher frequency of mutations and reduced protein expression relative to other translocation events involving chromosome 3p (Figures S1E and S1F). Investigation into the rate of mutation co-occurrence revealed that *VHL* mutations were associated with the presence of other mutations (*PBRM1, BAP1, SETD2*, and *KDM5C*), while *PBRM1* mutations were associated with mutation of *SETD2* and *KDM5C* but not *BAP1* (Figure S1G). These results support a model of branched evolution in ccRCC and the largely mutual exclusivity of somatic mutations involving *PBRM1* and *BAP1* (Gerlinger et al., 2014; Turajlic et al., 2018a).

Increased methylation of promoter CpG islands contributes to an oncogenic phenotype (Baylin and Jones, 2011). Querying DNA methylation status of CpG positions with a previously reported CpG island methylator phenotype (CIMP) marker panel specific for ccRCC (Arai et al., 2012), we classified tumors in our cohort into CIMP^−^ (negative) and CIMP^+^ (positive) categories (Figure S1H). We identified 36 tumors (34%) with CIMP^+^ status, which was associated with higher grade (p < 9.0 e–05) and stage (p < 0.001), and higher frequency of genomic instability (p < 0.004) (Figure 1A; Table S2).

### Integrated Proteogenomic Analyses of Genomic Alterations

**Figure 2.**
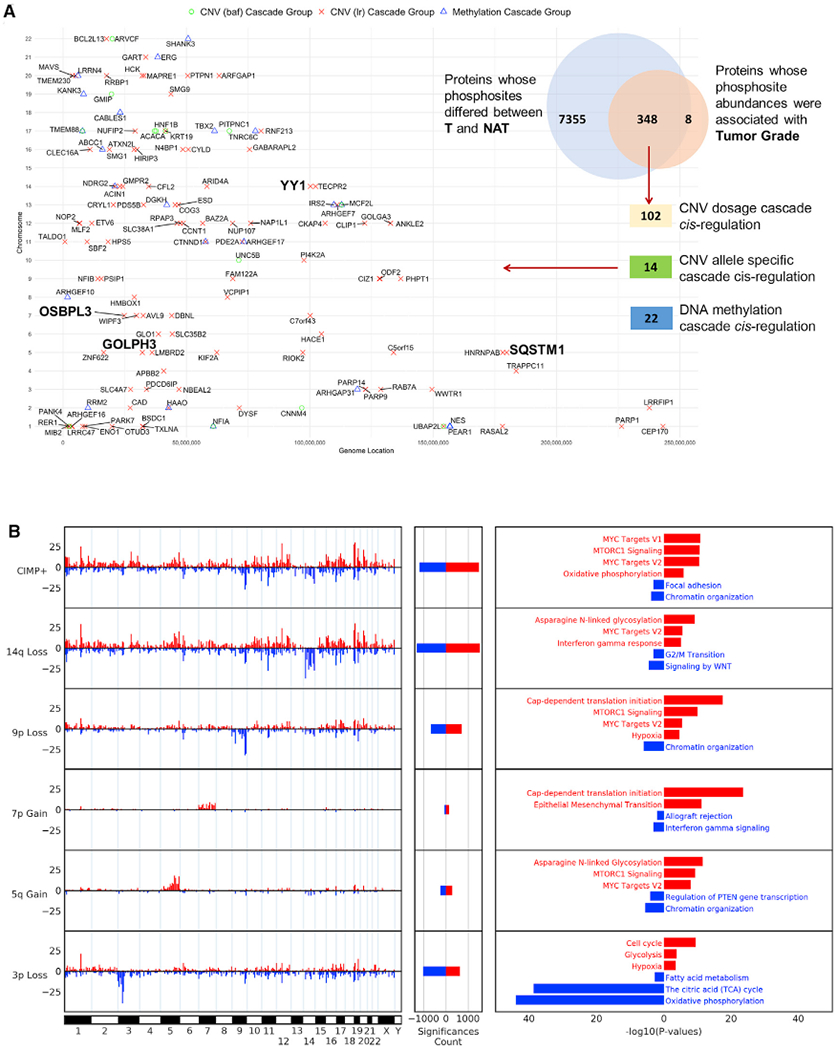
Impact of Copy Number Variation (CNV) on Protein Abundance (A) DNA variations (CNV baf, B-allele frequencies; CNV Ir, adjusted log coverage ratios; DNA methylation) with cascading *cis*-association (associations with all types of mRNA, global protein, and phosphopeptide abundances), overlapped with phosphopeptides significantly differentiated by clinical features (tumor versus NAT and tumor grade). Genes in bold are associated with CNV events involving chromosome 5 or 7 gain and 14 loss. (B) The *cis* and *trans* associations of chromosome arms (3p, 5q, 7p, 9p, and 14q) and CpG island methylator phenotype (CIMP). Significant (adjusted p < 0.1) positive (red) and negative (blue) associations for individual chromosomes (left), summed associations (middle), and corresponding enriched upregulated (red) and downregulated (blue) pathways (adjusted p < 0.05) are annotated (right). See also Figure S2 and Table S3.

Genomic alterations can impact mRNA and protein abundance at the same locus (*cis*-effects), as well as other loci (*trans*-effects). Through integration of mRNA, protein, and phosphopeptide levels, we identified genomic alterations preserved through transcriptional, translational, and post-translational levels (*cis*-effect cascades), prioritizing gene targets associated with tumor grade and differential abundance between tumors and NATs (Figures 2A, S2A, and S2B; Table S3). Examples of identified CNV *cis*-cascades included *SQSTM1* (5q35.3), *OSBPL3* (7p15.3), and *GOLPH3* (5q13.3), previously associated with PI3K-mTOR signaling (Creighton et al., 2013; Lehto and Olkkonen, 2003; Scott et al., 2009). We also identified a *cis*-cascade involving the multifunctional transcription factor *YY1* (14q32.2), which is a stabilizer of HIF-1α, co-repressor of HIF-2α activity, and inhibitor of *MYC* function (Austen et al., 1998; Petrella and Brinckerhoff, 2009; Wu et al., 2013). The latter observation suggests a mechanism whereby *YY1* loss links the HIF-2α signaling with MYC expression in ccRCC (Gordan et al., 2008). Investigation into methylation events proximal to *VHL* identified three genes, *VGLL4* (3p25.2), *PLCL2* (3p24.3), and *IQSEC1* (3p25.2) with associated *cis*-regulated effects (Figure S2C). Methylation of *PLCL2* and *IQSEC1* has been noted previously (Dmitriev et al., 2014); however, methylation of *VGLL4* has not been reported. Its functional role inhibiting YAP-induced cell proliferation (Zhang et al., 2014b) may represent another tumor suppressor gene in ccRCC. Interestingly, unique to *IQSEC1*, we found that gene methylation was associated with reduced protein and phosphorylation levels, but not mRNA. This distinctive feature of methylation affecting protein but not mRNA expression was also observed in several other genes, including *BCL9L* (11q23.3) and *AHDC1* (1p35.3) (Figure S2C; Table S3), and may indicate a post-translational regulatory mechanism.

Next, we focused on identifying cellular processes correlated with *cis*- and *trans*-effects driven by major CNV or mutation events in ccRCC and identified multiple pathways that were disparate or commonly dysregulated among distinct genomic alterations (Figures 2B and S2D; Table S3). Loss of chromosome 3p was associated with upregulation of hypoxic signaling, cell-cycle regulation and glycolysis, downregulation of oxidative phosphorylation (OXPHOS), fatty acid metabolism, and the TCA cycle. Increased expression of HIF-1α and HIF-2α is a mechanism for upregulating hypoxic signaling upon *VHL* loss (Guo et al., 2011; Krieg et al., 2000). In cancer cells, HIF1 signaling alters mitochondrial activity and drives a metabolic shift from OXPHOS to glycolysis (Papandreou et al., 2006). Investigation of *trans*-effects involving chromosome 3p genes revealed that *VHL* mutations resulted in dysregulation of similar pathways as 3p loss including downregulation of metabolic pathways and upregulation of G1/S cell-cycle transition and interferon-α response. *PBRM1* mutations drove downregulation of OXPHOS and upregulation of G2/M cell-cycle transition, mitogen-activated protein kinase (MAPK) signaling, and focal adhesion pathways. *BAP1* mutations were associated with upregulation of protein translation pathways and interferon-γ signaling, with the latter feature also associated with *SETD2* mutations (Figure S2D; Table S3). *KD5MC* mutations shared a similar trans-effect profile as 3p locus/VHL loss, including downregulation of select metabolic pathways and increased cell-cycle regulation. 5q gain resulted in increased mTORC1 and MYC signaling, while gain of 7p drove increased protein translation and epithelial mesenchymal transition (EMT) (Figure 2B). 9p loss, which includes the tumor suppressor *CDKN2A*, was associated with upregulation of translation initiation, mTOR and MYC signaling, consistent with loss of *CDKN2A* and *MYC* activation previously reported in ccRCC progression (Bailey et al., 2017). Loss of 14q, involving the potential tumor suppressors *NDRG2* (14q11.2) and *HIF1A* (14q23.2) (Lusis et al., 2005; Shen et al., 2011), displayed decreased WNT signaling expression and upregulation of MYC signaling, *N*-linked glycosylation, and interferon-γ response. We detected a high percentage of CIMP^+^ tumors with 14q loss (75%) (Figure 1C). CIMP^+^ tumors shared a similar *trans*-effect profile, including increased MYC signaling and protein translation, with a unique signature associated with increased OXPHOS and decreased focal adhesion (Figure 2B; Table S3).

### Correlation of Transcriptome and Proteome Expression

**Figure 3.**
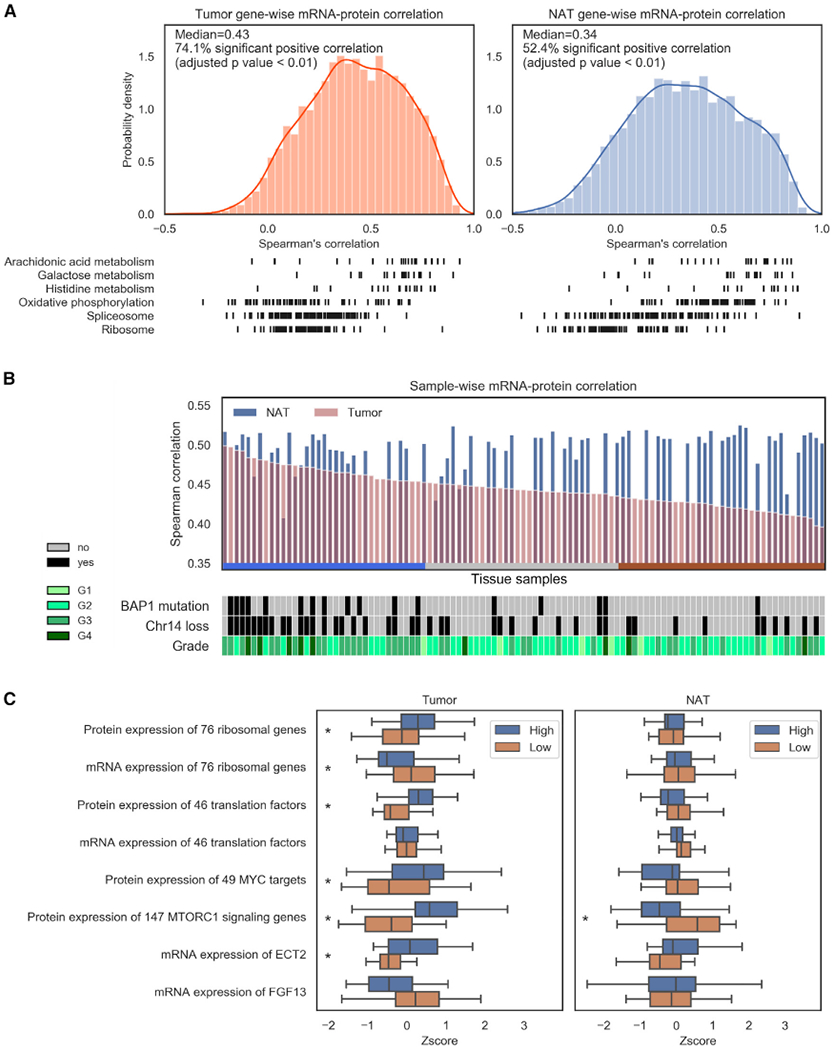
Correlations between Transcriptomic and Proteomic Abundance (A) Gene-wise correlations of mRNA and protein expression in tumors (left) and NATs (right). Annotated cellular pathways and corresponding Spearman gene-wise correlation (bottom). (B) Sample-wise correlation of tumors ranked from high to low with corresponding NAT sample-wise correlation (top). Tumors were evenly distributed into three groups: high (blue), middle (gray), and low (gold). BAP1 mutation, chromosome 14 loss status, and tumor grade are annotated (bottom). (C) Boxplots of ribosome and translation factor gene expression and Pol I-associated regulation in tumor samples (left) and corresponding NATs (right) (*p < 0.05). Figure S3 and Tables S1 and S4.

To understand the regulatory relationship between transcriptomic and proteomic processes, we calculated gene-wise (inter-sample) and sample-wise (intra-sample) correlation of 7,027 mRNA-protein pairs for the 103 ccRCC tumors and 72 NATs. NATs displayed a median gene-wise correlation value of 0.34, while tumors displayed a higher median value of 0.43, which is similar to previous studies investigating colorectal and high grade serous ovarian cancers (Figure 3A; Table S4; Zhang et al., 2014a, 2016). For ccRCC tumors and NATs, 74% and 52% of mRNA-protein pairs had significant positive Spearman correlations, respectively (Figure 3A; Benjamini-Hochberg adjusted p < 0.01), with OXPHOS, spliceosome, and ribosome pathways poorly correlated in tumors. In NATs, the housekeeping activities of the spliceosome and ribosome displayed a distinct correlation pattern, representing a differentially regulated axis to maintain cellular homeostasis (Komili and Silver, 2008).

Examination of sample-wise mRNA-protein correlation indicated a lower median sample-wise correlation for tumor samples (0.44) than for NAT samples (0.52), which had lower variance (Figures 3B and S3A; Benjamini-Hochberg adjusted p < 0.001). Adjusting for tumor purity (STAR Methods), we detected a trend of higher sample-wise correlation in tumors associated with clinical features such as higher grade (p = 0.006), chromosome 14 loss (p = 0.0006), and *BAP1* mutations (p = 0.00004) (Figure 3B; Table S4). Linking high sample correlation to increased protein translation, we identified a concordant increase of ribosome and translation factor protein expression (Figure 3C). Delineating a mechanism of increased ribosome biogenesis via Pol I transcription regulation (Pelletier et al., 2018), we detected a corresponding increase of protein expression for MYC-targets and mTORC1 signaling genes (p < 0.05), increased mRNA levels of the Pol I transcription activator, ECT2 (p < 0.05), and decreased mRNA levels for the Pol I transcription inhibitor, FGF13, in tumors with high sample-wise correlation compared to those with low sample-wise correlation (Figure 3C). In contrast, corresponding NATs did not display differences between the tumor-based classifications of high/low sample-wise correlation or express differential levels of ribosome and translation-related proteins (Figures 3C, S3B, and S3C). Interestingly, there was a concerted shift of discordant ribosome protein-mRNA levels in tumors (Figure 3C). However, the dysregulated mechanisms that contribute to the uncoupling of ribosomal mRNA and protein expression in tumors are less clear, suggesting the importance of protein evidence when evaluating ribosome biogenesis as a therapeutic target for intervention in ccRCC (Devlin et al., 2016).

### Proteogenomic Alterations of ccRCC Compared to NATs

**Figure 4.**
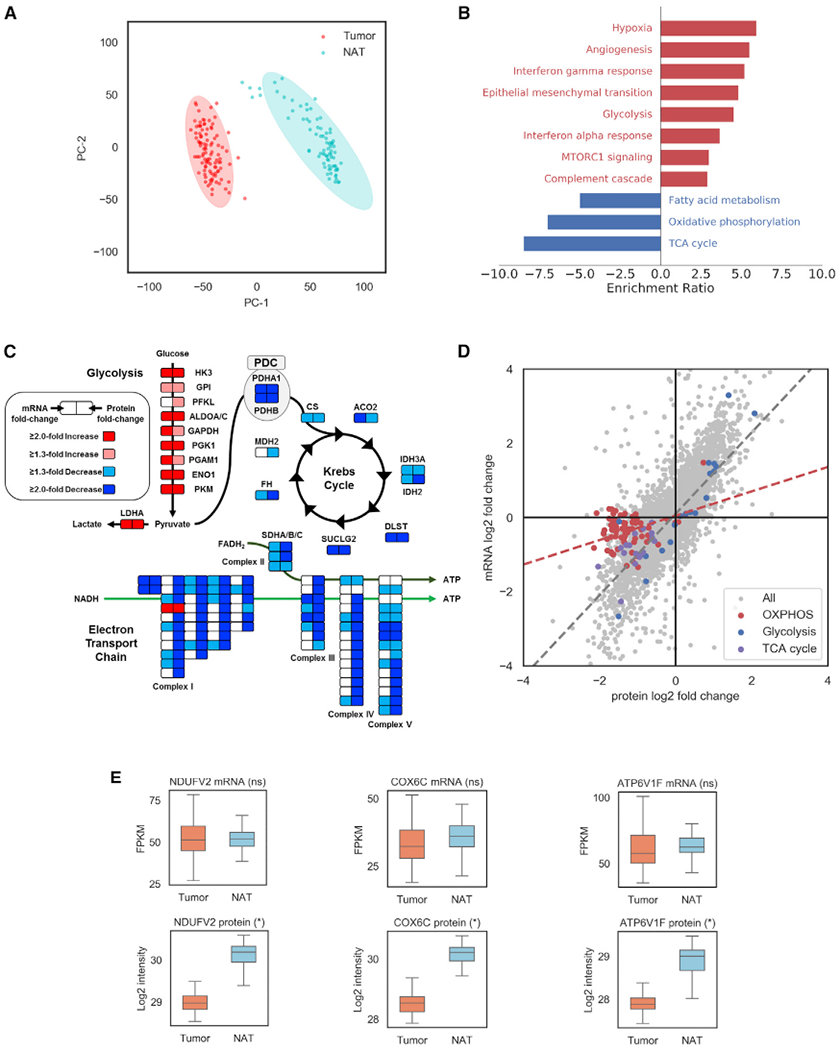
Differential Expression of Transcriptomic and Proteomic Profiles between ccRCC Tumors and NAT Protein Expression (A) PCA visualization of protein expression in ccRCC tumors and NATs. (B) Analysis of significantly differentially regulated pathways (adjusted p < 0.05) between ccRCC tumors and NATs. (C) Schema of metabolic pathways (glycolysis and electron transport chain [OXPHOS]) with select differential gene expression of mRNA and protein levels between ccRCC tumors and NATs. (D) Scatterplots depicting expression of mRNA (x axis) and protein (y axis). Linear regression of all mRNA-protein pairs (gray dotted line) and OXPHOS mRNA-gene pairs (red dotted line) are shown. Metabolism-related genes are indicated. (E) Boxplot of representative OXPHOS genes from complex I (*NDUFV2*), IV (*COX6C*), and V (*ATP6V1F*) displaying discordant mRNA-protein expression (n.s., not significant, *adjusted p < 0.05). See also Figure S4 and Tables S1, S4, and S5.

Visualization of the abundance of identified proteins by principal component analysis (PCA) and hierarchical clustering showed clear discrimination between ccRCC (n = 103) and NAT samples (n = 80) (Figures 4A and S4A). In total, 820 proteins showed significant differential expression in a group comparison of tissue samples (log2 fold-change >1; Benjamini-Hochberg adjusted p < 0.05), with 565 proteins downregulated and 255 upregulated in ccRCC tumors relative to NATs (Figure S4B; Table S5). Enrichment analysis revealed immune response, EMT, and multiple signaling pathways (hypoxia, glycolysis, and mTOR) to be upregulated in tumors, and TCA cycle, fatty acid metabolism, and OXPHOS to be downregulated (adjusted p < 0.05; Figure 4B). Select cellular pathways were maintained even when accounting for tissue heterogeneity in both tumors and NAT samples (Table S5). ccRCC tumors are characterized by particular genomic alterations that have resulted in their classification as a metabolic disease (Wettersten et al., 2017), which prompted us to identify and annotate differentially abundant mRNA transcripts and proteins involved in cellular metabolism (Figure 4C; Table S4). Proteins in the glycolysis pathway and their cognate mRNAs were upregulated in this analysis, whereas proteins associated with OXPHOS were downregulated. A non-linear correlation has been previously observed between metabolic mRNA levels and corresponding glycolytic and OXPHOS metabolites in ccRCC (Hakimi et al., 2016). Analyzing the differential abundances of mRNA and protein levels between tumors and NAT revealed a prominent uncoupling of OXPHOS mRNA and protein expression that was disparate from other cellular pathways (Figures 4C–4E and S4C), which reflects the regulation of select OXPHOS components at the translational level (Richman et al., 2016). Together, these results show that the functional consequences of the Warburg effect are not fully captured at the transcriptional level, which could impact the clinical use of transcription-based metabolic signatures for prognosis of ccRCC (Creighton et al., 2013).

### Phosphoproteomic Analysis of Kinase and Substrate Regulatory Pathways

**Figure 5.**
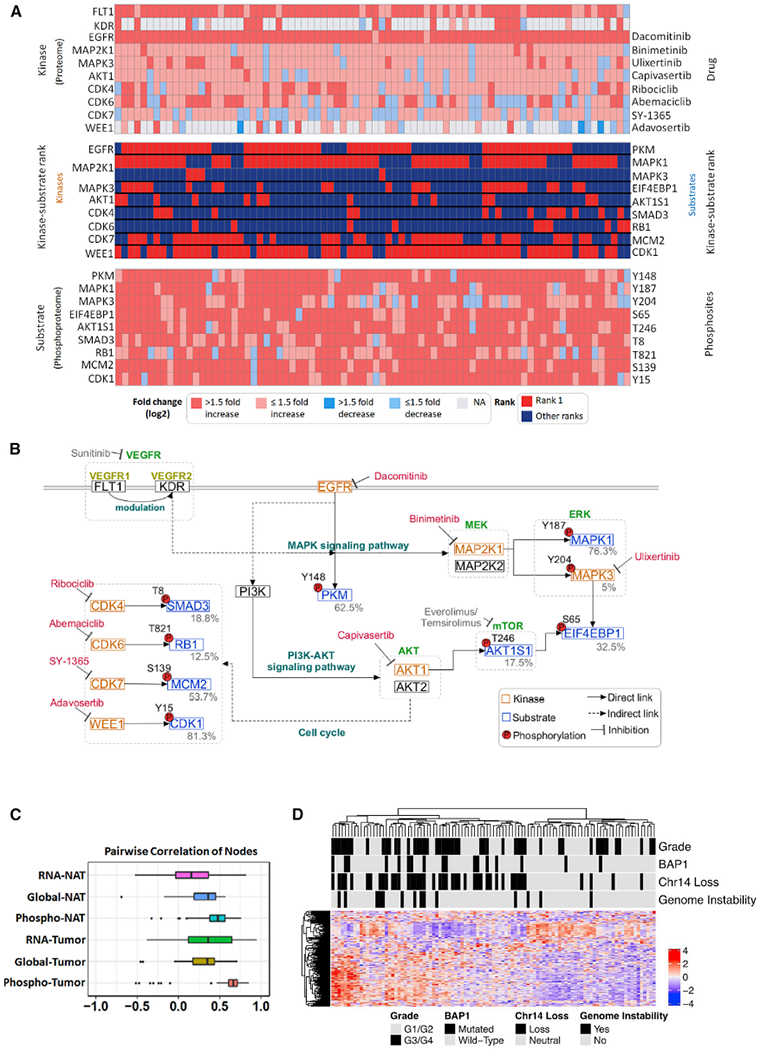
Phospho-Substrates with Associated Kinases and a Network Module Specific to Phospho-Tumor Data (A) Ranked phospho-substrate events of kinases with inhibitors and fold-change at global- and phospho-levels for kinases and substrates, respectively. (B) Pathways based on the selected phospho-substrates and kinases, with relevant drugs shown by targets (red). Current FDA-approved drugs for ccRCC (gray). (C) Pairwise correlation of nodes at multi-omics levels of “cell cycle” co-expression network module. (D) Heatmap of “cell cycle” module expression with grade, BAP1 and chromosome 14 loss, and genome instability distribution annotated. See also Figure S5 and Tables S1 and S6.

Phosphorylation impacts multiple cellular processes, with site occupancy tightly regulated by the activity of kinases and phosphatases on their respective substrates (Ubersax and Ferrell, 2007). We analyzed differential phosphopeptide abundance between 80 tumor/NAT paired tissues to stratify phospho-substrates corresponding to different kinases and their inhibitors, and identified CDK1 and MAPK1 (ERK2) as two highly ranked phospho-substrate events in most tumors. (Figure 5A; Table S6). A more comprehensive investigation of the cell-cycle regulatory network using phosphosite abundance revealed that phosphorylated substrates associated with S-phase entry/progression (CDK7-MCM2) and the G2/M checkpoint (WEE1-CDK1) were elevated across the majority of tumors (Figure 5A). The G2/M checkpoint is the final safeguard of genomic fidelity prior to mitosis; our data support a mechanism of G2-stalling that prevents mitotic arrest-induced apoptosis in tumors (Bucher and Britten, 2008), evidenced by elevated levels of the inhibitory CDK1-Y15 phosphorylation, especially in more aggressive tumors (p < 0.05) (Figure S5B). Comprehensive examination of the signaling network involving MAPK1 revealed increased protein and phospho-peptide expression of the upstream receptor tyrosine kinase epidermal growth factor receptor (EGFR) in almost all tumors, while vascular endothelial growth factor (VEGF) receptors such as FLT and KDR were more selectively expressed and phosphorylated in tumors (Figures 5A and S5A). Additionally, we inferred activated signaling from elevated substrate phosphosite occupancy. This analysis indicated that activation of the EGFR/VEGF downstream signaling pathways MAPK/ERK and AKT-mTOR converged on the downstream substrate EIF4EBP1 (Figures 5A and 5B), an important regulator of protein translation (She et al., 2010). This observation of cell signaling redundancy may explain the limited clinical response of ccRCC patients to mTOR-targeted therapies such as everolimus and temsirolimus (Kwiatkowski et al., 2016), suggesting that combinational therapy targeting both mTOR and MAPK/ERK pathways may be a more effective approach. In addition, activation of mTOR signaling via the phosphorylation of the mTORC1 subunit, AKT1S1 (Vander Haar et al., 2007), was observed in phosphoproteomic analysis but was not captured at the transcriptomic level (Figure S5C), highlighting the added value of phosphoproteomics in integrative analyses. Independent of EGFR-mediated MAPK/AKT signaling, PKM phosphorylation was highly ranked in approximately half of our ccRCC tumor cohort and associated with lower tumor grade (p < 0.05) (Figures 5A and S5B), reflecting a secondary, EGFR-mediated mechanism of glycolytic reprogramming in a subset of ccRCC tumors (Lim et al., 2016).

Leveraging differential phosphopeptide abundance across all tumor samples, we identified several phosphopeptide co-expression networks including two modules (cell cycle and angiogenesis) that were independent of global proteomic and transcriptomic profiles (Figures 5C, 5D, and S5D–S5G; Table S6). The cell-cycle module included multiple cell-cycle checkpoint proteins involved in the G1/S-phase transition (CDKN1B, SKP2), S-phase regulatory elements (MCM4, MCM6), and the G2/M phase (CDK1, TK1, CDC20) (Figure S5D), with phosphorylation of CDC20 representing another mechanism of mitotic-arrest (Hein and Nilsson, 2016). Interestingly, we observed tumors with genomic instability that correlated with this module, as well as phospho-events involved in DNA damage response (e.g., FANCD2, PSME3, CLSPN, and BRCA1) (Figures 5D and S5D), representing a mechanism by which a subset of tumors engage cellular processes in response to loss of genomic fidelity. The angiogenesis module included multiple elements associated with VEGF-response (ELK3, ERG), Notch-associated signaling (LDB2, SOX18), and vasculature development (PECAM-1, CCM2L) (Figure S5E). This module was inversely correlated with *BAP1* and chromosome 14 loss and associated with lower-grade tumors (Figure S5G). Our phosphoproteomic analysis thus identified multiple signal transduction pathways activated in tumors and provided evidence for expanding treatment selection beyond the current FDA-approved therapies targeting VEGF and mTOR (Figure 5B; Hsieh et al., 2018b).

### Characterization of Immune Infiltration in ccRCC

**Figure 6.**
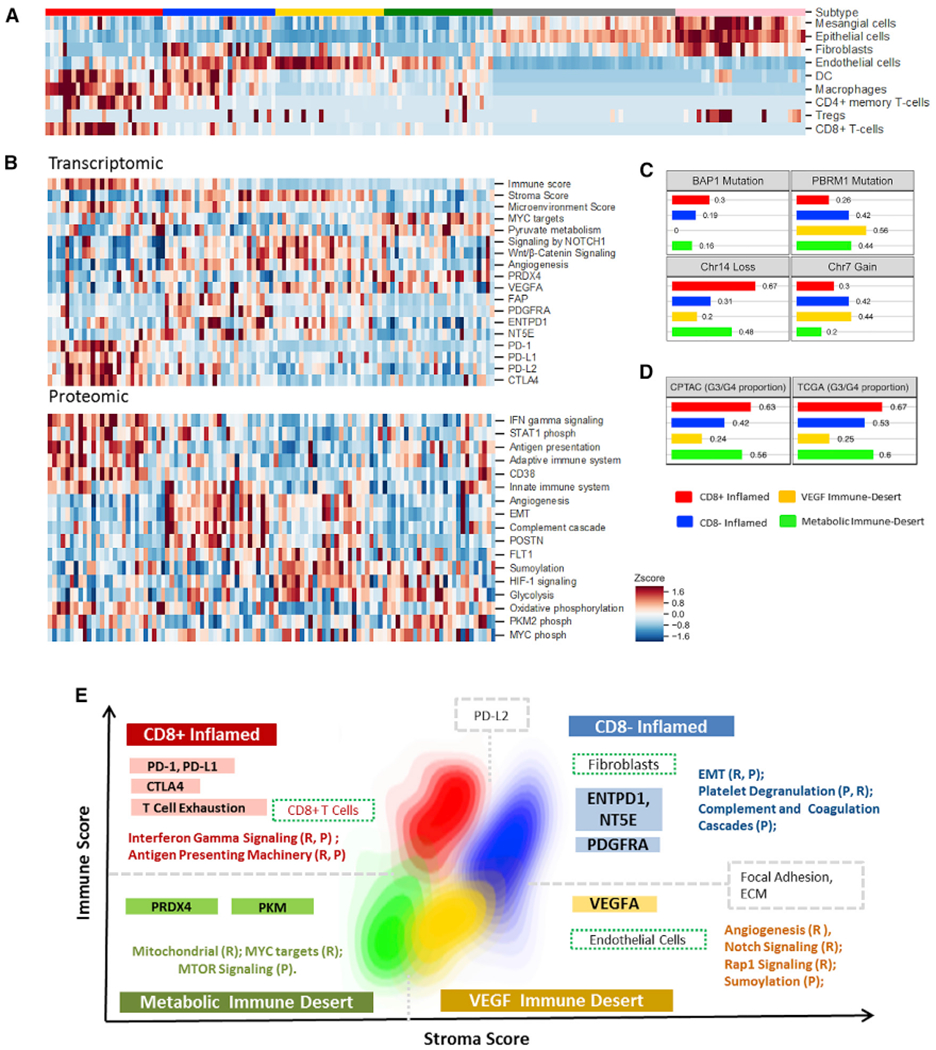
Immune-Based Subtyping of ccRCC Tumors (A) Transcriptome-based deconvolution of mRNA transcript cell signatures in 103 ccRCC tumors and 72 NATs using xCell. (B) Molecular characteristics (transcriptomic, proteomic) stratified tumors into four immune subtypes: CD8^+^ inflamed (red), CD8^−^ inflamed (blue), VEGF immune desert (yellow), metabolic immune desert (green), and NATs into two subtypes (pink and gray). (C) Proportion of *BAP1* mutation, *PBRM1* mutation, chromosome 14 loss, and chromosome 7 gains within each of the immune groups. (D) Proportion of high tumor grade tumors (i.e., grade 3 and grade 4) in each of the immune groups for CPTAC and TCGA datasets. High-grade tumors were significantly enriched in CD8^+^ inflamed group compared to VEGF immune desert group. (E) Density contours of immune and stroma scores of each immune subtype. Pathways upregulated based on RNA-seq and global proteomics data are labeled with “R” or “P,” respectively. See also Figure S6 and Tables S1 and S7.

To gain insight into features of immune infiltration in ccRCC, we analyzed the transcriptomic profiles of 103 tumors and 72 NATs and deconvoluted immune, stromal, and microenvironmental cell gene signatures using xCell (Aran et al., 2017). These molecularly based cell-type classifications were supported by histopathological assessment, DNA promoter methylation-based deconvolution analysis, and ESTIMATE analysis (Yoshihara et al., 2013), with the latter showing a Pearson correlation higher than 0.75 between protein and mRNA data for immune- and stromal-derived signatures (Figures S6A and S6B; Table S7). ESTIMATE generated RNA-seq stromal and immune signatures in this cohort were comparable to those observed in TCGA ccRCC and Genotype-Tissue Expression (GTEx) kidney-cortex datasets (Figure S6C). Consensus clustering of the cell signatures identified two NAT subtypes with distinct enrichment of cell signatures relative to ccRCC tumor tissues and four ccRCC tumor subtypes (Figure 6A). The latter were discriminated by the presence or absence of specific cell types related to immune (CD8^+^ T cells, macrophages, dendritic cells) and stromal (fibroblast, endothelial) signatures. Adopting general features of immune-based groupings described previously (Chen and Mellman, 2017) and incorporating transcriptomic and proteomic features, we defined four tumor subtypes in this ccRCC cohort: (1) CD8^+^ inflamed, (2) CD8^−^ inflamed, (3) VEGF immune desert, and (4) metabolic immune desert (Figures 6B and S6D; Table S7). These subtypes were characterized by unique genomic alterations and tumor microenvironment (TME) signatures and discriminating signaling pathways that could be leveraged to predict therapeutic response (Figures 6B, 6C, and 6E).

CD8^+^ inflamed tumors were characterized by a high degree of CD8^+^ T cell infiltration (t test adjusted p < 0.05) (Figure S6D), increased expression of the immune evasion markers *PD1, PD-L1, PD-L2*, and *CTLA4* (t test adjusted p < 0.05), and high frequency of chromosome 14 loss (chi-square test p < 0.05) (Figures 6A–6C). Corresponding to the elevated CD8^+^ T cell presence was a higher frequency of *BAP1* mutations, a feature previously associated with increased immune infiltration in a kidney cancer xenograft model (Wang et al., 2018b). Proteomic analysis showed upregulation of CD38 expression and pathways involved in antigen processing/presentation (APM) and interferon-γ signaling (Fisher’s exact test adjusted p < 0.05) (Figures 6B and S6E; Table S7). Phosphoproteomic analysis confirmed active interferon-γ signaling via elevated phosphorylation of the downstream effector STAT1 (t test adjusted p < 0.05) (Figure 6B). Interferon-γ signaling has been shown to regulate PD-L1 expression in cancer cells (Chen et al., 2012; Garcia-Diaz et al., 2017), and the combination of increased CD38 protein and mRNA signature (*PD-L1, PD-L2*, and *CTLA4*) associated with T cell exhaustion are representative of multiple mechanisms of immune evasion in this tumor type, with implications for immune checkpoint therapy (Chen et al., 2018; Sade-Feldman et al., 2018).

CD8^−^ inflamed tumors were characterized by an innate immune signature, evidenced by dendritic and macrophage cells in the TME compared to the VEGF and metabolic immune desert groups and by increased complement and coagulation cascade protein expression (Fisher’s exact test adjusted p < 0.05) (Figures 6A, 6B, and S6D; Table S7). A fibroblast signature that included elevated *PDGFRA*, extracellular matrix (ECM) and EMT-associated protein abundance, and expression of cancer associated fibroblasts (CAFs) features (POSTN protein and gene *FAP* mRNA) (t test adjusted p < 0.05) was a unique feature of the CD8^−^ inflamed tumor subtype (Figures 6A, 6B, and S6E; Shiga et al., 2015; Ziani et al., 2018). Together, these associated pathways are representative of TME-tumor crosstalk, with PDGF signaling driving fibroblast recruitment and activation, and CAFs subsequently inducing an EMT-phenotype in tumors (Gascard and Tlsty, 2016; Seppä et al., 1982). Interestingly, *PD-L2* mRNA levels were elevated independent of *PD-L1* expression (Figure 6B), suggesting a CAF-mediated mechanism of T cell death via PD-L2 antigen presentation (Lakins et al., 2018). Independently, increased mRNA expression of *ENTPD1* (*CD39*) and *NT5E* (*CD73*) in the CD8^−^ inflamed tumor subtype could signal an adenosine-rich TME (Antonioli et al., 2013), further contributing to CD8^+^ T cell exclusion in these tumors (Figures 6B and S6D).

CD8^−^ inflamed tumors and VEGF immune desert tumors shared several features including an elevated stromal score (t test adjusted p < 0.05) and enrichment of endothelial cells (t test adjusted p < 0.05 – VEGF immune desert) (Figures 6A and 6B; Table S7). Interestingly, these tumor subtypes had a higher frequency of chromosome 7 gain and lower frequency of chromosome 14 loss, with the latter feature inversely correlated with endothelial cell presence (Figures 6A and 6C). Although angiogenic signaling was elevated in both subtypes (Fisher’s exact test adjusted p < 0.05), angiogenesis and platelet degranulation were higher in CD8^−^ inflamed tumors (Fisher’s exact test adjusted p < 0.05) and corresponded to upregulation of the PDGF-related signaling pathway (Table S7). In contrast, higher expression of SUMOylation (protein level) and Wnt/β-catenin, RAP1, and Notch signaling pathways (mRNA level) were observed in VEGF immune desert tumors and corresponded to the highest endothelial cell signature (t test adjusted p < 0.05) (Figures 6B and S6E). The differential expression of VEGFR1 (FLT1) (t test adjusted p < 0.05) and multiple angiogenic-related signaling pathways may be representative of distinct mechanisms of endothelial cell recruitment and tumor vasculature formation between the two subtypes (Birdsey et al., 2015; Cao, 2013; Chrzanowska-Wodnicka, 2013; Hellström et al., 2007; Zhou et al., 2018; Zhu et al., 2017).

Metabolic immune desert tumors displayed low immune, stromal, and microenvironment scores (t test adjusted p < 0.05), an elevated *MYC* target mRNA signature (Fisher’s exact test adjusted p < 0.05), increased mTOR signaling (Fisher’s exact test adjusted p < 0.05), and a unique metabolic profile that included elevated mitochondrial, OXPHOS, glycolysis protein expression (Fisher’s exact test p < 0.05), and *PKM* mRNA expression (t test adjusted p < 0.05) (Figures 6B and S6E; Table S7). Previous reports have established a relationship between HIF-2α and MYC-induced transcription in renal carcinomas (Gordan et al., 2007a, 2008), with MYC regulation of metabolism functioning in concert with and independent of HIF-1 signaling (Gordan et al., 2007b). Further contributing to this unique metabolism signature was the increased expression of *PRDX4* (t test adjusted p < 0.05) (Figure 6B), which has been shown to impair the binding of HIF-1α/2α to the hypoxia response element in select glycolytic genes (*SLC2A3, PDK3, GPI*) (Luo et al., 2016). The minimal presence of any immune cells in metabolic immune desert tumors is particularly striking and supports the hypothesis that a hypoxic, nutrient-poor microenvironment can be immunosuppressive (Anderson et al., 2017; Mgrditchian et al., 2014).

This analysis discriminated four subtypes of ccRCC and linked unique cellular pathways to observed TME compositions, with select protein features validated using an orthogonal mass spectrometry approach (Figures 6A, 6E, and S6D; Table S7). We hypothesized that the delineated molecular signatures may predict patient responses to select therapies and survival. To examine the former, we characterized tumors within each subtype using two gene signatures that have been previously associated with patient response to immune checkpoint and anti-VEGF therapies (T-effector [T_eff_] and angiogenesis [Angio]), respectively (McDermott et al., 2018). CD8^+^ inflamed tumors displayed an elevated T_eff_ signature relative to other tumor types, while VEGF immune desert tumors displayed an elevated Angio signature (Figure S6F). The remaining two subtypes displayed a minimal T_eff_ signature but had a moderate Angio signature, suggesting a potential response to therapeutics targeting VEGF signaling. Leveraging the gene signatures from our subtypes, we explored the TCGA dataset and observed similar distribution patterns of tumor grade and CD8^+^ T cell, endothelial, and fibroblast cell composition (Figures 6D and S6G; Table S7). Patient stratification based on the four subtypes revealed that VEGF immune desert tumors were associated with improved patient survival, while CD8^+^ Inflamed tumors were associated with poor patient outcome (log-rank test adjusted p < 0.05) (Figure S6H). The latter result reflects the aggregation of multiple features in the CD8^+^ Inflamed subtype that are considered as poor prognosticators in ccRCC, including higher frequency of *BAP1* mutations (chi-square test adjusted p < 0.05), increased proportion of higher grade tumors, and increased *PD-1/PD-L1* expression (t test adjusted p < 0.05). We did not detect an association of tumor mutational burden or neoantigen load with any of these immune subtypes (Table S7), confirming previous reports that indicate that these features do not correlate with ccRCC prognosis (Matsushita et al., 2016; McDermott et al., 2018).

### Proteome Inter-Tumoral Heterogeneity of ccRCC

**Figure 7.**
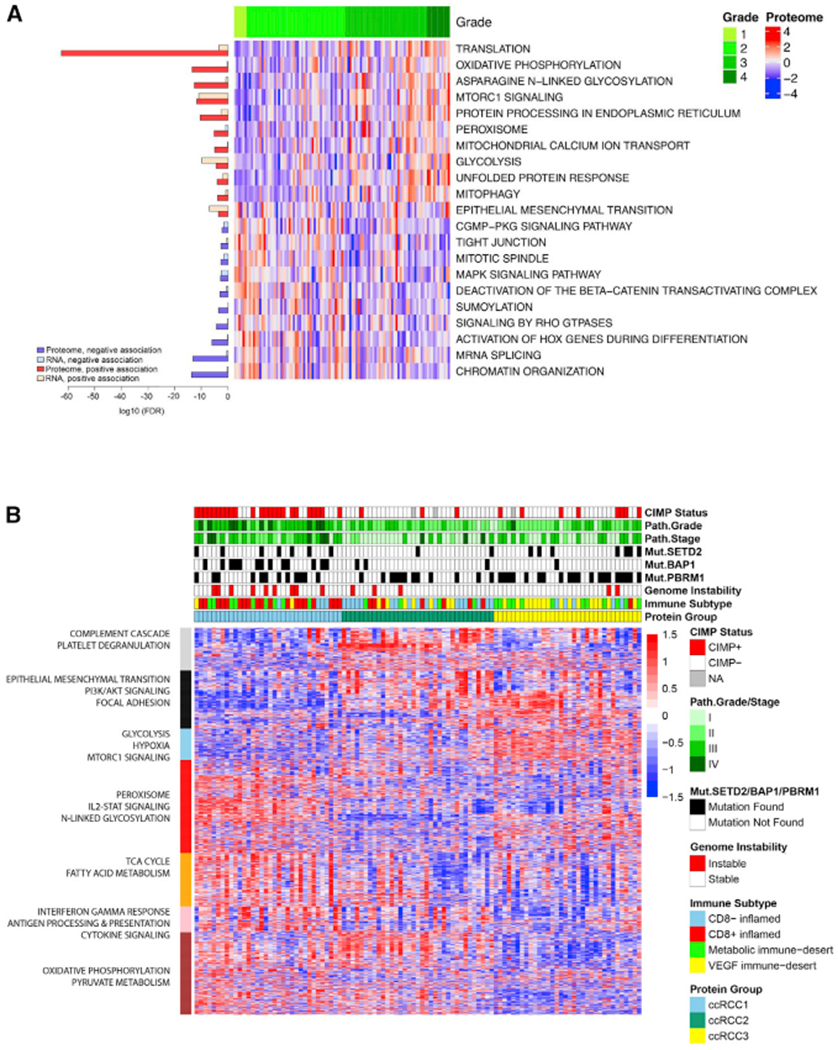
Proteomic Inter-Tumor Heterogeneity of ccRCC and Associated Functional Pathways (A) Cellular pathways (right) with positive (red) or negative (blue) associations with grade (adjusted p < 0.05) at protein or mRNA level (left). Heatmap of protein expression associated with high- and low-grade tumors (center) (Benjamini-Hochberg adjusted p < 0.05). (B) Heatmap of global proteomic abundances. For subtype identification, protein features (n = 3,567) were selected based on highest variance. Color indicates *Z* score of protein in each sample: red is increased, blue is decreased. Clinical and molecular features are indicated above the heatmap. Cluster-derived modules are annotated according to pathway enrichment using Hallmark Gene signature, REACTOME, and KEGG ontologies (adjusted p < 0.05). See also Figure S7 and Tables S1 and S5.

Tumor grade is an important prognosticator in ccRCC, which is often associated with higher-stage and larger tumors (Ishigami et al., 2014). Multivariate analysis integrating clinical features and CPTAC “omics” data found that numerous genes within each data type were associated with immune and stromal scores and with tumor grade (Benjamini-Hochberg adjusted p < 0.10; Figure S7A). Further investigation of these genes revealed distinct pathways associated with tumor grade. In high-grade tumors, translation, mTOR signaling, and EMT were increased at the mRNA and protein levels. Other cellular pathways displayed disparate upregulation at the transcriptomic and proteomic levels. For example, increased mRNA levels were associated with cell-cycle regulation and DNA repair, while increased OXPHOS and *N*-linked glycosylation were detected only at the protein level (Figures 7A and S7B; Table S5). In contrast, low-grade tumors displayed increased expression of receptor tyrosine kinase, RAS, MAPK, Notch, and RAP1 cell signaling pathways at both the mRNA and protein levels (Wilcoxon rank-sum test, Benjamini-Hochberg adjusted p < 0.01; Figures 7A and S7B; Table S5). Low-grade tumors showed increased protein expression of transcription-related processes (e.g., chromatin reorganization) that was not captured at the transcript level (Wilcoxon rank-sum test, Benjamini-Hochberg adjusted p < 0.01; Figure 7A; Table S5). The divergence of cellular processes between high- and low-grade tumors reflects the disparate tumoral needs, with higher grade tumors upregulating mechanisms to adapt to increased genomic alterations and a changing tumor microenvironment.

To determine ccRCC inter-tumor heterogeneity of the CPTAC cohort, we constructed an unbiased proteomic grouping of ccRCC. Three major proteomic ccRCC groups emerged from this analysis (ccRCC1-3), which were discriminated by seven major protein clusters (Figure 7B; Table S5). Tumors in ccRCC2 had a higher degree of protein expression associated with innate immunity and platelet degranulation (adjusted p < 0.05), while those in ccRCC3 displayed increased protein expression associated with glycolysis, mTOR signaling, and hypoxia (adjusted p < 0.05). ccRCC2 and ccRCC3 were associated with lower tumor grade (p < 0.01 and p < 0.02, respectively), while only ccRCC2 was associated with lower stage (p < 0.001). Tumors in ccRCC1 presented with higher grade (p < 0.001) and stage (p < 0.01), characterized by elevated adaptive immune response, *N*-linked glycosylation, OXPHOS protein expression, and fatty acid metabolism (adjusted p < 0.05). Tumors in ccRCC3 displayed a higher frequency of *PBRM1* mutations (p < 0.05), whereas those in ccRCC1 had a higher frequency of *BAP1* mutations (p < 0.0001), CIMP^+^ status (p < 0.007), and genomic instability (p < 0.0001) (Figure 7B). As highlighted in our immune analysis, ccRCC tumors had variable TME compositions, with immune and stromal signatures impacting observed protein expression patterns (Figure S7A). The distribution of the immune subtypes across the proteomic groupings contributed to the discriminating gene clusters described in Figure 7B that were also delineated in the immune-based subtyping of ccRCC (Figure 6B), capturing the more dominant molecular signatures of CD8^+^ inflamed (interferon-γ signaling), CD8^−^ inflamed (platelet degranulation), and VEGF immune desert (hypoxic signaling) tumors, which had a higher prevalence in ccRCC1 (p < 5.0 e–07), ccRCC2 (p < 6.0 e–05), and ccRCC3 (p < 0.0001), respectively.

## DISCUSSION

This comprehensive proteogenomic characterization of ccRCC provides insight into the differential impact of underlying genomic and epigenomic events on the transcriptome, proteome, and phosphoproteome. The integration of chromosome-level alterations and comparative, multi-level profiling of treatment-naive tumors and NATs connects genomic aberrations to active mechanisms that drive ccRCC tumorigenesis. The identification of a subset of ccRCC patients who display genomic instability could have clinical utility that warrants further investigation, as this group may have worse prognosis and benefit from continual surveillance post-treatment. The *trans*-effects of chromosome 3p fully capture the dysregulated VHL/HIF-1 axis that is a hallmark of ccRCC, while the widespread translocation events involving chromosome 3 observed in this study and others (Mitchell et al., 2018; Pavlovich et al., 2003) portray ccRCC as a disorder defined by genomic rearrangements.

Our analysis supports and elaborates on the metabolic shift that occurs within ccRCC tumors, illustrated at the protein level by the upregulation of glycolysis and the corresponding downregulation of the Krebs cycle and the electron transport chain (OXPHOS) associated with the Warburg effect. Notably, the downregulation of components of the Krebs cycle and the majority of the nuclear-encoded OXPHOS proteins were not observed at the mRNA level and would have not been reported by RNA-seq analysis alone. This finding is significant, as recent large-scale ccRCC studies have focused on mRNA expression data to depict the metabolic shift in ccRCC and have evaluated transcriptomic signatures to stratify patients with more aggressive disease (Chen et al., 2016a; Creighton et al., 2013). HIF1-signaling has been shown to decrease OXPHOS activity through multiple mechanisms (Hervouet et al., 2008; Papandreou et al., 2006). Interestingly, our proteomic analysis and previous metabolic profiling of ccRCC show evidence of late-stage tumors upregulating the OXPHOS pathway relative to earlier-stage tumors (Hakimi et al., 2016) and may reflect the dysregulation of HIF-1α expression resulting from 14q loss or the aberrant methylation profiles associated with CIMP^+^ status. The maintenance of OXPHOS transcription levels similar to those seen in normoxic cells may provide a mechanism for rapid induction of OXPHOS activity when it is advantageous to fulfill tumor energy requirements. This hypothesis warrants deeper exploration and expanded investigation in other cancer types.

Current first-line therapies for advanced ccRCC target VEGF and mTOR (Escudier et al., 2007a, 2007b; Hudes et al., 2007; Motzer et al., 2007, 2008; Sternberg et al., 2010), with ongoing exploration of immune checkpoint inhibitors (Atkins and Tannir, 2018). Through investigation of transcriptomic, proteomic, and phosphoproteomic signatures of treatment-naive tumors, we propose a rational stratification of ccRCC patients for personalized therapeutic interventions. Immune-based subtyping of ccRCC tumors via deconvolution of cell composition identified TME and cellular pathways that delineated patients who displayed a pro-angiogenic phenotype from those with an immune-evasive phenotype. With evidence demonstrating the lack of efficacy of anti-VEGF therapy in patients with elevated levels of immune checkpoint signatures (Hara et al., 2017; Shin et al., 2015) and preliminary clinical studies showing encouraging results when treating RCC using PD-1/CTLA4-targeting therapies (Motzer et al., 2018), it is vital to identify theranostic markers to improve patient outcome and overall survival. However, mechanisms that drive the observed differences in TME signatures warrant further exploration. Our results support recent reports that tumor mutational burden or neoantigen load may not be associated with immune infiltration or response to immune checkpoint therapy in ccRCC (Matsushita et al., 2016; McDermott et al., 2018), although alternative explanations, such as endogenous retroviral expression, were not examined in our study (Panda et al., 2018; Smith et al., 2018). Independent of current first-line regimens and immune checkpoint inhibition, the ubiquitous activation of EGFR and downstream signaling cascades (MAPK1), as well as cell-cycle checkpoint regulation (WEE1-CDK1) revealed by our phosphoproteomic analysis, provide additional therapeutic targets that have been evaluated extensively in other cancer types but minimally in ccRCC (Ascierto et al., 2013; Huang et al., 2008; Matheson et al., 2016; Ravaud et al., 2008). Application to ccRCC would be especially important given our identification of a subset of ccRCC tumors that are predicted or shown to be immune checkpoint/VEGF non-responders (Beuselinck et al., 2015; Maroto et al., 2017) that may benefit from therapies that activate anti-tumor T cell expansion (Naing et al., 2018) or combinatorial therapeutic approaches, such as concurrent cell-cycle checkpoint and mTOR inhibition.

Overall, this study reveals unique biological insights that are gained only when combining complementary proteomic and genomic analyses that link the functional consequences of genomic aberrations with proteomic outcomes. The integration of comprehensive genomic, epigenomic, transcriptomic, proteomic, and phosphoproteomic measurements for tumors and corresponding NATs provides an invaluable bioinformatic resource for the deeper examination of ccRCC tumorigenesis. Our multi-level “omics” analysis identifies underlying molecular mechanisms that are not fully captured at the genomic and transcriptomic levels and defines proteomic, phosphoproteomic, and immune signatures necessary to stratify ccRCC patients, with the goal of developing rational therapeutic interventions.

## STAR★METHODS

### LEAD CONTACT AND MATERIALS AVAILABILITY

This study did not generate new unique reagents. Further information and requests for resources should be directed to and will be fulfilled by the Lead Contact, Hui Zhang (huizhang@jhu.edu).

### EXPERIMENTAL MODEL AND SUBJECT DETAILS

#### Human Subjects

A total of 110 participants, with an age range of 30-90, were included in this study. This cohort contained males (n = 81) and females (n = 29) and reflects the gender distribution of clear cell renal cell carcinoma (ccRCC) (Creighton et al., 2013). Only histopathologically defined adult ccRCC tumors were only included in the analysis. Institutional review boards at each Tissue Source Site (TSS) reviewed protocols and consent documentation, in adherence to Clinical Proteomic Tumor Analysis Consortium (CPTAC) guidelines.

#### Clinical Data Annotation

Clinical data were obtained from TSS and aggregated by the Biospecimen Core Resource (BCR, Van Andel Research Institute (Grand Rapids, MI)). Data forms were stored as Microsoft Excel files (.xls). Clinical data can be accessed and downloaded from the CPTAC Data Portal at https://cptac-data-portal.georgetown.edu/cptac/documents/S044_CPTAC_CCRCC_Discovery_Cohort_Clinical_Data_r1_Sept2018.xlsx. Patients with any prior history of other malignancies within twelve months or any systemic treatment (chemotherapy, radiotherapy, of immune-related therapy) were excluded from this study. Demographics, histopathologic information, and treatment details were collected and summarized in Table S1. The characteristics of the CPTAC ccRCC cohort reflect the general incidence of ccRCC (Creighton et al., 2013), including patient age distributions [30-49 (17.4%), 50-69 (60.6%), and > 70 (22.0%)], grade distributions [G1 (5.5%), G2 (48.6%), G3 (37.6%), and G4 (8.3%)], and stage distributions [I (46.8%), II (11.9%), III (30.3%), and IV (11.0%)].

### METHOD DETAILS

#### Specimen Acquisition

The tumor, adjacent normal tissue and whole blood samples used in this manuscript were prospectively collected for the CPTAC project. Biospecimens were collected from newly-diagnosed patients with ccRCC who were undergoing surgical resection and had received no prior treatment for their disease, including radiotherapy or chemotherapy. All cases had ccRCC histology but were collected regardless of histologic grade or surgical stage. Cases were then graded using the Fuhrman Nuclear Grading System and staged using the AJCC cancer staging system 7^th^ edition (Edge et al., 2010). Tumors specimens weighed between 125 and 3,000 mg. For most cases, three to four tumor specimens were collected. Each tissue specimen endured cold ischemia for 30 minutes or less prior to freezing in liquid nitrogen. The specimens were collected with an average total ischemic time of fifteen minutes from resection/collection to freezing. Specimens were either flash-frozen in liquid nitrogen or embedded in optimal cutting temperature (OCT) medium, with histologic sections obtained from top and bottom portions for review. Each case was reviewed by a board-certified pathologist to confirm the assigned pathology. The top and bottom sections were required to contain an average of 80% tumor cell nuclei with less than 20% necrosis. Specimens were shipped overnight from the TSS to the BCR using a cryoport that maintained an average temperature of less than −140°C. At the BCR, the specimens were confirmed by pathology qualification and prepared for genomic, transcriptomic, and proteomic analyses. Selected specimens were cryopulverized, and material was aliquoted for subsequent molecular characterization. Genomic DNA and total RNA were extracted and sent to the genome characterization centers (GCC). The DNA sequencing and methylation analyses were performed at the Broad Institute (Cambridge, MA) and RNA sequencing was performed at the University of North Carolina (Chapel Hill, NC). Material for proteomic analyses was sent to the Proteomic Characterization Center (PCC) at Johns Hopkins Medical Institutions of Johns Hopkins University (Baltimore, MD)

#### Genomic and Transcriptomic Sample Preparation and Data Acquisition

##### Sample Processing for Genomic DNA and total RNA Extraction

Our study sampled a single site of the primary tumor. All DNA and RNA were isolated using a co-isolation protocol in which nucleic acids were isolated from the same cryopulverized aliquot that was used for both proteomics and genomics. Tumor samples were from surgical resections due to the requirement to process a minimum of 125mg of tumor issue and 50mg of adjacent normal tissue. RNA and DNA were extracted from tumor and adjacent normal specimens using QIAGEN’s QIAsymphony DNA Mini Kit and QIAsymphony RNA Kit. Genomic DNA was also isolated from peripheral blood (3-5 mL) to serve as matched benign reference material. The Qubit dsDNA BR Assay Kit was used with the Qubit® 2.0 Fluorometer to determine the concentration of dsDNA in an aqueous solution. A sample that passed quality control and produced sufficient DNA yield various genomic assays was sent for genomic characterization. RNA quality was quantified using the NanoDrop 8000 and quality was assessed using Agilent Bioanalyzer. A sample that passed RNA quality control and had a minimum RNA integrity Number (RIN) score of 8 was subjected to RNA sequencing.

#### Whole Exome Sequencing Methods

##### Library Construction

Library construction was performed as described in Fisher et al. (2011), with the following modifications: initial genomic DNA input into shearing was reduced from 3 μg to 250 ng in 50 μL of solution. For adaptor ligation, Illumina paired end adapters were replaced with palindromic forked adapters obtained from Integrated DNA Technologies, with unique dual-indexed molecular barcode sequences to facilitate downstream pooling. Kapa HyperPrep reagents were used in a 96-reaction kit format for end repair/A-tailing, adaptor ligation, and library enrichment PCR. In addition, during the post-enrichment SPRI cleanup, elution volume was reduced to 30 μL to maximize library concentration followed by a vortexing step to maximize the amount of template eluted.

##### In-solution Hybrid Selection

Following library construction, products were pooled into groups of up to 96 samples. Hybridization and capture were performed using the relevant components of Illumina’s Nextera Exome Kit and following the manufacturer’s recommended protocol, with a few exceptions. First, all libraries within a library construction plate were pooled prior to hybridization. Second, to facilitate automation the Midi plate from Illumina’s Nextera Exome Kit was replaced with a skirted PCR plate. All hybridization and capture steps were automated utilizing the Agilent Bravo liquid handling system.

##### Preparation of Libraries for Cluster Amplification and Sequencing

After post-capture enrichment, library pools were quantified by qPCR using a kit obtained from KAPA Biosystems with probes specific to the ends of the adapters. The assay was automated on the Agilent Bravo liquid handling system. Based on qPCR quantification, libraries were normalized to 2 nM.

##### Cluster Amplification and Sequencing

Cluster amplification of DNA libraries was performed according to the manufacturer’s protocol (Illumina) using exclusion amplification chemistry and flowcells. Flowcells were sequenced utilizing Sequencing-by-Synthesis chemistry. The flowcells are then analyzed using RTA v.2.7.3 or a later version. Each pool of whole exome libraries was sequenced on paired 76-cycle runs with two eight-cycle index reads across the number of lanes necessary to meet coverage for all libraries in the pool. Pooled libraries were processed using HiSeq4000 as paired end runs to achieve a minimum of 150x on-target coverage per library. The raw Illumina sequence data were demultiplexed and converted to FASTQ files, and adaptor and low-quality sequences were trimmed. The raw reads were mapped to the hg38 human reference genome. The validated Binary Alignment Map (BAM) files were used for downstream analysis and variant calling. FASTQ files of all reads were then uploaded to the Genomic Data Commons (GDC) repository.

#### Whole Genome Sequencing, PCR-Free

##### Preparation of Libraries for Cluster Amplification and Sequencing

Input genomic DNA (350 ng in 50 μL) was acoustically sheared using a Covaris focused-ultrasonicator (~385 bp fragment range). Following shearing, an additional size selection was performed using a SPRI cleanup. Library preparation was performed using KAPA Hyper Prep without amplification module (KAPA Biosystems) with palindromic forked adapters containing unique eight-base index sequences embedded within the adaptor (Integrated DNA Technologies). Libraries were quantified using quantitative PCR (KAPA Biosystems), with probes specific to the ends of the adapters. The assay was automated on the Agilent Bravo liquid handling system. Based on qPCR quantification, libraries were normalized to 1.7 nM and pooled into 24-plexes.

##### Cluster Amplification and Sequencing (HiSeqX)

Sample pools were combined with HiSeqX Cluster Amp Regents EPX1, EPX2 and EPX3 into single wells on a strip tube using the Hamilton Starlet Liquid Handling system. Cluster amplification of the templates was performed according to the manufacturer’s protocol (Illumina) with the Illumina cBot. Flowcells were sequenced for a minimum of 15x coverage on HiSeqX utilizing sequencing-by-synthesis to produce 151 base pair paired-end reads. Outputs from Illumina software were processed by the Picard data-processing pipeline to yield BAM files containing demultiplexed, aggregated aligned reads. All sample information tracking was performed by automated LIMS messaging. FASTQ files of all reads were then uploaded to the GDC.

##### Illumina Infinium MethylationEPIC BeadChip Array

The MethylationEPIC array uses an eight-sample version of the Illumina BeadChip that captures > 850,000 methylation sites per sample. 250 ng of DNA was used for bisulfite conversion using the Infinium MethylationEPIC BeadChip Kit. The EPIC array includes sample plating, bisulfite conversion, and methylation array processing. After scanning, the data were processed through an automated genotype calling pipeline, generating raw idat files and a sample sheet. For 109 out of the 110 samples, a sufficient amount of material was obtained to perform methylation profiling. Two out of the 109 samples showed a missing rate greater than 95% and were excluded from all downstream analyses.

#### RNA Sequencing

##### Quantitation and Quality Assessment QC of total RNA Samples

All RNA analytes were assayed for RNA integrity, concentration, and fragment size. Samples for total RNA-seq were quantified on a TapeStation system (Agilent). Samples with RINs > 8.0 were considered to be of high quality and were processed further.

##### Total RNA-seq Library Construction

Total RNA-seq libraries were generated using 300 ng of total RNA, analyzed using the TruSeq Stranded Total RNA Library Prep Kit with Ribo-Zero Gold and bar-coded with individual tags following the manufacturer’s instructions (Illumina). Libraries were prepared on an Agilent Bravo Automated Liquid Handling System. Quality control was performed at every step, and the libraries were quantified using a TapeStation system.

##### Total RNA Sequencing

Indexed RNA-seq libraries were sequenced using the HiSeq4000 platform to generate a minimum of 120 million paired end reads (75 base pairs) per library with a target of greater than 90% mapped reads. The sequence data were demultiplexed and converted to FASTQ files, and adaptor and low-quality sequences were quantified/trimmed. Samples were then assessed for quality by mapping reads to the hg38 reference genome, estimating the total number of reads that mapped, assessing the amount of RNA that mapped to coding regions, the amount of rRNA in the sample, the number of genes expressed, and the relative expression of housekeeping genes. Samples that passed the quality criteria were then clustered with other expression data from similar and distinct tumor types to confirm expected expression patterns, including pathological status (i.e., normal adjacent versus tumor tissue) and tissue-origin specificity. FASTQ files of all reads were then uploaded to the GDC repository.

#### Proteomic Sample Preparation and Data Acquisition

##### Sample Processing for Protein Extraction and Tryptic Digestion

All samples for the current study were prospectively collected as described above and processed for mass spectrometric (MS) analysis at the PCC. Tissue lysis and downstream sample preparation for global proteomic and phosphoproteomic analysis were carried out as previously described (Mertins et al., 2018). Approximately 25-120 mg of each cryopulverized renal tumor tissues or NATs were homogenized separately in an appropriate volume of lysis buffer (8 M urea, 75 mM NaCl, 50 mM Tris, pH 8.0, 1 mM EDTA, 2 μg/mL aprotinin, 10 μg/mL leupeptin, 1 mM PMSF, 10 mM NaF, Phosphatase Inhibitor Cocktail 2 and Phosphatase Inhibitor Cocktail 3 [1:100 dilution], and 20 mM PUGNAc) by repeated vortexing. Lysates were clarified by centrifugation at 20,000 x g for 10 min at 4°C, and protein concentrations determined by BCA assay (Pierce). Lysates were diluted to a final concentration of 8 mg/mL with lysis buffer, and 800 μg of protein was reduced with 5 mM dithiothreitol (DTT) for 1 h at 37°C and subsequently alkylated with 10 mM iodoacetamide for 45 min at RT (room temperature) in the dark. Samples were diluted 1:3 with 50 mM Tris-HCl (pH 8.0) and subjected to proteolytic digestion with LysC (Wako Chemicals) at 1 mAU:50 μg enzyme-to-substrate ratio for 2 h at RT, followed by the addition of sequencing-grade modified trypsin (Promega) at a 1:50 enzyme-to-substrate ratio and overnight incubation at RT. The digested samples were then acidified with 50% trifluoroacetic acid (TFA, Sigma) to a pH value of approximately 2.0. Tryptic peptides were desalted on reversed-phase C18 SPE columns (Waters) and dried using a Speed-Vac (Thermo Scientific).

##### TMT Labeling of Peptides

Tandem-mass-tag (TMT) quantitation utilizes reporter ion intensities to determine protein abundance and facilitate quantitative proteomic analysis (Ross et al., 2004). Previously, CPTAC used two major LC-MS/MS-based methods for quantitative proteomics: label-free quantification (LFQ) and isobaric tag for relative and absolute quantitation (iTRAQ) for proteogenomic characterization of colorectal, breast, and ovarian cancers (Mertins et al., 2016; Zhang et al., 2014a, 2016). Inherent benefits of isobaric tag approaches over LFQ for protein quantitation include decreasing peptide/protein quantification differences attributed to variation in instrument performance, reducing the number “missing values,” and facilitating integrated measurements of global protein and post-transcriptional modifications (PTM) levels (Hogrebe et al., 2018; Thompson et al., 2003). Recent developments in isobaric tag technology have enabled an increase in the number of independent samples that can be analyzed in parallel, thereby, increasing throughput and facilitating deeper quantification of respective proteomes (McAlister et al., 2012; Werner et al., 2012). Desalted peptides from each sample were labeled with 10-plex TMT (Tandem Mass Tag) reagents (Thermo Fisher Scientific). Peptides (300 μg) from each ccRCC and NAT sample were dissolved in 300 μL of 50 mM HEPES, pH 8.5 solution. Five milligrams of TMT reagent was dissolved in 256 μL of anhydrous acetonitrile, and 123 μL of each TMT reagent was added to the corresponding aliquot of peptides. After 1 h incubation at RT, the reaction was quenched by acidification with 50% TFA to pH < 3. A reference sample was created by pooling an aliquot from individual ccRCC tumors and NAT samples (90 tumors and 72 NATs, representing ~90% of the sample cohort), labeled with the TMT-131 reagent, and included in all TMT 10-plexes as a pooled reference channel. Two internal quality control (QC) samples, a single, independently-acquired chromophobe renal cell carcinoma (chRCC) tumor sample and an NCI-7 Cell Line Panel sample (Clark et al., 2018), were prepared and interspersed among all TMT 10-plex sets. 110 ccRCC tumor and 84 NAT samples with eight chromophobe QC aliquots and five NCI-7 QC aliquots were co-randomized to 23 TMT 10-plex sets. The sample-to-TMT channel mapping is shown in https://cptac-data-portal.georgetown.edu/cptac/documents/S044_CPTAC_CCRCC_Discovery_Cohort_Specimens_r1_Sept2018.xlsx . Following labeling, peptides were mixed according to the sample-to-TMT channel mapping, concentrated and desalted on reversed-phase C18 SPE columns (Waters) and dried using a Speed-Vac (Thermo Scientific).

##### Peptide Fractionation by Basic Reversed-phase Liquid Chromatography (bRPLC)

To reduce the likelihood of peptides co-isolating and co-fragmenting in these highly complex samples, we employed extensive, high-resolution fractionation via basic reversed-phase liquid chromatography (bRPLC). Previous reports indicate that this approach can reduce the incidence of isobaric reporter ion ratio distortion effects, which would impact downstream quantitation (Ow et al., 2011; Rauniyar and Yates, 2014). The desalted, TMT-labeled samples were reconstituted in 900 μL of 20 mM ammonium formate (pH 10) and 2% acetonitrile (ACN) and loaded onto a 4.6 mm x 250 mm RP Zorbax 300 A Extend-C18 column with 3.5 μm size beads (Agilent). Peptides were separated at a flow-rate of 1mL/min using an Agilent 1200 Series HPLC instrument via bHPLC with Solvent A (2% ACN, 5 mM ammonium formate, pH 10) and a non-linear gradient of Solvent B (90% ACN, 5 mM ammonium formate, pH 10) as follows: 0% Solvent B (9 min), 6% Solvent B (4 min), 6% to 28.5% Solvent B (50 min), 28.% to 34% Solvent B (5.5 min), 34% to 60% Solvent B (13 min), and holding at 60% Solvent B for 8.5 min. Collected fractions were concatenated into 24 fractions by combining four fractions that are 24 fractions apart (i.e., combining fractions #1, #25, #49, and #73; #2, #26, #50, and #74; and so on); a 5% aliquot of each of the 24 fractions was used for global proteomic analysis, dried in a Speed-Vac, and resuspended in 3% ACN/0.1% formic acid prior to ESI-LC-MS/MS analysis. The remaining sample was utilized for phosphopeptide enrichment.

##### Enrichment of Phosphopeptides by Fe-IMAC

The remaining 95% of the sample was further concatenated before being subjected to phosphopeptide enrichment using immobilized metal affinity chromatography (IMAC) as previously described (Mertins et al., 2013). In brief, Ni-NTA agarose beads were used to prepare Fe^3+^-NTA agarose beads, and 300 mg of peptides were reconstituted in 80% ACN/0.1% trifluoroacetic acid and incubated with 10 μL of the Fe^3+^-IMAC beads for 30 min. Samples were then centrifuged, and the supernatant containing unbound peptides was removed. The beads were washed twice and then transferred onto equilibrated C-18 Stage Tips with 80% ACN/0.1% trifluoroacetic acid. Tips were rinsed twice with 1% formic acid and eluted from the Fe^3+^-IMAC beads onto the C-18 Stage Tips with 70 μL of 500 mM dibasic potassium phosphate, pH 7.0 a total of three times. C-18 Stage Tips were then washed twice with 1% formic acid, followed by elution of the phosphopeptides from the C-18 Stage Tips with 50% ACN/0.1% formic acid twice. Samples were dried down and resuspended in 3% ACN/0.1% formic acid prior to ESI-LC-MS/MS analysis.

##### ESI-LC-MS/MS for Global Proteome and Phosphoproteome Analysis

Global proteome and phosphoproteome fractions were analyzed using the same instrumentation and methodology. Peptides (~0.8 μg) were separated on an Easy nLC 1200 UHPLC system (Thermo Scientific) on an in-house packed 20 cm x 75 mm diameter C18 column (1.9 mm Reprosil-Pur C18-AQ beads (Dr. Maisch GmbH); Picofrit 10 mm opening (New Objective)). The column was heated to 50°C using a column heater (Phoenix-ST). The flow rate was 0.200 μl/min with 0.1% formic acid and 2% acetonitrile in water (A) and 0.1% formic acid, 90% acetonitrile (B). The peptides were separated with a 6%–30% B gradient in 84 min and analyzed using the Thermo Fusion Lumos mass spectrometer (Thermo Scientific). Parameters were as follows: MS1: resolution – 60,000, mass range – 350 to 1800 m/z, RF Lens – 30%, AGC Target 4.0e^5^, Max IT – 50 ms, charge state include - 2-6, dynamic exclusion – 45 s, top 20 ions selected for MS2; MS2: resolution-50,000, high-energy collision dissociation activation energy (HCD)-37, isolation width (m/z) – 0.7, AGC Target – 2.0e^5^, Max IT – 105 ms.

##### ESI-LC-MS/MS for Global Proteome Data-Independent Acquisition Analysis

Unlabeled, digested peptide material from individual tissue samples (ccRCC and NAT) was spiked with index Retention Time (iRT) peptides (Biognosys) and subjected to data-independent acquisition (DIA) analysis. Peptides (~0.8 μg) were separated on an Easy nLC 1200 UHPLC system (Thermo Scientific) on an in-house packed 20 cm x 75 μm diameter C18 column (1.9 μm Reprosil-Pur C18-AQ beads (Dr. Maisch GmbH); Picofrit 10 μm opening (New Objective)). The column was heated to 50°C using a column heater (Phoenix-ST). The flow rate was 0.200 μl/min with 0.1% formic acid and 3% acetonitrile in water (A) and 0.1% formic acid, 90% acetonitrile (B). The peptides were separated with a 7%–30% B gradient in 84 mins and analyzed using the Thermo Fusion Lumos mass spectrometer (Thermo Scientific). The DIA segment consisted of one MS1 scan (350-1650 m/z range, 120K resolution) followed by 30 MS2 scans (variable m/z range, 30K resolution). Additional parameters were as follows: MS1: RF Lens – 30%, AGC Target 4.0e^5^, Max IT – 50 ms, charge state include - 2-6; MS2: isolation width (m/z) – 0.7, AGC Target - 2.0e^5^, Max IT – 120 ms.

##### Spectral Library generation for Data-Independent Acquisition Analysis

For spectral library generation, an aliquot (2 μg) of unlabeled, digested peptide material from individual tissue samples (ccRCC and NAT) was pooled and subjected to bRPLC as previously described. Collected fractions were concatenated into eight fractions by combining twelve fractions that are eight fractions apart (i.e., combining fractions #1, #9, #17, #25, #33, #41, #49, #57, #65, #73, #81, and #89; #2, #18, #26, #34, #42, #58, #66, #74, #82, and #90; and so on); dried down in a Speed-Vac, resuspended in 3% ACN, 0.1% formic acid, and was spiked with index Retention Time (iRT) peptides (Biognosys) prior to ESI-LC-MS/MS analysis. Parameters were the same as previously described for ESI-LC-MS/MS for Global Proteome and Phosphoproteome Analysis with a high-energy collision dissociation activation energy (HCD) – 34.

#### Genomic Data Processing

##### Harmonized Somatic Variant Calling

Details regarding somatic variant calling performed by the GDC are available at: https://docs.gdc.cancer.gov/Data/Bioinformatics_Pipelines/DNA_Seq_Variant_Calling_Pipeline/, https://gdc.cancer.gov/about-gdc/variant-calling-gdc. The University of Michigan aligned FASTQ files to the GRCh38 references, including alternate haplotypes. Variant calling was performed using VarDict (germline & somatic) and Strelka2 (somatic). Variant callers were run with default settings, but custom filters were applied. Strelka was used to generate the primary somatic call-set. Variants called by Strelka had to be either (FILTER = = “PASS”) or meet the following threshold criteria: allele frequency in the tumor > 0.05, allele frequency in the normal < 0.01, at least five variant reads, depth in normal > 50, Somatic Evidence Score (EVS) > 90th percentile of overall EVS distribution. These calls were supplemented by variants called confidently (FILTER = = “PASS” and manual review) by VarDict in genes recurrently mutated in ccRCC: *VHL, PBRM1, BAP1, SETD2, KDM5C, PTEN, MTOR, TP53, PIK3CA, ARID1A, STAG2, KDM6A, KMT2C, KMT2D.* This strategy improved sensitivity in ccRCC-mutated genes without sacrificing the accuracy of variant calls genome wide. Washington University in St. Louis called somatic variants using four tools: Strelka2, Mutect2, VarScan2.3.8, and Pindel0.2.5. SNVs and indels from the four tools were then merged with SNVs identified by the University of Michigan and GDC pipelines using the following strategy: SNVs called by any two callers among Mutect2, VarScan2.3.8, and Strelka2 and indels called by any two callers among MUTECT2, VarScan2.3.8, Strelka2, and Pindel 0.2.5. For the merged SNVs and indels, we applied a cut-off of 14X and 8X coverage for tumor and normal respectively. SNVs and indels were filtered using a minimal variant allele frequency (VAF) of 0.05 in tumors and a maximal VAF of 0.02 in normal tissues. Any SNV within 10 bps of an indel identified in the same tumor sample was filtered.

##### Structural Variant Analysis

Structural variants (SVs) and indels were called from the whole-genome mapped paired-end sequencing reads by Manta (Chen et al., 2016b) with the default record- and sample-level filters. Record-level filters included a QUAL score < 20; somatic variant quality score < 30; depth greater than 3x the median chromosome depth near one or both variant breakends; for variants significantly larger than the paired read fragment size, no paired reads support the alternate allele in any sample). Sample-level filters included a Genotype Quality <15. This approach optimizes the analysis of somatic variation in tumor/normal sample pairs. The paired and split-read evidence was combined during the SV discovery and scoring to improve accuracy but did not require split-reads or successful breakpoint assemblies to report a variant in cases where there is strong evidence otherwise. Calls were prioritized with three confidence levels based on whether the read evidence included spanning read pairs (level 1: no spanning read pairs, only split-reads; level 2: one spanning read pair with or without split-reads; level 3: two or more spanning read pairs with or without split-reads) (Table S2). We mainly focused on levels 2 and 3 SVs with spanning read pair evidence. We extracted the chr3 translocation events from the SV calls and calculated the prevalence of each chr3 translocation types.

We used an independent structural variant calling method LUMPY to validate the translocation events (Layer et al., 2014), selecting the LUMPY Express mode for automated breakpoint detection by using the default parameters. LUMPY Express expected BWA-MEM aligned BAM files as input and automatically parsed sample, library, and read group. Before running LUMPY, we extracted the split and discordant read-pairs based on the whole-genome mapped paired-end sequencing reads of each sample, which were needed by LUMPY. The VCF output file contained the number of supporting reads for each SV event. For those translocation events that were also detected in LUMPY supported by spanning reads, we labeled them ‘Yes’ in the *Validated_by_LUMPY* column (Table S2) indicating that they were validated.

##### Methylation Analysis

Raw data from Illumina’s EPIC methylation array were made available by GCCs as IDAT files from the CPTAC consortium. The methylation analysis was performed using the cross-package workflow “methylationArrayAnalysis” (https://master.bioconductor.org/packages/release/workflows/html/methylationArrayAnalysis.html) available on Bioconductor. In brief, the raw data files (IDAT files) were processed to obtain the methylated (M) and unmethylated (U) signal intensities for each locus. The processing step included an unsupervised functional normalization step that has been previously implemented for Illumina 450K methylation arrays (Fortin et al., 2014). A detection p value was also calculated for each locus, and this p value captured the quality of detection at the locus with respect to negative control background probes included in the array. Loci having common SNPs (with MAF >0.01), as per dbSNP build 132 through 147 via snp132 through snp147 common tracks at the UCSC Browser, were removed from further analysis. Beta values were calculated as M/(M+U), which is equal to the fraction methylated at each locus. Beta values of loci with detection p value > 0.01 were assigned NA in the output file. All loci were annotated with the annotation information file ‘MethylationEPIC_v-1-0_B2.csv’ from the zip archive ‘infinium-methylationepic-v1-0-b2-manifest-file-csv.zip’ (https://www.illumina.com) through the Bioconductor IlluminaHumanMethylationEPICanno.ilm10b2.hg19 package.

##### Classification of Samples with CpG Island Methylator Phenotype (CIMP)

To classify the tumor samples into CpG island methylator phenotypes (CIMP+ and CIMP−), we performed hierarchical clustering of the methylation data using CpG probes previously established to distinguish these epigenetic states in ccRCC (Arai et al., 2012). The parameters used for the hierarchical clustering were “Euclidean” for distance, “complete” for method, and “none” for scale. The resulting two clusters were verified using the histopathological stage data and well-known gene methylation markers (Shenoy et al., 2015). The CIMP+ group was enriched with late-stage tumors and the CIMP+/− differential marker genes were recapitulated based on our classification (Figure S1H; Table S2).

##### Copy-Number Analysis

Copy-number analysis was performed jointly leveraging both whole-genome sequencing (WGS) and whole-exome sequencing data of the tumor and germline DNA, using CNVEX (https://github.com/mctp/cnvex). CNVEX uses whole-genome aligned reads to estimate coverage within fixed genomic intervals and whole-genome and whole-exome variant calls to compute B-allele frequencies (BAFs) at variable positions (we used VarDict germline calls). Coverages were computed in 10kb bins, and the resulting log coverage ratios between tumor and normal samples were adjusted for GC bias using weighted LOESS smoothing across mappable and non-blacklisted genomic intervals within the GC range 0.3-0.7, with a span of 0.5 (the target and configuration files are provided with CNVEX). The adjusted log coverage-ratios (LR) and BAFs were jointly segmented by a custom algorithm based on Circular Binary Segmentation (CBS). Alternative probabilistic algorithms were implemented in CNVEX, including algorithms based on recursive binary segmentation (RBS) (Gey and Lebarbier, 2008), and dynamic programming (Bellman, 1961), as implemented in the R-package jointseg (Pierre-Jean et al., 2015). For the CBS-based algorithm, first LR and mirrored BAF were independently segmented using CBS (parameters alpha = 0.01, trim = 0.025) and all candidate breakpoints collected. The resulting segmentation track was iteratively “pruned” by merging segments that had similar LR, BAFs, and short lengths. For the RBS- and DP-based algorithms, joint-breakpoints were “pruned” using a statistical model selection method (Lebarbier, 2005). For the final set of CNV segments, we chose the CBS-based results as they did not require specifying a prior number of expected segments (K) per chromosome arm, were robust to unequal variances between the LR and BAF tracks, and provided empirically the best fit to the underlying data. The resulting segmented copy-number profiles were then subject to joint inference of tumor purity and ploidy and absolute copy number states, implemented in CNVEX, which is most similar to the mathematical formalism of ABSOLUTE (Carter et al., 2012) and PureCN (Riester et al., 2016). Briefly, the algorithm inputs the observed log-ratios (of 10kb bins) and BAFs of individual SNPs. LRs and BAFs are assigned to their joint segments and their likelihood is determined given a particular purity, ploidy, absolute segment copy number, and number of minor alleles. To identify candidate combinations with a high likelihood, we followed a multi-step optimization procedure that includes grid-search (across purity-ploidy combinations), greedy optimization of absolute copy numbers, and maximum-likelihood inferences of minor allele counts. Following optimization, CNVEX ranks candidate solutions. Because the copy-number inference problem can have multiple equally likely solutions, further biological insights are necessary to choose the most parsimonious result. The solutions have been reviewed by independent analysts following a set of guidelines. Solutions implying whole-genome duplication must be supported by at least one large segment that cannot be explained by a low-ploidy solution, inferred purity must be consistent with the variant-allele-frequencies of somatic mutations, and large homozygous segments are not allowed. Clonal loss or LOH of 3p is very likely in ccRCC, whereas near-haploid and very high ploidy solutions are unlikely.

##### Classification of Samples with Genome instability

To classify the tumor samples into genome instability+ samples and genome stability-samples, we calculated the proportion of genome altered (PGA), which is defined as the length of all segments that have copy number (as inferred by CNVEX) different from C = 2 K = 1; i.e., diploid heterozygous divided by the total length of the genome. The resulting PGA score isabimodal distribution with one peak < 25% and the other peak near 100%. We dichotomized the samples into two genome instability categories using a cutoff of 0.85, which has the local minimal density separating two peaks. The genome instability+ group was enriched with late-stage tumors and the CIMP+/− differential marker genes were recapitulated based on our classification (Figure 1C; Table S2).

#### Transcriptomic Data Processing

Transcriptomic data were analyzed as described previously (Robinson et al., 2017), using the Clinical RNA-seq Pipeline (CRISP) developed at University of Michigan (https://github.com/mcieslik-mctp/crisp-build). Briefly, raw sequencing data were trimmed, merged using BBMap, and aligned to GRCh38 using STAR. The resulting BAM files were analyzed for expression using feature counts against a transcriptomic reference based on Gencode 26. The resulting gene-level counts for protein-coding genes were transformed into FPKMs using edgeR.

#### Proteomic Data Processing

##### Protein database searching and quantification of global and phosphoproteomic data

Raw mass spectrometry files were converted into open mzML format using the msconvert utility of the Proteowizard software suite. MS/MS spectra were searched using the MSFragger database search tool (Kong et al., 2017) against a CPTAC harmonized RefSeq protein sequence database appended with an equal number of decoy sequences. For the analysis of whole proteome data, MS/MS spectra were searched using a precursor-ion mass tolerance of 20 ppm, fragment mass tolerance of 20 ppm, and allowing C12/C13 isotope errors (−1/0/1/2/3). Cysteine carbamidomethylation (+57.0215) and lysine TMT labeling (+229.1629) were specified as fixed modifications, and methionine oxidation (+15.9949), N-terminal protein acetylation (+42.0106), and TMT labeling of peptide N terminus and serine residues were specified as variable modifications. The search was restricted to fully tryptic peptides, allowing up to two missed cleavage sites. For the analysis of phosphopeptide enriched data, the set of variable modifications also included phosphorylation (+79.9663) of serine, threonine, and tyrosine residues.

The search results were further processed using the Philosopher pipeline (https://github.com/Nesvilab/philosopher). Whole proteome and phosphopeptide-enriched datasets were processed separately but using the same pipeline steps, except when noted. First, MSFragger output files (in pepXML format) were processed using PeptideProphet (Keller et al., 2002) (with the high–mass accuracy binning and semi-parametric mixture modeling options) to compute the posterior probability of correct identification for each peptide to spectrum match (PSM). In the case of the phosphopeptide-enriched dataset, PeptideProphet files were additionally processed using PTMProphet (Deutsch et al., 2015) to localize the phosphorylation sites. The resulting pepXML files from PeptideProphet (or PTMProphet) from all 23 TMT 10-plex experiments were then processed together to assemble peptides into proteins (protein inference) and to create a combined file (in protXML format) of high confidence protein groups. Corresponding peptides were assigned to each group. The combined protXML file and the individual PSM lists for each TMT 10-plex were further processed using Philosopher filter command as follows. Each peptide was assigned either as a unique peptide to a particular protein group or assigned as a razor peptide to a single protein group that had the most peptide evidence. The protein groups assembled by ProteinProphet (Nesvizhskii et al., 2003) were filtered to 1% protein-level False Discovery Rate (FDR) using the chosen FDR target-decoy strategy and the best peptide approach (allowing both unique and razor peptides) and applying the picked FDR strategy (Savitski et al., 2015). In each TMT 10-plex, the PSM lists were filtered using astringent, sequential FDR strategy, retaining only those PSMs with PeptideProphet probability of 0.9 or higher (which in these data corresponded to less than 1% PSM-level FDR) and mapped to proteins that also passed the global 1% protein-level FDR filter. For each PSM that passed these filters, MS1 intensity of the corresponding precursor-ion was extracted using the Philosopher label-free quantification module based on the moFF method (Argentini et al., 2016) (using 10 p.p.m mass tolerance and 0.4 min retention time window for extracted ion chromatogram peak tracing). In addition, for all PSMs corresponding to a TMT-labeled peptide, ten TMT reporter ion intensities were extracted from the MS/MS scans (using 0.002 Da window) and the precursor ion purity scores were calculated using the intensity of the sequenced precursor ion and that of other interfering ions observed in MS1 data (within a 0.7 Da isolation window). All supporting information for each PSM, including the accession numbers and names of the protein/gene selected based on the protein inference approach with razor peptide assignment and quantification information (MS1 precursor-ion intensity and the TMT reporter ion intensities) was summarized in the output PSM.tsv files, one file for each TMT 10-plex experiment. The PSM.tsv files were further processed using TMT-Integrator (https://github.com/Nesvilab/TMT-Integrator) to generate summary reports at the gene and protein level and, for phosphopeptide enriched data, also at the peptide and modification site levels. In the quantitation step, TMT-Integrator used as input the PSM tables generated by the Philosopher pipeline as described above and created integrated reports with quantification across all samples at each level. First, PSM from PSM.tsv files were filtered to remove all entries that did not pass at least one of the quality filters, such as PSMs with (a) no TMT label; (b) missing quantification in the Reference sample; (c) precursor-ion purity less than 50%; (d) summed reporter ion intensity (across all ten channels) in the lower 5% percentile of all PSMs in the corresponding PSM.tsv file (2.5% for phosphopeptide enriched data); (e) peptides without phosphorylation (for phosphopeptide enriched data). In the case of redundant PSMs (i.e., multiple PSMs in the same MS run sample corresponding the same peptide ion), only the single PSM with the highest summed TMT intensity was retained for subsequent analysis. Both unique and razor peptides were used for quantification, while PSMs mapping to common external contaminant proteins (that were included in the searched protein sequence database) were excluded. Next, in each TMT 10-plex experiment, for each PSM the intensity in each TMT channel was log2 transformed, and the reference channel intensity (pooled reference sample) was subtracted from that for the other nine channels (samples), thus converting the data into log2-based ratio to the reference scale (referred to as ‘ratios’ below). After the ratio-to-reference conversion, the PSMs were grouped on the basis of a predefined level (gene, protein, and also peptide and site-level for phosphopeptide enriched data; see below for details). At each level, and in each sample, the interquartile range (IQR) algorithm was applied to remove the outliers in the corresponding PSM group. The first quantile (Q1), the third quantile (Q3), and the interquartile range (IQR, i.e., Q3-Q1) of the sample ratios were calculated, and the PSMs with ratios outside of the boundaries of Q1-1.5*IQR and Q3+1.5*IQR were excluded. Then, the median was calculated from the remaining ratios to represent the ratio for each sample, at every level. In the next step, the ratios were normalized using the median absolute deviation (MAD). Briefly, independently at each level of data summarization (gene, protein, peptide, or site), given the p by n table of ratios for entry j in sample i, R_ij_, the median ratio M_i_ = median(R_ij_, j = 1,…,p), and the global median across all n samples, M_0_ = median(M*i*, i = 1,…,n), were calculated. The ratios in each sample were median centered, ${\text{R}}_{\mathrm{ij}}^{\text{C}}={\text{R}}_{\mathrm{ij}}-{\text{M}}_{\text{i}}$. The median absolute deviation of centered values in each sample, ${\mathrm{MAD}}_{\text{i}}=\mathrm{median}\left(\mathrm{abs}\left({\text{R}}_{\mathrm{ij}}^{\text{C}}\right),\text{j}=1\ldots \text{p}\right)$ was calculated along with the global absolute deviation, ${\mathrm{MAD}}_{0}=\mathrm{median}\left({\mathrm{MAD}}_{\text{i}},\text{i}=1,\ldots ,\text{n}\right)$. All ratios were then scaled to derive the final normalized measures: ${\text{R}}_{\mathrm{ij}}^{\text{N}}=\left({\text{R}}_{\mathrm{ij}}^{\text{C}}/{\mathrm{MAD}}_{\text{i}}\right)\times {\mathrm{MAD}}_{0}+{\text{M}}_{0}$. As a final step, the normalized ratios were converted back to the absolute intensity scale using the estimated intensity of each entry (at each level, gene/protein/peptide/site) in the Reference sample. The Reference Intensity of entry I measured in TMT 10-plex k (k = 1,…q), REF_ik_, was estimated using the weighted sum of the MS1 intensities of the top three most intense peptide ions (Ning et al., 2012) quantified for that entry in the TMT 10-plex k. The weighting factor for each PSM was taken as the proportion of the reference channel TMT intensity to the total summed TMT channel intensity. The overall Reference Intensity for entry i was then computed as REF_i_ = Mean(REF_ik_, k = 1,…,q). In doing so, the missing intensity values (i.e., no identified and/or quantified PSMs in a particular TMT 10-plex experiment) were imputed with a global minimum intensity value. The final abundance (intensity) of entry i in sample j (log2 transformed) was computed as ${\text{A}}_{\mathrm{ij}}={\text{R}}_{\mathrm{ij}}^{\text{N}}+\log2\left({\mathrm{REF}}_{\text{i}}\right)$. The ratio and intensity tables described above were calculated separately for each level (gene and protein for whole proteome, and also peptide and site-level for phosphopeptide enriched data). PSMs were grouped as follows. At the gene level, all PSMs were grouped based on the gene symbol of the corresponding protein to which they were assigned as either unique or razor peptides. In the protein tables, identified proteins that mapped to the same gene were kept as separate entries. To generate peptide-level and site-level tables, additional post-processing was applied to generate all non-conflicting phosphosite configurations using a strategy similar to that described in Huang et al. (2017). In doing so, confidently localized sites were defined as sites with PTMProphet localization probability of 0.9 or higher. The same peptide sequences but with different site configurations, i.e., different site localization configurations or peptides with unlocalized sites, were retained as separate entries in the site-level tables. In the peptide-level tables, different site-level configurations were combined into a single peptide-level index, grouping PSMs with all site configurations together if they corresponded to the same peptide sequence. The tutorial describing all steps of the analysis, including specific input parameter files, command-line option, and all software tools necessary to replicate the results are available at https://github.com/Nesvilab.

##### Creation of a Patient-Specific Protein Sequence Database

The proteogenomic database tool pyQUILTS (Ruggles et al., 2016), available at http://quilts.fenyolab.org, was used to incorporate the germline and somatic SNVs and RNA-seq-predicted junctions into a searchable protein database. The human RefSeq protein database (downloaded 2018/06/29) was used as a reference for the hg38 proteome and genome.

##### Variant Peptide Identification and Neoantigen Prediction

NeoFlow (https://github.com/bzhanglab/neoflow) was used for neoantigen prediction. Specifically, Optitype (Szolek et al., 2014) was used to identify human leukocyte antigens (HLA) in the WES data. netMHCpan (Jurtz et al., 2017) was then used to predict HLA peptide binding affinity for somatic mutation-derived variant peptides with a length between 8-11 amino acids. The cutoff of IC_50_ binding affinity was set to 150 nM. HLA peptides with binding affinity higher than 150 nM were removed. Variant identification was also performed at the mRNA and protein levels using RNA-seq data and MS/MS data, respectively. To identify variant peptides, we used a customized protein sequence database approach (Wang et al., 2012). Two different workflows were used. In the first workflow, we derived customized protein sequence databases from matched WES data and performed database searching using the customized databases for individual TMT experiments. We built a customized database for each TMT experiment based on somatic variants from WES data. Customprodbj (https://github.com/bzhanglab/customprodbj) was used to construct customized databases. MS-GF+ was used to identify variant peptides for all global proteome and phosphorylation data. Results from MS-GF+ were filtered with 1% FDR at PSM level. Remaining variant peptides were further filtered using PepQuery (http://www.pepquery.org) (Wen et al., 2019) with the p value cutoff ≤ 0.01. Variant peptide spectra were annotated using PDV (http://www.zhang-lab.org/) (Li et al., 2019). In the second workflow, the RefSeq-based protein database used in the main analysis was trypsin digested *in silico* allowing up to one missed cleavage and treating N-terminal methionine excision as a variable modification to produce two sets of N-terminal peptides (methionine excised and methionine retained). One additional missed cleavage was retained for peptides containing KP and RP amino acids. Isoleucine and leucine occurrences were set to leucine as they are indistinguishable during peptide sequencing. QUILT-derived, patient-specific protein FASTA files corresponding to all samples within a given TMT-plex were combined. From these files, a set of unique peptides was generated from each protein, and any of these peptides that occurred within the RefSeq database were removed. The result of this process was a protein FASTA file, in which every peptide present within the combined personalized QUILT FASTA that was not found within the RefSeq database was retained for searching. The new customized databases were used to search each corresponding dataset against spectra previously classified as having a PeptideProphet score lower than 0.5. The new database searches were carried out using the MSFragger methodology described previously (Kong et al., 2017). Resulting PSMs were filtered, keeping only charge states 2, 3 and 4 and PSMs with delta mass between > −.05 and 2.5. After scoring all identifications with FDRs, the best PSM from each experiment was selected, generating quantitation tables of raw abundance and ratio to common reference. In global and phosphorylation-enriched datasets, post processing was completed to annotate misidentified novel peptides that are contained within UniProt Swiss-Prot (ret. 22 June 2019) and to identify the patients from which each novel peptide was derived. In the phosphorylation-enriched dataset, a column (called isMatch) was added to check whether mutation sites overlap with novel peptide phosphorylation sites. The somatic variant peptides identified by either of the two workflows were used for downstream analysis.

##### Protein database searching and quantification of global data independent acquisition data

Raw mass spectrometry files from DIA (n = 194) and DDA (n = 8) platforms were processed using the DIA-Umpire (Tsou et al., 2015) based pipeline to generate a combined spectral library that integrated DDA and DIA search results. In brief, DIA data were first processed using DIA-Umpire to generate deconvoluted (pseudo-MS/MS) spectra. DDA and pseudo-MS/MS DIA spectra were then searched using the MSFragger (Kong et al., 2017) search engine against the same CPTAC harmonized RefSeq protein sequence database as for TMT data analysis (with an addition of the sequences of iRT peptides). The search was completed using tryptic peptides only, allowing up to two missed cleavages, allowing methionine oxidation and protein N-terminal acetylation as variable modifications, and cysteine carbamylation as a fixed modification. The search results were further processed using the Philosopher pipeline, including PeptideProphet and iProphet (run using the same settings as for TMT data as described above). Retention times of peptide identifications from all DIA and DDA runs were aligned to a single reference DIA run using high quality peptide identifications. ProteinProphet was run using all iProphet pepXML files (i.e., from all DIA and DDA runs) to generate a single combined protXML file, and the protein list was filtered to 1% protein-level FDR. PSMs identified in each individual data subset (DDA or DIA) were then filtered using the Philosopher filter utility to 1% peptide ion-level FDR separately in each subset. Only those PSMs that mapped to proteins in the 1% protein-level FDR filtered combined DDA plus DIA protein list were retained. These filtered PSMs, with aligned retention times, were used to generate two spectral libraries using SpectraST, one for DIA and one for DDA data subsets. The retention times were further transformed to the indexed retention time (iRT) scale using standard peptides spiked into the samples. The consensus MS/MS spectra were generated for each peptide ion, and the spectral libraries were refined to contain only selected peptide fragments using the spectrast2tsv script from the msproteomicstools resource (https://github.com/msproteomicstools), requiring a minimum of three and a maximum of six fragments per spectrum, fragment m/z values between 250 and 2000 Da only, and *b* and *y* ion types only, but allowing neutral losses of water or ammonia. The resulting DIA and DDA spectral libraries were combined. When the same peptide ion was present in both libraries, the DIA spectrum was selected for the combined library. The combined library was then converted to Spectronaut (Biognosys) format for subsequent targeted re-extraction of quantification information. The combined spectrum library was loaded into Spectronaut, and targeted quantification was performed using default settings. For protein quantification, all abundances were calculated as the area under the extracted ion chromatogram (XIC) of all selected fragments that passed filtering. The data were filtered using the global protein-level FDR value of 1%, and proteins were quantified in each individual DIA run using peptides that passed the run-specific peptide ion q-value of 0.01. Protein abundances for select candidates (PECAM1, VEGFA, PKM, HLA-C, C5, HLA-A, PGM1, HLA-B, POSTN, STAT1) were extracted and reported in Table S7. DIA expression matrix of select proteins (ccRCC-DIA_selected_proteins.csv) is available at https://cptac-data-portal.georgetown.edu/cptac/s/S050

##### Preprocessing of ccRCC proteomics data

Global protein and phosphosite abundances from 194 samples were measured in 23 TMT-10plexes in this experiment, which identified 11,355 unique genes in global protein abundance along with 100,730 phosphosites. There were 18.4% data missing in global protein abundance with 7,150 genes completely observed. In the phosphoproteomic analyses, 67.5% of data was missing in phosphosite abundance, with 5,584 phosphosites from 2,443 genes completely observed. Before performing any downstream analysis, we applied batch correction on global and phosphoproteome abundance to remove the technical difference between different TMT 10-plexes. An R tool, ComBat, with tumor/normal status adjustment was applied to remove batch effects (Johnson et al., 2007). To impute missing values, we used DreamAI (https://github.com/WangLab-MSSM/DreamAI), an ensemble algorithm developed during the NCI-CPTAC Dream Proteomics Imputation Challenge (https://www.synapse.org/#!Synapse:syn8228304/wiki/413428). Only those phosphosites and proteins with a missing rate less than 50% were imputed. After imputation, the number of phosphosites deemed valid for downstream analyses was boosted to 26,814 (from 5,571 genes).

##### Sample labeling check of ccRCC dataset

Integrating multiple layers of omics data enhances our understanding of complex molecular mechanisms in biological systems. However, unintended errors in annotations and sample mislabeling often occur when generating and managing large-scale data (Alyass et al., 2015). Since integrative analysis based on erroneous data could lead to inaccurate scientific conclusions, a sample-labeling check is a critical QC step before integration. In this study, we performed a systematic QC procedure to confirm that all annotations in clinical information and sample names were consistent. We checked tissue annotations (tumor or normal), gender annotations (male or female), and sample matching among RNA-seq, proteomics and phosphorylation data.

1. Tissue Annotation. We performed PCA independently in RNA-seq, proteomics and phosphorylation data. As expected, normal and tumor tissues were well separated without exception, suggesting that tissue information is consistent with given annotation.
2. Gender Annotation. Expression of marker genes in X and Y chromosomes can help to infer gender of the samples from which they are analyzed (Staedtler et al., 2013). After combining all normal and tumor RNA-seq samples, male- and female-annotated samples were compared on a t test. Two Y chromosome genes (*RPS4Y1* and *DDX3Y*) showed the strongest separation (t test p values = 6.1 × 10^−140^ and 4.4 × 10^−136^ respectively). When using the expression of these two genes, genders agreed between annotation and expression in all samples. When a similar approach was applied to proteomics and phosphorylation data, the signal was less clear than in RNA-seq data. Hence, it proved difficult to check gender consistency using proteomics and phosphorylation data.
3. Sample Alignment. Although we assessed the accuracy of tissues and gender information, sample mislabeling could still occur by swapping, shifting, or duplicating two samples from the same tissue or genders. Therefore, it is necessary to confirm that complementary samples used in RNA-seq, proteomics, and phosphorylation analyses are from the same individuals. We applied a pairwise alignment procedure developed by us previously (Yoo et al., 2014) using all samples that compared global proteomics and phosphoproteomics, RNA-seq and global proteomics, and RNA-seq and phosphoproteomics. First, the top 500 *cis* genes were identified based on the correlation coefficient for each of proteomics-phosphorylation, RNA-seq-proteomics, and RNA-seq-phosphorylation pair. Then the values of the 500 genes were rank-transformed to obtain a sample-wise similarity score. If a sample matches between two types of data, its sample similarity score should be higher than the score when compared to other cases, which have null distribution with mean 0. Using this approach, we confirmed that all 110 tumor and 84 normal tissues were perfectly matched between global proteomic and phosphoproteomic data. All tumor samples in RNA-seq were well matched with their corresponding global proteomic and phosphoproteomic data except for one normal sample, CPT0012090003, whose self-similarity score did not differ from scores corresponding to other samples (Figure S1A; Table S1). Indeed, the RNA expression of this sample did not match its proteomic profile (both global and phosphoprotein abundance). While this error could arise from either RNA-seq orglobal-proteomic/phosphorylation data since global proteomic and phosphorylation data for this sample were well aligned. This sample was removed from all further downstream analysis as the error-source remained unresolved.

#### CNV Integrated Analysis

##### Genomic determination of 103 ccRCC versus 7 non-ccRCC samples from 110 pathologically defined ccRCC tumors

Outlier samples identified PCA of by RNA-seq data using Omics Explorer (Qlucore, Lund, Sweden) also exhibited low expression of ccRCC biomarkers such as *ANGPTL4, CA9*, and *NDUFA4L2*, among others and were subject to further critical evaluation for genomic aberrations (copy number variations (CNVs) and mutations) (Figures S1B–S1D). Samples C3N-00492 and C3N-00175 showed one copy loss of chromosomes 1, 2, 6, 10, 13 and 17 along with *TP53* mutations and contained high expression of several biomarkers (such as FOXI1, RHCG) (Lindgren et al., 2017) that are characteristic of chromophobe RCC (chRCC). In addition, samples C3N-00832 and C3N-00313 contained *PTEN* mutations, and the latter also showed outlier expression of the papillary RCC biomarker *VSTM2A* (Wang et al., 2018a), along with gain of chr7, and *PIK3R1* mutation and were thus categorized as likely papillary RCCs. Sample C3L-00359 contained bi-allelic loss of *TSC1* along with histologic features of eosinophilic solid cystic RCC (ESC-RCC) (Mehra et al., 2018), while C3N-01180 had a *SFPQ-TFE3* gene fusion, a hallmark of translocation RCC. Finally, C3N-00435 contained 3p loss with a *PIK3CA* hotspot mutation. These seven samples were therefore annotated as non-ccRCC samples and excluded from the ccRCC cohort in all downstream analyses (Table S1).

##### Detection of Chromosomal Arm-Level Aberration

GISTIC2 (Mermel et al., 2011) was used to process the segment-level CNV log ratios and define chromosome arm-level gain/loss events for 3p, 5q, 7p, 9p, and 14q, using the default threshold setting (i.e., -ta and -td were both set to 0.1). From the GISTC2 arm-level outputs, we defined the positive and negative values as arm gain and loss events, respectively. Since GISTIC2 by design ignores arm-level CNVs, we next identified arm-level recurrence of gains/losses using an alternate approach. For each chromosome arm, we calculated the average (weighted) maximum-likelihood copy number (clonal orsubclonal) relative to 2 (diploid). Similarly, for each chromosome arm, we calculated the proportion of the arm that shows LOH, including copy-neutral LOH. Following thresholding, (for CN gain (> 0.5) or loss (< −0.5)) these two proportions were used to estimate population-level recurrence of arm-level losses/gains and LOH, respectively. For chromosome 3p, only the loss (< −0.5) was investigated for LOH (defined as > 0.5). The detailed annotation of all chromosome arm events is provided (Table S2).

##### Heatmap Web Server

We developed an online application that allows researchers to query the dataset for genes of interest, rendering a downloadable table and corresponding heatmap visualization of the select data. The underlying data consist of quantitative information on copy number alteration, mutation, methylation, RNA-seq gene expression, protein expression, and phosphosite expression for 22,867 genes across 110 samples. Data tracks for each gene are labeled as: “Mut”-mutation data (“Yes” is any types of mutation, “No” is no mutation), “Methy”-beta value of CpG island in the promoter region of the gene (standardized), “CNV (lr)”-the log ratio of copy number variation, “CNV (baf)”-the b-allele frequency of the copy number variation (standardized), “mRNA”-gene expression levels (standardized), “Protein”-gene-level protein abundance (standardized), and “Phospho”-gene-level phosphoprotein abundance (standardized). Genomic and clinical annotation data are displayed on the top and bottom of the visualization. Tracks on the top include genomically confirmed ccRCC and non-ccRCC, 3p copy number variation, and an immune grouping for each sample. Bottom-placed tracks visualize CNV data for chromosomes 5q, 7p, 9p, and 14q, t(3;2) and t(3;5) chromosome translocations, CIMP status, and genomic instability status, as well as grade, stage, and gender information. The application can be accessed at http://ccrcc.cptac-data-view.org. This is an entirely web-based application, and users do not need to download any software outside of a web browser to visualize and access the data. Users begin by entering official symbols for up to thirty genes into a text field. For convenience, the input gene list may be separated by tabs, commas, semicolons, single spaces, or line breaks. All gene symbols found in the underlying dataset will be used to generate an Excel file (.xls) and corresponding heatmap visualization. The raw data and graphic visualization (.png) can be downloaded to a local computer for further exploration or for use in publication. The application can also be used for interactive visual exploration of the data. Users may click any data point on the interactive heatmap to view the underlying values, including the sample identifier, data type, and value. By clicking a link next to the sample identifier, users can view direct links to the histological images corresponding to the sample, organized by tissue location. When available, the user can click gene symbols on the heatmap to view summary descriptions from the NCBI. Users can sort the entire heatmap by a single data track, in ascending or descending order. The sorted visualization can then be downloaded with a button. This functionality enables users to explore dynamically the relationships and patterns among different tracks.

##### Downstream Analysis of Chromosome Arm 3p translocation

This cohort featured frequent chromosome 3p arm translocation (e.g., to chromosome 5q and results in 3p loss and 5q gain) (Figure 1B). An association analysis of chromosome 3p arm translocation events was performed with each of the 9,190 protein abundances (with missing rate < 50%) across 110 ccRCC tumor samples to identify the proteomic functional impacts of the translocation events. Specifically, three types of translocation categories, Chr3-Chr2, Chr3-Chr5, and Chr3-other, were simultaneously considered in the regression models, and an ANOVA test was applied to assess whether any translocation was associated with the protein abundances in the tumors. Age, gender, ischemic time, OCT status and tumor purity were adjusted as covariates. The most significant protein was SETD2 (p = 8E - 6; FDR < 0.05). Post-ANOVA assessment of each event indicated that Chr3-Chr2 translocation was significantly associated with decreased abundance in SETD protein abundance (Figure S1F), which is consistent with the high mutation rate observed in Chr3-Chr2 group (Figure S1E).

##### *iProFun-Based* Cis *Association Analysis*

The integrative analysis tool, iProFun (Song et al., 2019), was used to identify functional molecular quantitative traits perturbed by DNA-level variations (https://github.com/WangLab-MSSM/iProFun). Compared to analyzing each molecular trait separately, iProFun models multi-omic data jointly, thereby enhancing the power for detecting significant *cis*-associations shared across different omics data types and achieves better accuracy in inferring *cis*-associations unique to certain types of molecular traits. Specifically, we considered three functional molecular quantitative traits (mRNA expression levels, global protein abundances, and phosphopeptide abundances) for their associations with four DNA-level variations (copy number alterations measured by log ratios, copy number alterations measured by b-allele frequency, DNA methylations and somatic mutations).

##### Data and preprocessing:

We analyzed data from 110 tumors in the CPTAC cohort. mRNA expression levels measured with RNA-seq were available for 19,293 genes (https://cptac-data-portal.georgetown.edu/cptac/s/S050; RNA_rpkm_tumor_normal.tsv), while global protein abundance measurements were available for 11,355 genes (https://cptac-data-portal.georgetown.edu/cptac/s/S050; CPTAC3_CCRCC_Whole_ abundance_gene_protNorm = 2_CB.tsv) and the phosphopeptide abundances were available for 42,893 peptides (https://cptac-data-portal.georgetown.edu/cptac/s/S050; CPTAC3_CCRCC_Phospho_abundance_phosphopeptide_protNorm%3D2_CB_ 1211.tsv) from 8,502 genes. The log ratios and b-allele frequencies of copy number alterations were obtained from WGS and WES data using a segmentation method for 19,285 and 19,293 genes, respectively. The DNA methylation levels (beta values) averaging the CpG islands located in the upstream and nearby transition starting site (TSS) regions, including 5UTR, first exon, and upstream TSS were available for 15,885 genes. Somatic mutations were called using WES (see SNV calling section above). All data types were preprocessed to eliminate potential issues such as batch effects, missing data, and major unmeasured confounding effects. All seven types of data were filtered with a missing rate > 50%. mRNA expression levels and global protein and phosphoprotein abundances were also normalized to a standard normal distribution. Somatic mutations with > 5% frequency were considered in iProFun for their functional consequences on molecular quantitative traits (QTs). To account for potential confounding factors, we considered age, gender and tumor purity. Tumor purity was determined from RNA-seq data using ESTIMATE (Yoshihara et al., 2013).

##### iProFun procedure:

The iProFun procedure was applied to 4,009 genes measured across all six data types (mRNA, global protein, phosphoprotein, CNA – lr, CNA – baf, DNA methylation) for their *cis* regulatory patterns in tumors. Thirteen genes with frequent somatic mutations (mutation rate > 5%) were also considered for their effects on *cis* molecular QTs. Specifically, for the remaining 3986 genes, we considered the following three regressions:

mRNA∼CNV(lr)+CNV(baf)+methy+covariates,

global∼CNV(lr)+CNV(baf)+methy+covariates, and

phosphor∼CNV(lr)+CNV(baf)+methy+covariates.

For the thirteen genes with frequent somatic mutations we considered the following three regressions:

mRNA∼CNV(lr)+CNV(baf)+methy+mutation+covariates,

global∼CNV(lr)+CNV(baf)+methy+mutation+covariates, and

phosphor∼CNV(lr)+CNV(baf)+methy+mutation+covariates,

The association summary statistics of CNV (lr), CNV (baf), and methy from two sets of regression frameworks were combined and applied to iProFun to call the posterior probability of belonging to each of the eight possible configurations (“None,” “mRNA only,” “global only,” “phosphor only,” “mRNA & global,” “mRNA & phosphor,” “global & phosphor,” and “all three”) (Figure S2A), to estimate the variation of molecular QTs explained by each DNA variations (R2), and to determine significance associations.

Table S3 and Figure S2B present the significant genes that pass three criteria: (1) satisfying the biologic filtering procedure, (2) posterior probabilities > 75%, and (3) empirical false discovery rate (eFDR) < 5%. Specifically, we posed the biological filtering criterion for CNV and DNA methylations. Only CNV (lr) that were positively associated with all the types of molecular QTs, DNA methylations that were negatively associated with all types of molecular QTs, and CNVs (baf) with associations of consistent direction (either positive or negative) with all types of molecular QTs were considered for significance calling. A significance was then called only if the posterior probability > 75% of a predictor being associated with a molecular QT was greater than 75%, by summing over all configurations that are consistent with the association of interest. For example, the posterior probability of a DNA methylation being associated with mRNA expression levels was obtained by summing up the posterior probabilities in the following four association patterns – “mRNA only,” “mRNA & global,” “mRNA & phosphor,” and “all three,” all of which were consistent with the association of DNA methylation with mRNA expression. Lastly, we calculated empirical FDR (eFDR) via 100 permutations per molecular QTs by shuffling the label of the molecular QTs and requesting eFDR < 5% by selecting a minimal cutoff value of alpha such that 75% < alpha < 100%. The eFDR is calculated by:

eFDR=(Averaged number of genes with posterior probabilities>alpha in permuted data)/×(Averaged number of genes with posterior probabilities>alpha in original data)

Among all the genes whose phosphoproteins were significantly associated with tumor versus normal and with tumor grade, CNV, and methylation with cascade effects, i.e., demonstration of significant association with all of the three traits (mRNA levels, protein and phosphopeptide abundances) were plotted in Figure 2A. Table S3 catalogs R2 range (0,1) by providing the percentage of adjusted variances explained by each type of DNA alterations. These R2 were obtained by contrasting multiple regression R2 values between the full model and models without the predictor of interest. For example, to calculate R2 of DNA methylation of a gene with a low mutation rate on its *cis* mRNA, the R2_full value is generated from model mRNA ~CNV (lr) + CNV (baf) + methy + covariates, whereas the R2_reduced value is from model mRNA ~CNV (lr) + CNV (baf) + methy + covariates. The difference, R2_diff is calculated as R2_full - R2_reduced and represents R2 of mRNA explained by DNA methylation in this gene. For the additional thirteen somatic mutations, posterior probabilities could not be called robustly using iProFun, as alternative densities used in iProFun cannot be inferred accurately using only thirteen observations. We only required eFDR < 5% and consistent direction in association with all types of molecular QTs to call significance. The R2 can be obtained in the same way as in other DNA variations. Table S3 presents the iProFun results based on measured mRNA and protein, without considering phosphoprotein. This exercise begins with a large number of genes that are measured across mRNA and protein.

##### Trans Association Analysis

We analyzed ten genomic features to understand their *cis* and *trans* associations with multi-omic molecular QTs in 110 cases in this cohort (Figures 2B and S2D). Specifically, we considered multiple linear regression to evaluate the association between each pair of genomic feature and molecular trait adjusting for potential confounding factors. The mRNA expression and global protein abundances were considered at gene-level, and the phosphopeptide data were considered at the peptide level. In ccRCC, a total of 19,293 mRNA expression levels, 11,355 global protein abundances and 42,893 peptide-level phosphoprotein abundances were quantified, respectively. We required a missing rate < 50% for consideration in association analyses, with 17,461 mRNA expression levels, 9,190 protein abundances and 21,245 phosphopeptide abundances being analyzed. All outcomes were normalized to match to a standard normal distribution before entering the regression models. We considered five chromosome arm-level genomic features (3p, 5q, 7p, 9p and 14q) that are most prevalent in the genome, one global methylation feature (CpG Island Methylator Phenotype (CIMP)), and five key mutations (*VHL, PBRM1, BAP1, SETD2, KDM5C*) for ccRCC (Table S3). For chromosome arms 3p and 14q, we compared copy loss with copy neutral. For chromosome arm 5q and 7p, we compared amplification with neutral. In 7p, we excluded four samples with loss from the analysis. CIMP was treated as a categorical variable; CIMP+ and CIMP− were compared in regression. For five mutations, we grouped all mutation types for power purposes and compared mutation versus no mutation in the analysis. We adjusted for potential confounding factors that could affect the association between *trans* genomic features and molecular traits in the analyses, including age, gender, OCT embedding, ischemic time, and tumor purity. Ischemic time was calculated as minutes from the initial clamping to collection and minutes from collection to freezing, if a clamp was used in the surgery. If no clamp was used in the surgery, the ischemic time was defined as minutes from collection to freezing. We considered eFDR to call for significance to account for complex unknown gene/gene correlations. Specifically, we first calculated t statistics for the association between a genomic feature and all genes/peptides of a given data type (e.g., mRNA) via multiple regression, thus adjusting for confounding factors. We then permuted our sample 100 times by shuffling the sample label of the outcomes to re-calculate the t statistics. For a pre-specified t statistics cut-off value, T, a gene was considered positive if it’s absolute t statistics were greater than T. Empirical FDR was calculated as noted previously for a pre-specified T value. The smallest T values that allow an averaged empirical FDR < 10% were used as the final cutoffs, and all genomic feature and molecular trait pairs with absolute t statistics greater than the cutoff were considered significant associations. The significant *trans* associations of the selected key features (3ploss, 5q amplification, 7p amplification, 9ploss, 14q loss, CIMP, *VHL, PBRM1, BAP1, SETD2, KDM5C*) Figures 2B and S2D) were binarized to +1 (positive) and −1 (negative) according to the calculated association values above and visualized in Figures 2B and S2D using OmicsOne (Hu et al., 2019), a toolkit for data visualization and analysis of multi-omic data (https://github.com/ HuiZhangLab-JHU/OmicsOne). The cumulative density of the positive and negative associations on each individual chromosome was counted to illustrate the propagation heterogeneity of *trans* associations on different chromosomal locations. For each one of the eleven genomic events, pathway analyses were considered based on their association summary statistics with 9,190 genes that were observed in more than 50% of the 110 clinical samples. Specifically, a quantity T = − sign(beta) log(p value) was considered for the association between each pair of genomic event and protein abundance. The T value will be extremely high if the genomic event is significantly upregulated in the protein abundance, extreme negative values if the genomic event is significantly downregulated in the protein abundance, and values close to zero if the genomic event is not associated with the protein abundance. For each genomic event and each pathway under consideration, we tested if T_in = T_out using a two-sided Wilcoxon rank-score test, where T_in are the T values for all proteins inside of the pathway and T_out are the T values for all proteins outside of the pathway. Databases from Hallmark (MsigDB Collections), KEGG, and Reactome were combined for pathway analysis (Joshi-Tope et al., 2005; Liberzon et al., 2011; Ogata et al., 1999). The significance threshold was set as FDR < 0.05 to identify pathways that were most significantly associated with the genomic features. The direction of regulation (up versus down) was given by test statistics, with T_in > T_out indicating upregulation and T_in < T_out indicating downregulation.

##### Correlation of mRNA and Protein Expression

A total of 7,026 genes with complete mRNA (https://cptac-data-portal.georgetown.edu/cptac/s/S050; RNA_rpkm_tumor_ normal.tsv) and protein (https://cptac-data-portal.georgetown.edu/cptac/s/S050; CPTAC3_CCRCC_Whole_abundance_gene_ protNorm = 2_CB.tsv) data across all 103 ccRCCs and 72 NAT samples were used to measure gene-wise (Figure 3A) and sample-wise (Figures 3B and S3A) mRNA and protein correlations. Spearman correlation was calculated for each mRNA-protein pair across tumors and NATs separately and for each individual sample across 7,026 genes (Table S4). GSEA was used for gene-wise investigation on the correlation-ranked list of genes to determine if functional groups (pathways or complexes) were non-randomly distributed in terms of mRNA-protein correlation (Subramanian et al., 2005). Functional classes were obtained from the MSIGDB (https://www.broadinstitute.org/gsea/msigdb/index.jsp) and were chosen from the most significant non-redundant functions that were biologically informative. The individual proteins associated with pathways highlighted in the text as being significantly differentially present in more- or less-correlated protein-mRNA pairs are presented. To determine which pathway or functional activity may contribute to the sample-wise mRNA-protein correlation, we calculated the Spearman correlation between index and protein expressions across 103 tumor samples for 7,026 proteins using the correlation as index for each sample. Similarly, functional pathways were enriched and selected in those high correlations by GSEA enrichment analysis using the correlation-ranked list of proteins (Figures 3B, 3C, S3B, and S3C). In addition, we assessed the association between DNA aberrations such as CNV data and mutations and clinical phenotypes such as tumor grade. A univariate linear model was utilized in which the pairwise correlation was modeled as a linear function of DNA aberrations and clinical phenotypes (Table S4). To assess whether the association between pairwise correlation and those covariates was induced by their shared dependence on tumor purity, tumor purity was included in the linear model as a covariate (Table S4). All figures were visualized using OmicsX (Pan et al., 2019), a webserver for data analysis and visualization (http://bioinfo.wilmer.jhu.edu/OmicsX/).

#### Differential Abundance of mRNA, Protein, and Phosphoproteome Measurements

##### Principal Components Analysis

We performed PCA on 103 tumor samples and 80 normal adjacent (NAT) samples to illustrate the global proteomic difference between tumor and NAT samples (Figure 4A). The PCA function under the scikit-learn R package (Pedregosa et al., 2011) was implemented for unsupervised clustering analysis with the parameter ‘n_components = 2’ on the expression matrix of global proteomic data containing 7000+ proteins (features). The 95% confidence coverage was represented by a colored ellipse for each group, which was calculated based on the mean and covariance of points in each specific group (tumor and NAT).

##### Global Heatmap

Two-way hierarchical clustering was applied to the global proteomic data on samples and proteins to identify the global differential protein expression and protein co-expression patterns (Figure S4A). Each gene expression value in the global proteomic expression matrix was transformed to a z-score across all samples. For the sample-wise and protein-wise clustering, distance was set as “Euclidean” distance, and the weight method was “complete.” The z-score-transformed matrix was clustered using R package: pheatmap (version 1.0.10).

##### Tumor versus Normal Differential Proteomic Analysis

TMT-based global proteomic data were used to perform differential proteome analysis between tumor and normal samples (Figures 4B, S4B, and S4C; Table S5). A Wilcoxon rank sum test was performed to determine differential abundance of proteins between tumor and normal samples. The significantly differentially expressed gene lists (fold change > = 2 and FDR < 0.05) were used to perform overrepresentation enrichment analysis (ORA) implemented in WebGestaltR (Wang et al., 2017), in which the parameters were set to use 9190 background genes and the combined KEGG/HALLMARK/Reactome database as described above.

##### Accounting for Tissue Purity in Differential Analysis based on Proteomic Data

NAT and tumor tissues represent mixtures of epithelial, stromal and immune cells. TSNet (Petralia et al., 2018) was used to account for this tumor heterogeneity and identify proteins that are differentially expressed between pure-tumor and pure-NAT cells. TSNet models the global abundance of each protein as a mixture of pure component and a component that captures the immune and stromal infiltration in a particular tissue. This algorithm estimated a mean parameter for pure component and immune/stromal infiltrated component for each protein. TSNet was applied to tumor (T) and NAT (N) samples separately by estimating the following two models:

$$X_{T,i.j}=\pi_{T,i}y_{T,i,j}+\left(1-\pi_{T,i}\right)Z_{T,i,j}$$

$$X_{N,i,j}=\pi_{N,i}y_{N,i,j}+\left(1-\pi_{N,i}\right)Z_{N,i,j}$$

with $y_{T,i,j}∼Normal\left(\mu_{T,j}^{Y},\sigma_{T,j}^{Y}\right),Z_{T,i,j}∼Normal\left(\mu_{T,j}^{Z},\sigma_{T,j}^{Z}\right),y_{N,i,j}∼Normal\left(\mu_{N,j}^{Y},\sigma_{N,j}^{Y}\right),Z_{N,i,j}∼Normal\left(\mu_{N,j}^{Z},\sigma_{N,j}^{Z}\right),X_{t,i,j}$ being the observed global abundance of sample *i* and gene *j* for tissue $t\in \left\{T,N\right\},\pi_{t,j}$ being the tissue purity for sample *i* and tissue *t* and $\left(y_{t,i,j},z_{t,i,j}\right)$ being latent variables corresponding to the protein abundance that would be observed in pure-tissue (i.e., $y_{t,i,j}$) and immune/stromal cells (i.e., $z_{t,i,j}$*).* Given the consistency of purity values estimated by TSNet and ESTIMATE (Yoshihara et al., 2013) for this analysis, purity was inferred via ESTIMATE and considered as fixed (Table S7). Before implementing TSNet, each protein was z-score normalized across NAT and tumor samples. Once that the signal was deconvolved into pure-tissue and immune/stromal components, we identified proteins that were differentially expressed in pure tumor component compared to pure NAT component. Mathematically, this was achieved by assessing the significance of the difference between the means $\mu_{N,j}^{Y}\;\mathrm{and}\;\mu_{T,i}^{Y}$ for each protein *j.* Significance was assessed via permutation. Specifically, TSNet was implemented under permuted data, where the labels of NAT and tumor samples were randomly shuffled. For this analysis, 200 permutations were considered. Using the null density of mean difference derived based on permutated data, we assessed the significance of the up/downregulation in tumor compared to NAT. In particular, at a specific FDR cut-off of 10%, true associations were computed using the strategy illustrated by Tusher et al. (2001). To be consistent with the differential analysis based on un-deconvolved data, among the selected proteins at FDR 10%, only mean differences with a fold change greater than two were considered significant. Reported pathways are listed in Table S5.

##### Accounting for Anatomic Region of NAT

Using published gene signatures (Lindgren et al., 2017), NAT samples were allocated to different anatomic regions, including cortex, medulla, corticomedulla (Tal), inflammatory (Infla), and endothelial and/or smooth muscle cells or fibroblasts (SMC). Gene expression of all the genes (TPM) was z-scored normalized across samples and then averaged across genes mapping to each anatomic region subtype. Each sample was then allocated to the anatomic group with the highest score. A one-sided Wilcoxon test was performed to compare ccRCC versus NAT samples allocated to the cortex anatomic region. P values were adjusted for multiple comparisons using a Benjamini Hochberg adjustment. Only proteins with an adjusted p value less than 5% and fold change greater than two were considered significant. Reported pathways are listed in Table S5.

##### Metabolic Reprogramming in ccRCC

A Wilcoxon rank sum test was performed to explore tumor-normal differential analysis for RNA and protein at the gene level (Figure 4C). Genes associated with glycolysis, the TCA cycle (Krebs Cycle), and oxidative phosphorylation (electron transport chain) were focused for metabolic reprogramming. In tumor samples, metabolic reprogramming-associated genes were selected, and z-score transformation was performed. For genes detected at both the mRNA and protein levels, t tests were performed to compare the gene/protein expression between tumor and NAT, separately. The log2 fold changes were used to measure the expression difference and significance and the concordance between mRNA level and protein measurements. Finally, the difference of log2 fold change between mRNA and protein were input into GSEA (Table S4) to investigate enriched pathways. The enriched concepts indicates the discordance between mRNA and protein for tumor/normal difference (Figures 4D and S4C). Figures 4D and S4C were visualized using OmicsX (http://bioinfo.wilmer.jhu.edu/OmicsX/) (Pan et al., 2019).

#### Phosphoproteomic Analysis

##### Phosphopeptide Analysis – Kinase and Substrate Regulation

To discover the phosphorylation events that were relevant to ccRCC, we utilized phosphopeptide-level data to examine the overall relationship between phospho-substrates and their associated kinases (Figures 5A and S5A). The kinase-substrate association was first extracted from PhosphoSitePlus (Hornbeck et al., 2015) to eliminate phosphopeptides (https://cptac-data-portal.georgetown.edu/cptac/s/S050; CPTAC3_CCRCC_Phospho_abundance_phosphopeptide_protNorm = 2_CB_imputed_1211.tsv) containing phosphosites (https://cptac-data-portal.georgetown.edu/cptac/s/S050; CPTAC3_CCRCC_Phospho_abundance_phosphosite_ protNorm = 2_CB_imputed.tsv) that were not reported as well as those without associated kinases identified in our global dataset (https://cptac-data-portal.georgetown.edu/cptac/s/S050; CPTAC3_CCRCC_Whole_abundance_gene_protNorm = 2_CB.tsv). Next, we inspected any substantial differences among 80 tumor/NAT pairs, especially those that showed higher changes in tumors, by calculating the fold change (log2 scale). We then ranked each tumor (> 1.5 fold increase) among different kinase substrates to obtain the highest ranked phospho-substrate events in the majority of tumors (Table S6). Finally, we identified nine phospho-substrate events of eight kinases with inhibitors that are either FDA-approved or in clinical trials (Carles et al., 2018; Ferguson and Gray, 2018). We also calculated the fold change of the selected phospho-substrates and kinases in other omics data (e.g., mRNA) to examine any difference in expression level among multiple omics data types (Figures S5A–S5C). In addition, we compared the phosphopeptide expression between low-grade tumors (Grades 1 and 2) and high-grade tumors (Grades 3 and 4) as well as between low-stage tumors (Stages 1 and 2) and high-stage tumors (Stages 3 and 4). A p value < 0.05 (Mann–Whitney U test) was considered as significant (Figure S5B). Data were visualized using Omic-Sig (https://github.com/hzhangjhu/Omic-Sig) (Lih et al., 2019).

##### Phosphoproteomic Co-expression Network Inference

Network inference was utilized to characterize co-expression patterns among phosphopeptides in ccRCC. Due to the high dimensionality of phosphorylation data, which contained approximately 20,000 different peptides, phosphopeptides were first clustered into three groups, and then co-expression networks were estimated for each group, separately (Figures 5C, 5D, and S5D–S5G). Specifically, we first summarized multiple phosphopeptides mapping to the same protein using their leading principal component, which was derived based on 103 ccRCC tumor samples and 80 NAT samples. k-means clustering was then implemented on the gene-level matrix to cluster proteins into three groups. This procedure resulted in one group containing 1,842 genes mapping to 6,182 phosphopeptides, a second group containing 1,963 genes mapping to 6,976 phosphopeptides, and a third group containing 2,047 genes mapping to 7,818 phosphopeptides. For each group of genes, a co-expression network was estimated based on phospho-peptide level data through a random-forest-based algorithm (Petralia et al., 2015). In particular, co-expression networks were estimated using missing data-imputed peptide-level phosphorylation data (https://cptac-data-portal.georgetown.edu/cptac/s/S050; CPTAC3_CCRCC_Phospho_abundance_phosphopeptide_protNorm = 2_CB_imputed_1211.tsv). Let *p* represent the total number of phosphopeptides measured for *n* samples. $X_{i,j}^{S}$ represents the abundance of the *j*-th peptide mapping to the *s*-th protein for the *i*-th sample. $X_{i,j}^{S}$ was modeled as a function of other protein phosphopeptides, i.e., ${\left\{X_{i,j}^{k}\right\}}_{k\neq s}$, via random forest. To facilitate the comparison with networks inferred based on RNA-seq and global proteomic data, the network was obtained at the gene-level with nodes corresponding to genes. Basically, an edge between two genes was drawn if at least some peptides mapping to the two genes were found to be associated. This was achieved by using an extension of the random forest algorithm (https://github.com/WangLab-MSSM/ptmJRF) (Petralia et al., 2016). Basically, for each protein *j*, the abundance of each phosphopeptide mapping to that protein was modeled as a function of other proteins’ phosphopeptides via random forest. At each node in the random forest tree, *M* proteins were randomly sampled and proposed as candidates for the splitting rule. Then, across all phosphopeptides mapping to the *M* proteins, the phosphopeptide resulting in the lowest node impurity was utilized for the splitting rule. A separate collection of T trees was estimated for each phosphopeptide mapping to protein j. Based on each ensemble tree, an importance score capturing the association between protein *k* and *j* was derived (Petralia et al., 2015). The final weight assigned to the relationship (k→j) was derived by taking the maximum of the importance scores across different tree ensembles. This procedure was repeated for each protein *j.* The final importance score assigned to the edge (k−j) was derived as the average between the importance score corresponding to (k→j) and the importance score corresponding to (j→k) (Petralia et al., 2016). To derive the final unweighted networks, a proper cut-off value was chosen via permutation techniques (Petralia et al., 2016). Specifically, 40 permutations and a FDR cut-off of 5% were considered to derive the final network. Table S6 contains the list of network edges of genes obtained at 5% FDR cut-off.

##### Phosphoproteomics Co-expression Network Modules

Network-modules were derived using Glay (Su et al., 2010), a community clustering algorithm available through Cytoscape (Morris et al., 2011; Shannon et al., 2003). Thirty network-modules containing at least twenty genes were identified (Table S6). Considering the list of genes mapping to each network module, pathway enrichment analysis was performed to identify biological pathways overrepresented in each network module via Fisher’s exact test. For this analysis, pathways from the KEGG (Kanehisa and Goto, 2000) and Reactome (Joshi-Tope et al., 2005) database were considered. Table S6 shows the list of enriched pathways for each network-module. A one-sided t test was used to identify 18 network modules whose nodes were more correlated under the Phospho-Tumor network than under other data types (Table S6). To visualize the network modules (Figures S5D and S5E) the software iCAVE (Kalayci and Gümüş, 2018; Liluashvili et al., 2017) and Cytoscape were utilized.

##### Interactive Network Exploration Portal

We developed a web portal that allows researchers to interactively explore tumor phosphoproteomics co-expression network and its modules (http://ccrcc.cptac-network-view.org). This web-based application does not require users to download any software outside of a web browser to access and explore the data. The main page features a panel on the left that enables 3D viewing and exploration of the tumor phosphoproteomics co-expression network. Nodes are colored accordingly with associated modules that are listed to the right of the viewing panel. Users can search for a certain gene within the network by entering the HUGO symbol in the search box provided and clicking the Search button. If found, the gene is highlighted in the 3D panel in red, and the associated information about the gene is provided under the search box. Gene information includes the list of genes that are directly connected to the queried gene and also association of the gene with clinical variables (FDR value and p value for grade, gender, age, and stage). Users can click on the name of a module to view it in detail. A separate page opens and displays module-specific network and associated details. Module-specific pages provide the 3D network view and exploration panel at the center of the page. Users can interactively explore the network in 3D using this panel. Node sizes are proportional to the number of connections. Hovering over a node displays the gene name and highlights it in red. Clicking on a node will highlight the edges connected to the node in red and also display associated information on the right side. Gene-associated information includes the list of genes that are directly connected to the gene, peptides associated with the gene, and the association of the gene with clinical variables (FDR value and p value for grade, gender, age, and stage). Above the network-view panel, another panel can perform phenotype-related search operations. Users can select the phenotype of interest (grade, gender, age, or stage), enter an FDR cutoff value, and click the Submit button. Genes that satisfy the search metrics are listed in the text box and are also highlighted in the network panel in red. Users can click the Reset button to return to the original network. On the left side of the network view panel, an interactive table showing the list of enriched pathways (if any) is provided. Users can click on a pathway name, and the genes within the pathway will be listed in the text box as well as highlighted in the network panel in red. Users can click the Reset button to return to the original network. The interactive network exploration portal utilizes multiple client-side Javascript libraries (e.g., three.js, D3.js, JQuery) to facilitate visualization and user interaction with large volumes of data in real time. For 3D visualizations that are displayed within the interface, we incorporated 3D layouts from iCAVE (Kalayci and Gümüş, 2018; Liluashvili et al., 2017) and customized them to serve the specific needs of our tool. We also incorporated other utility libraries (e.g., dataTables.js) for data manipulation and interaction. For web interface styling, we relied primarily relied on Bootstrap v3.3.7, integrated with our custom CSS elements. Since our implementation utilizes only standard libraries and does not necessitate any external plug-ins, the portal runs on all modern web browsers.

#### ccRCC Inter-Tumor Proteome Heterogeneity

##### Proteomic Subtyping

We investigated the molecular subtyping of all tumor samples based mainly on global proteomic expression to identify the associations between the multi-omics expression and clinical phenotypes, such as tumor stage and grade (Figure 7B; Table S5). The 3,567 (50%) most variable global proteins without missing values were analyzed by CancerSubtypes (Xu et al., 2017) for consensus clustering (Monti et al., 2003) of tumor subtypes. Specifically, 80% of the original sample pool was randomly subsampled without replacement and partitioned into three major clusters using hierarchical clustering, which was repeated 500 times (Wilkerson and Hayes, 2010). The expression values were transformed into Z scores at the gene level using the built-in standardization function of R. The consensus-clustered samples were ordered according to the calculated distance and associated with stage and grade, four key mutations (*VHL, PBRM1, SETD2*, and *BAP1*), and the consensus clustering results from other omics data, including immune subtypes. Proteins were grouped into three clusters using hierarchical clustering. The overrepresentation analysis (ORA) was performed on the gene list of each protein cluster using WebGestaltR (the R Version of WebGestalt). The parameters were set as described above. The significance threshold was set as FDR < 0.05 to identify and annotate the pathways most-associated with each protein cluster.

##### Multivariate Analysis

To investigate the possible associations between genome-/proteome-wide data and clinical features, we utilized multiple omics data including RNA-seq, proteome, and phosphoproteome of tumor and NAT samples to conduct the association analysis (Figures 7A, S7A, and S7B; Table S5). We also incorporated CNV and DNA methylation data from tumors only in the analysis. Our set of clinical features consists of tumor characteristics (e.g., stage, grade, margin status, left-right kidney laterality, presence of necrosis), patient properties (e.g., age, gender, BMI, country of origin), lifestyle, medical history (e.g., smoking, alcohol, diabetes), and sample handling parameters (ischemic time). We also included Immune and Stromal scores computed by ESTIMATE (Yoshihara et al., 2013) as predictors because they reflect crucial TME properties (Figure 6E). In our linear regression analysis, stage, grade, age, BMI, ischemic time, and Immune and Stromal score were used as numerical variables, whereas the remaining variables were treated as categorical. For alcohol and smoking status, lifetime non-drinkers or non-smokers were compared to the rest of the population. For country of origin, European countries (in this case, Poland and Ukraine) were compared to all other countries. p values obtained for each gene in multivariate linear regression were corrected using Benjamini-Hochberg adjustment (Benjamini and Hochberg, 1995). The values of all adjusted p values for all clinical features and all data types in both tumor and adjacent normal are provided in Table S5. Figure S7A contains the number of genes with adjusted p value below 10%. To identify molecular pathways associated with tumor grade, we began with the pathway gene sets obtained from KEGG, Reactome, and Hallmark databases (Joshi-Tope et al., 2005; Kanehisa and Goto, 2000; Liberzon et al., 2011) and removed pathways that contained more than 500 genes. For proteome and mRNA data, we computed log10(FDR) for genes with positive associations and negative associations, where FDR is the adjusted p value for a gene’s association with grade. On this dataset, we ran a one-sided Wilcoxon rank-sum test for each pathway gene set versus all other genes. These p values were then corrected using Benjamini-Hochberg adjustment. A score for each pathway was computed as (+/−)log10(p-adj), depending on whether the rank-sum test p value showed it to be more up- or downregulated compared to other genes. Table S5 lists the scores of all pathways with adjusted p value <0.01. Figures 7A and S7B present a subset of pathways significantly associated with grade that were selected to be representative, non-redundant, and as informative as possible regarding the biological functions contained in the full set.

#### Immune-based Clustering of ccRCC tumors

##### Subtype identification based on cell type composition

The abundances of 64 different cell types in 175 ccRCC samples (103 tumor samples and 72 NAT samples) were computed via xCell (Aran et al., 2017). For this analysis, FPKM (Fragments Per Kilobase Million) mRNA expression values were utilized. Table S7 contains the final score computed by xCell for different cell types for the 175 samples. Based on these 64 signatures, consensus clustering was performed to identify groups of samples with the same immune/stromal characteristics. Consensus clustering was performed using the R packages ConsensusClusterPlus (Monti et al., 2003; Wilkerson and Hayes, 2010) within the Bioconductor package CancerSubtypes (Xu et al., 2017). Specifically, 80% of the original 175 samples were randomly subsampled without replacement and partitioned into six major clusters using the Partitioning Around Medoids (PAM) algorithm, which was repeated 200 times (Wilkerson and Hayes, 2010). Figure 6A shows the heatmap of scores for key cell types from the 175 ccRCC samples. The four tumor sample-based subtypes were tested for association with clinical variables (e.g., tumor grade) and genomic aberrations such as chr14 loss via a Chi-Square test of independence (Table S7). The upregulation of immune and stromal cells in a particular immune group based on tumor samples was assessed using a multivariate linear regression in which the score of each immune and stromal cell was modeled as a function of immune groups. First, every score was normalized across tumor samples by subtracting the mean and dividing by the standard deviation. Then, the score of the *j*-th cell for sample *i* was modeled as:

(1) $$X_{i,j}=\sum_{k=1}^{4}\beta_{k,j}1\left(i\in I_{K}\right)+\varepsilon_{i,j}$$

with $\varepsilon_{i,j}∼N\left(0,\sigma_{j}\right),I_{k}$ being the set of samples belonging to the *k*-th immune cluster, 1 (*A*) being an indicator function equal to 1 if the event *A* occurs and 0 otherwise, and $\beta_{k,j}$ being the coefficient capturing the association between gene *j* and the *k*-th immune group. Benjamini-adjusted p values can be found in Table S7.

##### Estimation of Stromal and Immune Scores

ESTIMATE (Yoshihara et al., 2013) was also used to infer tumor purity and immune and stromal scores based on RNA-seq data and global proteomic data (Figure S6B; Table S7). For the analysis of global proteomic data, only proteins with no missing values across all samples were considered. As shown in Table S7, immune and stromal scores based on global proteomic data and RNA-seq data were highly correlated (i.e., a Pearson correlation between immune scores based on RNA-seq and proteomic data higher than 0.85 and Pearson correlation of stromal scores higher than 0.75 for both NAT samples and ccRCC tumor samples). For this comparison, only samples overlapping between the two data types were considered (i.e., 103 ccRCC tumor samples and 72 NAT samples).

##### Validation of microenvironment scores using DNA methylation data

Edec was used to infer the tumor composition from DNA methylation data (Table S7). Edec is based on the principle that DNA methylation measured from whole bulk tumor is the linear combination of measurements from individual cell types weighted by their cell proportions. For the reference methylation profiles, we collected DNA methylation data (represented as beta-values) for five cell types – kidney cancer epithelial cells, kidney normal epithelial cells, fibroblasts, endothelial cells and immune cells (Table S7). Using a one-versus-all t test, we selected the methylation probes that distinguish the given cell type from other cell types. The probes were then mixed for the data deconvolution.

##### Immunohistochemistry (IHC) validation of immune cell compositions

Formalin-fixed, paraffin-embedded 5 μm tissue sections were stained in batches for CD4, CD8, and CD163 in a central laboratory at the Johns Hopkins Hospital according to standard automated protocols. Deparaffinization and rehydration were performed, followed by antigen retrieval and antibody staining. CD4 and CD8IHC was performed using the Ventana Benchmark Ultra autostaining system (Roche) using mouse monoclonal anti-CD8 (C8144B) antibody (Cell marque) and rabbit monoclonal anti-CD4(Sp35) antibody (Roche), followed by detection with the iVIEW DAB Detection Kit (Roche). CD163 IHC was performed on the Leica Bond MAX autostaining system (Leica Biosystems) using anti-CD163 (10D6) antibody (Leica Biosystems) followed by detection with Bond Polymer Refine Detection kit (Leica Biosystems). For tissue section imaging, slides were imaged using a Ventana iScan HT slide scanner (Roche) and processed using the Ventana Virtuoso software (Roche) (Figure S6D).

##### Analysis of Differentially-Expressed Genes and Pathways

Genes that were upregulated and downregulated in each of the four immune clusters were identified based on 103 tumor samples. For each data type, every feature vector was normalized by subtracting the mean and dividing by the standard deviation. For each data type, the expression level of gene *j* and ccRCC sample *i* (i.e., $X_{i,j}$) was modeled via Equation (1). Model [1] was implemented for each gene *j.* Table S7 shows upregulated and downregulated genes identified based on different data types. Considering genes that were up- and downregulated with Benjamini’s adjusted p value lower than 10%, a Fisher’s exact test was implemented to derive enriched pathways (Figures 6B and S6E; Table S7) (Benjamini and Hochberg, 1995). For this analysis, pathways from the Reactome, KEGG and Hallmark databases were considered and as background the full list of gene/proteins observed under each data type was utilized. Pathway scores for 103 ccRCC tumor samples and 80 NAT samples were computed based on combined z-score using the R package GSVA (Hänzelmann et al., 2013). Pathway scores based on different data types can be found in Table S7. Only combined z-scores of some key enriched pathways (Figure S6E) were included.

##### Angiogenesis and T-Effector Signatures

Using package GSVA (Hänzelmann et al., 2013), Angiogenesis (VEGFA, KDR, ESM1, PECAM1, ANGPTL4, and CD34) and T-Effector (CD8A, EOMES, PRF1, IFNG, and CD274) signatures (McDermott et al., 2018) were computed for 103 ccRCC samples. Upregulation of these signatures in a particular immune group was assessed via (1) (Figure 6F; Table S7).

##### Immune-based clustering on The Cancer Genome Atlas (TCGA) data

Based on 103 ccRCC samples, we selected genes that were differentially expressed in each of the four immune groups (CD8+ Inflamed, CD8− Inflamed, VEGF Immune Desert, Metabolic Immune Desert) using the function TCGAanalyze_DEA from the package TCGAbiolinks (Colaprico et al., 2016) and following our previously-described workflow (Silva et al., 2016). In particular, only genes with 10% FDR cut-off and log fold change greater than 1 were selected. Following this procedure, 2,252 unique genes were selected across different immune groups (i.e., 1,067 for CD8+ Inflamed, 721 for CD8− Inflamed, 1,054 for VEGF Immune Desert and 898 for Metabolic Immune Desert). Based on this set of genes, the one-class regression model, OCRL (Sokolov et al., 2016) was applied to construct a CPTAC data-based classifier for each immune group. The logistic regression model was trained using the R CRAN package, gelnet. The OCRL pipeline returned a 2,252 dimensional vector of weights for each immune group, i.e., *W*_*i*_ with ∈ {1,2,3,4} Then, TCGA kidney renal clear cell carcinoma (KIRC) samples were allocated into immune groups based on two scores that were computed using the set of 2,252 pre-selected genes. Specifically, for the k-th sample in TCGA data and each immune group i, the following two scores were computed:

Score 1: Spearman correlation between the model’s weight vector and the *k*-th TCGA sample’s expression profile, i.e., *Z_ik_* = *cor* (*W_i_, X_k_*) with *X_k_* being a 2,252 dimensional vector containing expression levels of the 2,252 genes for the *k*-th TCGA sample. The correlation between *W_i_* and *X_k_* would be high if the *k*-th TCGA sample belonged to the *i*-th immune group. Scores {*Z_sk_*} were normalized to be in the unit interval {0,1} by subtracting the lowest value and then dividing by the maximum value.
Score 2: Spearman correlation between the *k*-th TCGA sample’s expression profile and the *s*-th CPTAC sample’s expression profile of 2,252 genes, i.e., *S_sk_* = *cor*(*Y_s_, X_k_*) with *Y_s_* being a 2,252 dimensional vector containing expression levels of the pre-selected 2,252 genes for the *k*-th CPTAC sample, *X_k_* being a 2,252 dimensional vector containing expression levels of the 2,252 genes for the *k*-th TCGA sample. This score was computed for each CPTAC sample s belonging to the *i*-th immune group. Scores ${\left\{S_{SK}\right\}}_{S\in l_{i}}$ were normalized to be in the unit interval {0,1}. The final score measuring the association between the *k*-th TCGA sample and the *i*-immune group (i.e., $Q_{iK}$) was obtained by averaging scores ${\left\{S_{SK}\right\}}_{S\in l_{i}}$ with $l_{i}$ being the set of samples in the *j*-th immune group.

The first score (i.e., $Z_{iK}$ ) was utilized previously to classify samples (Malta et al., 2018). In this study, a second score was considered to avoid cases in which multiple immune categories resulted in the same score. In particular, the final score was derived by averaging scores $\left\{Z_{iK}\right\}\;\mathrm{and}\;\left\{Q_{iK}\right\}$, i.e., $X_{iK}=\left(Z_{iK}+Q_{iK}\right)/2$. Finally, to each TCGA sample *k*, the immune group with the highest score was assigned, i.e., $G_{k}=\arg\max_{i}{}_{K}$. This final score can be found in Table S7. This classification resulted in 126 samples allocated to the CD8− Inflamed group, 156 samples allocated totheCD8+ Inflamed group, 135 samples allocated to the Metabolic Immune Desert and 78 samples allocated to the VEGF Immune Desert group (Table S7). This TCGA-based classification was compared to that based on CPTAC data in terms of immune and stromal cell infiltration, pathway activities and key markers that were found to be upregulated in different immune groups based on CPTAC data. Similarly to CPTAC data, the concentration of different immune and stromal cells was computed via xCell (Aran et al., 2017) (Table S7), while the activity of key pathways was derived via a combined z-score (Hänzelmann et al., 2013) (Figure S6E; Table S7). To identify immune and stromal cells upregulated in different immune groups, the strategy adopted for CPTAC data was utilized (Model 1, Table S7).

##### Clinical Outcome of Immune Groups

Immune groups based on TCGA data were utilized to better understand the clinical outcome and expected survival for different immune groups (Figures S6G and S6H). Overall survival data and tumor grade information for 495 TCGA KIRC samples, deposited in the Genomic Data Commons (GDC) Data Portal, were downloaded using the function GDCquery_clinic from the package TCGAbiolinks (Colaprico et al., 2016). Table S7 shows Benjamini’s adjusted p values (Benjamini and Hochberg, 1995) from a pairwise Log Rank test comparing survival curves that correspond to different immune groups. Kaplan-Meier overall survival curves were generated using the function TCGAanalyze_survival from the package TCGAbiolinks (Colaprico et al., 2016). The association between high-grade tumors (i.e., grade 3 and 4) and immune groups was assessed via a Chi-Square test of independence (Table S7).

### QUANTIFICATION AND STATISTICAL ANALYSIS

#### Transcriptomic Quantitation

The resulting BAM files were analyzed for expression using feature counts against a transcriptomic reference based on Gencode 26. The resulting gene-level counts for protein-coding genes were transformed into FPKMs using edgeR.

#### Proteomic and Phosphoproteomic Quantitation

Whole proteome and phosphopeptide-enriched datasets were processed separately but using the same pipeline steps as described in the “Protein database searching and quantification of global and phosphoproteomic data” section of the STAR Methods. A tutorial describing all steps of the analysis, including specific input parameter files, command-line option, and all software tools necessary to replicate the results are available at https://github.com/Nesvilab.

The statistical details of all experiments have been reported in the manuscript text, figure legends and corresponding STAR Methods section descriptions. Data analysis was performed in Excel, R, and Python.

### DATA AND CODE AVAILABILITY

Raw data files for proteomic analysis reported in this paper are hosted by the CPTAC Data Portal and can be accessed at: https://cptac-data-portal.georgetown.edu/cptac/s/S044 and https://cptac-data-portal.georgetown.edu/cptac/s/S050. Genomic and transcriptomic data files can be accessed at: https://portal.gdc.cancer.gov/. Processed data utilized for this publication can be accessed at: https://cptac-data-portal.georgetown.edu/cptac/s/S050.

Several custom coding softwares were generated as part of this study and have been referenced in the corresponding STAR Methods section and listed with links to the coding script in the Key Resources Table: software codes generated by the Cieslik laboratory for genomic analyses (CNVEX and CRISP), by the Nesvizhskii laboratory for proteomic data processing (Philosopher and TMT-Integrator) by the Wang lab for data imputation (DreamAI), and by the Zhang lab for data processing and neoantigen detection (NeoFlow and PepQuery).

Interactive data analysis tools were generated by the Wang lab: a web-based application for visualizing a heatmap of 22,867 genes across 110 samples can be accessed at: http://ccrcc.cptac-data-view.org; a web-based application for interactively exploring ccRCC phosphoproteomic co-expression networks (3,614 nodes, 11,200 edges) and their modules is available at: http://ccrcc.cptac-network-view.org/.

#### Additional Resources

The CPTAC program website, which includes details about program initiatives, investigators, and datasets, can be accessed at: https://proteomics.cancer.gov/programs/cptac

## Supplementary Material

Table S1

Table S2

Table S4

Table S5

Table S3

Table S6

Table S7

8

## Figures and Tables

**Table:** KEY RESOURCES TABLE

REAGENT or RESOURCE	SOURCE	IDENTIFIER
Antibodies		
Mouse monoclonal anti-CD8 (C8/144B)	Cellmarque	Catalog #108M-96; RRID: AB_1158208
Rabbit monoclonal anti-CD4 (SP35)	Roche	Catalog #790-4423
Liquid Concentrated Monoclonal Antibody anti-CD163	Leica Biosystems	Catalog #NCL-L-163; RRID: AB_2756375
Biological Samples		
Primary tumor samples	See Experimental Model and Subject Details	N/A
Chemicals, Peptides, and Recombinant Proteins		
Aprotinin	Sigma	Catalog: A6103
Leupeptin	Roche	Catalog: 11017101001
Phenylmethylsulfonyl fluoride	Sigma	Catalog:93482
Sodium fluoride	Sigma	Catalog: S7920
Phosphatase Inhibitor Cocktail 2	Sigma	Catalog: P5726
Phosphatase Inhibitor Cocktail 3	Sigma	Catalog: P0044
Urea	Sigma	Catalog: U0631
Tris(hydroxymethyl)aminomethane	Invitrogen	Catalog: AM9855G
Ethylenediaminetetraacetic acid	Sigma	Catalog: E7889
Sodium chloride	Santa Cruz Biotechnology	Catalog: sc-295833
PUGNAc	Sigma	Catalog: A7229
Dithiothretiol	ThermoFisher Scientific	Catalog: 20291
Iodoacetamide	ThermoFisher Scientific	Catalog: A3221
Sequencing grade modified trypsin	Promega	Catalog: V511X
Lysyl endopeptidase, aass spectrometry grade	Wako Chemicals	Catalog: 125-05061
Formic acid	Fisher Chemical	Catalog: A117-50
Reversed-phase C18 SepPak	Waters	Catalog: WAT054925
4-(2-hydroxyethyl)-1-piperazineethanesulfonic acid	Alfa Aesar	Catalog: J63218
Tandem mass tags – 10plex	ThermoFisher Scientific	Catalog: 90110
Trifluoroacetic acid	Sigma	Catalog: 302031
Ammonium Hydroxide solution	Sigma	Catalog: 338818
Hydroxylamine solution	Aldrich	Catalog: 467804
Ni-NTA agarose beads	QIAGEN	Catalog: 30410
Iron (III) chloride	Sigma	Catalog:451649
iVIEW DAB Detection Kit	Roche	Catalog: 760-091
Bond Polymer Refine Detection Kit	Leica Biosystems	Catalog: DS9800
Critical Commercial Assays		
TruSeq Stranded Total RNA Library Prep Kit with Ribo-Zero Gold	Illumina	Catalog: RS-122-2301
Infinium MethylationEPIC Kit	Illumina	Catalog: WG-317-1003
Nextera DNA Exosome Kit	Illumina	Catalog: 20020617
KAPA Hyper Prep Kit, PCR-free	Roche	Catalog: 07962371001
BCA Protein Assay Kit	ThermoFisher Scientific	Catalog: 23225
Deposited Data		
PhosphoSitePlus	Hornbeck et al., 2015	https://www.phosphosite.org
GTEx	Ardlie et al., 2015	https://gtexportal.org/home/
TCGA - ccRCC	Creighton et al., 2013	https://portal.gdc.cancer.gov/
Software and Algorithms		
methylationArrayAnalysis (version 3.9)	Maksimovic et al., 2016	https://master.bioconductor.org/packages/release/workflows/html/methylationArrayAnalysis.html
Illumina EPIC methylation array (3.9)	Fortin et al., 2017	https://bioconductor.org/packages/release/data/annotation/html/IlluminaHumanMethylationEPICanno.ilm10b2.hg19.html
VarDict	Lai et al., 2016	https://github.com/AstraZeneca-NGS/VarDict
Strelka2	Kim et al., 2018	https://github.com/Illumina/strelka
MUTECT2	Cibulskis et al., 2013	https://software.broadinstitute.org/gatk/documentation/tooldocs/3.8-0/org_broadinstitute_gatk_tools_walkers_cancer_m2_MuTect2.php
VarScan2.3.8	Koboldt et al., 2012	http://varscan.sourceforge.net
Pindel0.2.5	Ye et al., 2009	http://gmt.genome.wustl.edu/packages/pindel/
CNVEX	Marcin Cieslik Lab	https://github.com/mctp/cnvex
CRISP	Marcin Cieslik Lab	https://github.com/mcieslik-mctp/crisp-build
Proteowizard	Kessner et al., 2008	http://proteowizard.sourceforge.net/
MSFragger	Kong et al., 2017	https://msfragger.nesvilab.org/
Philosopher	Alexey Nesvizhskii Lab	https://philosopher.nesvilab.org/
PeptideProphet	Keller et al., 2002	http://peptideprophet.sourceforge.net/
ProteinProphet	Nesvizhskii et al., 2003	http://proteinprophet.sourceforge.net/prot-proph.pdf
PTMProphet	Deutsch et al., 2015	http://www.tppms.org/tools/ptm/
TMT-Integrator	Alexey Nesvizhskii Lab	https://github.com/Nesvilab/TMT-Integrator
DIA-Umpire	Tsou et al., 2015	https://github.com/Nesvilab/DIA-Umpire
msproteomicstools	http://msproteomicstools.roestlab.org/	https://github.com/msproteomicstools
ComBat (v3.20.0)	Johnson et al., 2007	https://bioconductor.org/packages/release/bioc/html/sva.html
DreamAI	Pei Wang Lab	https://github.com/WangLab-MSSM/DreamAI
GISTIC2.0	Mermel et al., 2011	ftp://ftp.broadinstitute.org/pub/GISTIC2.0/GISTIC_2_0_23.tar.gz
iProFun	Song et al., 2019	https://github.com/WangLab-MSSM/iProFun
ESTIMATE	Yoshihara et al., 2013	https://bioinformatics.mdanderson.org/public-software/estimate/
WebGestaltR	Wang et al., 2017	http://www.webgestalt.org/
Joint Random Forest	Petralia et al., 2016	https://github.com/WangLab-MSSM/ptmJRF
GSVA	Hänzelmann et al., 2013	https://bioconductor.org/packages/release/bioc/html/GSVA.html
TCGAbiolinks	Colaprico et al., 2016	http://bioconductor.org/packages/release/bioc/html/TCGAbiolinks.html
Cytoscape	Shannon et al., 2003	https://cytoscape.org/
TSNet	Petralia et al., 2018	https://github.com/WangLab-MSSM/TSNet
xCell	Aran et al., 2017	http://xcell.ucsf.edu/
CPTAC Network Exploration Portal	Pei Wang Lab	http://ccrcc.cptac-network-view.org/
CPTAC Data Viewer	Pei Wang Lab	http://ccrcc.cptac-data-view.org/
iCAVE	Liluashvili et al., 2017	http://labs.icahn.mssm.edu/gumuslab/software
MODMatcher	Yoo et al., 2014	https://github.com/integrativenetworkbiology/Modmatcher
ConsensusClusterPlus	Monti et al., 2003; Wilkerson and Hayes, 2010	http://bioconductor.org/packages/release/bioc/html/CancerSubtypes.html
OmicsX	Pan et al., 2019	http://bioinfo.wilmer.jhu.edu/OmicsX/
Omic-Sig	Lih et al., 2019	https://github.com/hzhangjhu/Omic-Sig
OmicsOne	Hu et al., 2019	https://github.com/HuiZhangLab-JHU/OmicsOne
pyQUILTS (v1.0)	Ruggles et al., 2016	http://openslice.fenyolab.org/cgi-bin/pyquilts_cgi.pl
MS-GF+	Kim and Pevzner, 2014	https://github.com/MSGFPlus/msgfplus
NeoFlow	Bing Zhang Lab	https://github.com/bzhanglab/neoflow
netMHCpan	Jurtz et al., 2017	http://www.cbs.dtu.dk/services/NetMHCpan/
Optitype	Szolek et al., 2014	https://github.com/FRED-2/OptiType
Customprodbj	Bing Zhang Lab	https://github.com/bzhanglab/customprodbj
PDV	Li et al., 2019	https://github.com/wenbostar/PDV
PeoQuery	Wen et al., 2019	http://pepquery.org

## Highlights

- Integrated proteogenomic characterization in 103 ccRCC cases
- Delineation of chromosomal translocation events leading to chromosome 3p loss
- Tumor-specific proteomic/phosphoproteomic alterations unrevealed by mRNA analysis
- Immune-based subtypes of ccRCC defined by mRNA, proteome, and phosphoproteome
