# Supplementary material for: Integrated Proteogenomic Characterization of Clear Cell Renal Cell Carcinoma
Source: Cell. Author manuscript; Available in PMC 2020 Aug 11. (PMC7331093; doi:10.1016/j.cell.2019.10.007)
Supplement: 8 [file NIHMS1597713-supplement-8.pdf]

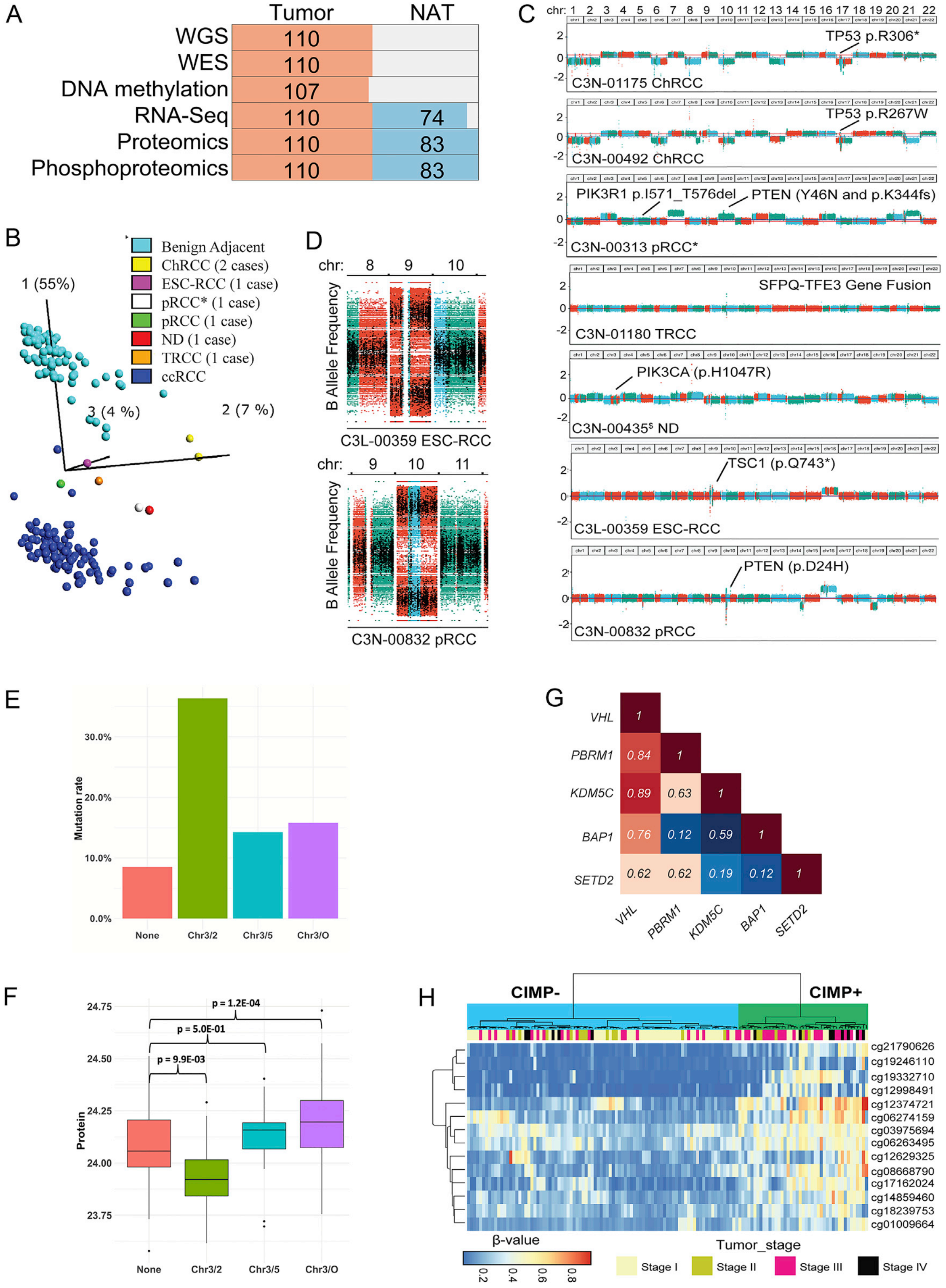

Figure S1. Impact of Genomic Alterations on Transcriptomic and Proteomic Abundances, Related to Figure 1

(A) The number of RCC and NAT samples subjected to “WGS” (whole genome sequencing), “WES” (whole exome sequencing), “DNA Methylation,” “RNA-seq,” “Proteomics,” and “Phosphoproteomics” analyses. The presented numbers reflect the number of samples processed that passed data quality control (QC) metrics. (B) Principal Component Analysis (PCA) of RNA-seq data for all CPTAC tumors (n = 110). Identified outliers are annotated by color coding (right).

(legend continued on next page)

(C) Outlier samples were further investigated for copy number variations (WGS) and somatic mutations (WES). (D) B-allele frequency plots (derived from WES (black dots) and WGS (colored)) for two samples indicate loss of heterozygosity by uniparental disomy for chr 9 and 10 for two index samples. (E) Frequency of *SETD2* mutation distribution across tumors without translocation events and tumors with t(3:2) (ch3/2), t(3:5) (ch3/5), and other (ch3/O) translocation events. (F) SETD2 protein abundance distribution across tumors without translocation events and tumors with t(3:2) (ch3/2), t(3:5) (ch3/5), and other (ch3/O) translocation events. (G) Co-occurrence of *VHL*, *PBRM1*, *BAP1*, *SETD2*, and *KDM5C* gene mutations in ccRCC tumors (n = 103). (H) Heatmap of 14 CpG probe beta-values associated with the CpG island methylator phenotype (CIMP) profile of ccRCC tumors. CIMP status and Stage are annotated (top).

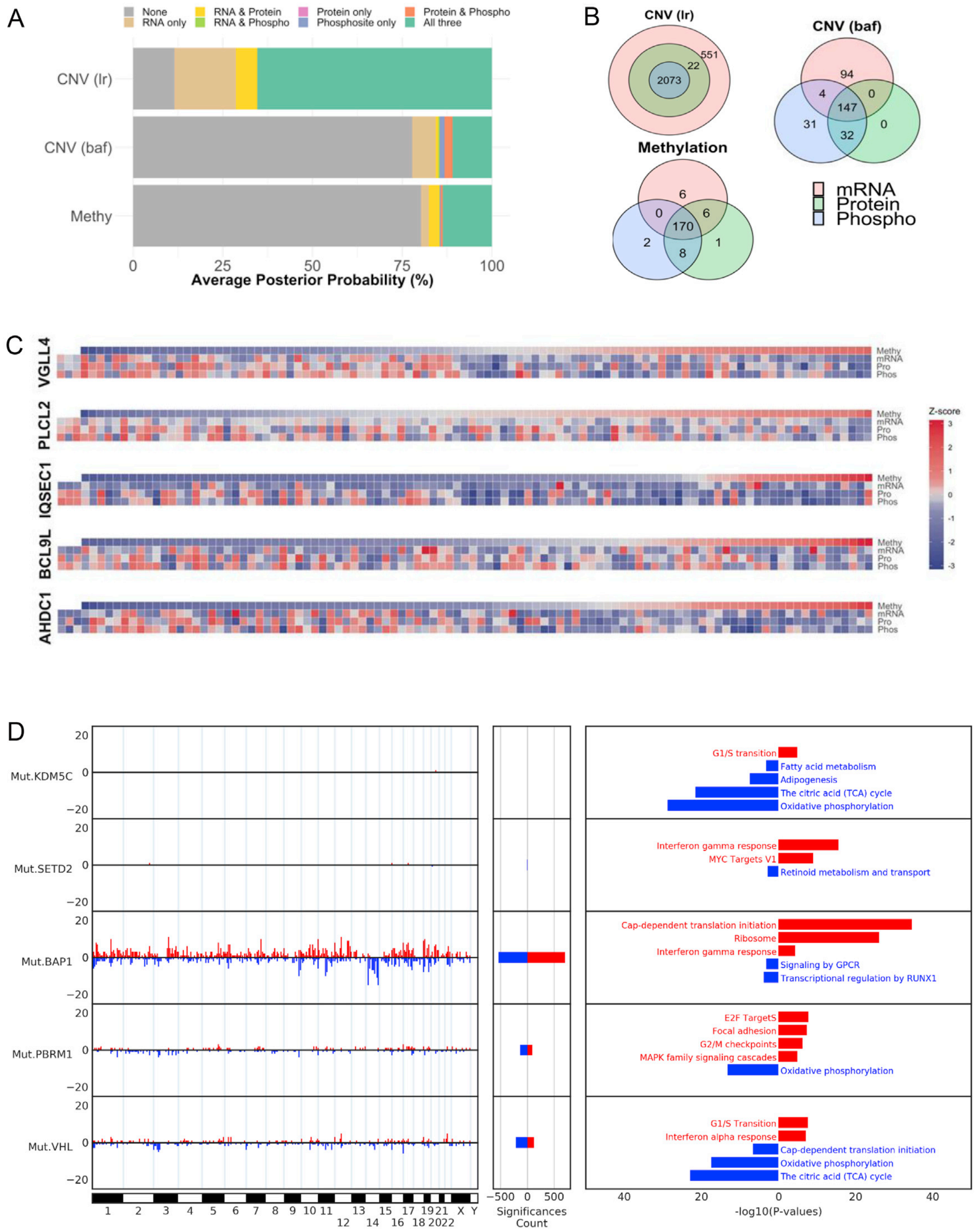

Figure S2. Impact of Copy Number Variation (CNV) on Protein Abundance, Related to Figure 2

(A) Results of iProFun associations of CNV lr (adjusted log coverage-ratios), CNV baf (B-allele frequencies), and DNA methylation. Average posterior probability was calculated for association of genomic alteration events and mRNA, global protein, and phosphosite abundance. (B) Venn diagram of CNV (lr), CNV (baf), and DNA methylation genomic alterations for mRNA, protein, and phosphosite association overlap. (C) Heatmap of multi-omic data for methylation *cis*-cascade event (*VGLL4*, *PLCL2*) and for DNA methylation not detected at the mRNA level (protein and phosphosite only) (*IQSEC1*, *BCL9L*, *AHDC1*). Tumor samples were ordered by increasing DNA methylation z-score (left to right). (D) Associated enriched upregulated (red) and downregulated (blue) pathways of *cis*- and *trans*-effects for five key tumor suppressor genes (*VHL*, *PBRM1*, *BAP1*, *SETD2*, and *KDM5C*) (adjusted $p < 0.05$).

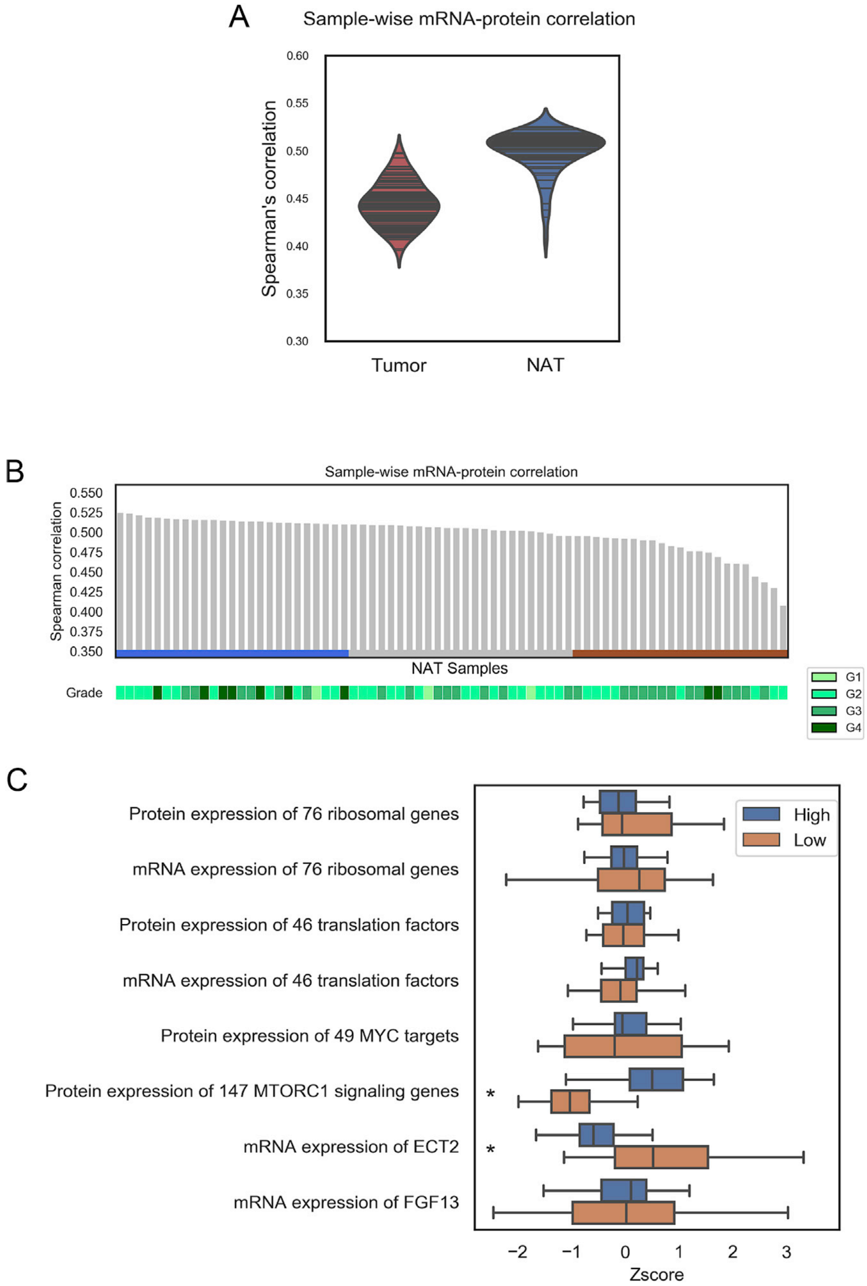

Figure S3. Correlation of mRNA and Protein Abundance in ccRCC and NATs, Related to Figure 3

(A) Violin plots of tumor and NAT sample-wise correlation frequency and median distribution. (B) Sample-wise correlation of NATs ranked from high to low. NATs were evenly distributed into three groups: high (blue), middle (gray), and low (gold). Corresponding tumor grade is indicated below. (C) Boxplots of ribosomal gene expression (mRNA and protein), translation factor expression (mRNA and protein), and Pol I associated regulatory factors based on NAT high/low sample-wise correlation ranking (*p < 0.05).

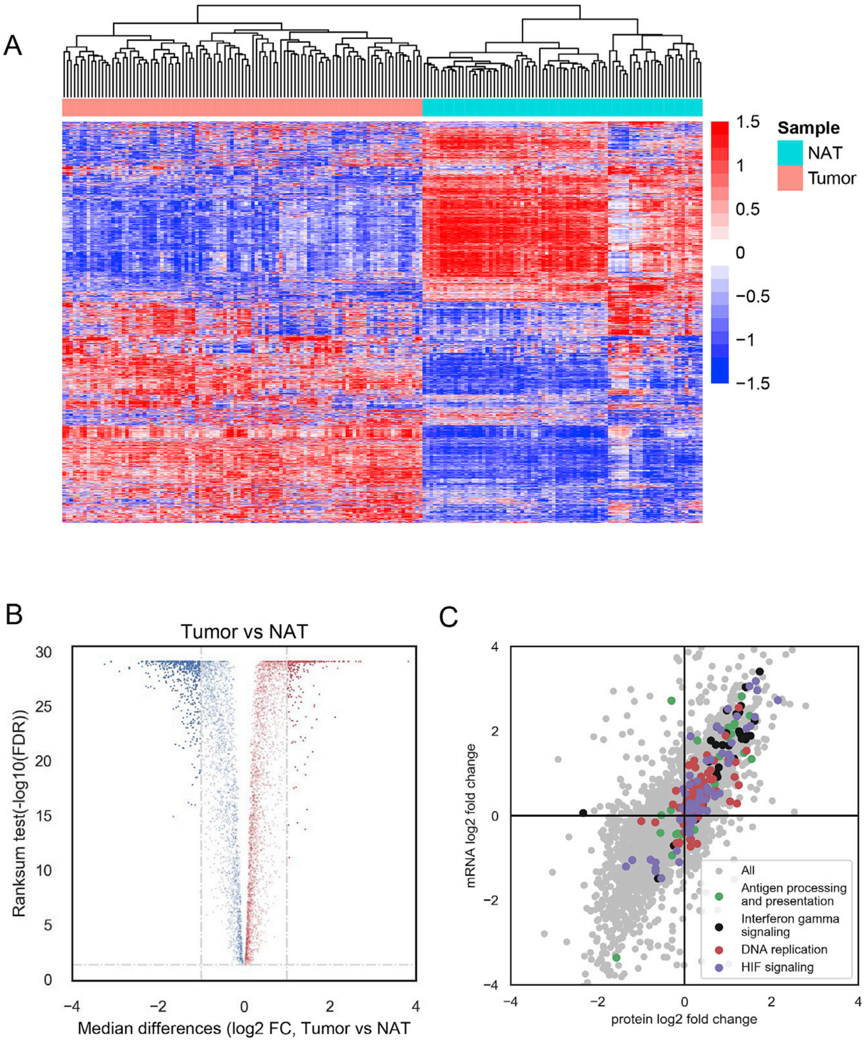

Figure S4. Differential Expression of Transcriptomic and Proteomic Profiles between ccRCC and NAT Protein Expression, Related to Figure 4

(A) Heatmap of protein expression abundances between ccRCC and NAT samples. Hierarchical clustering discriminated tumors (left) from NAT (right) (n = 5745) (B) Volcano plot of significantly differentially expressed proteins (log2 fold-change > 1; Benjamini-Hochberg adjusted $p < 0.05$) (n = 820; 565 downregulated, 255 upregulated). (C) Scatterplot of differential gene expression between ccRCC tumors and NATs at mRNA and protein levels using log2-fold changes. Each dot represents one gene; x axis: protein, y axis: mRNA, respectively. Negative values indicate a decrease and positive values an increase in gene expression between tumor and NAT. Upregulated pathways in ccRCC samples were shown.

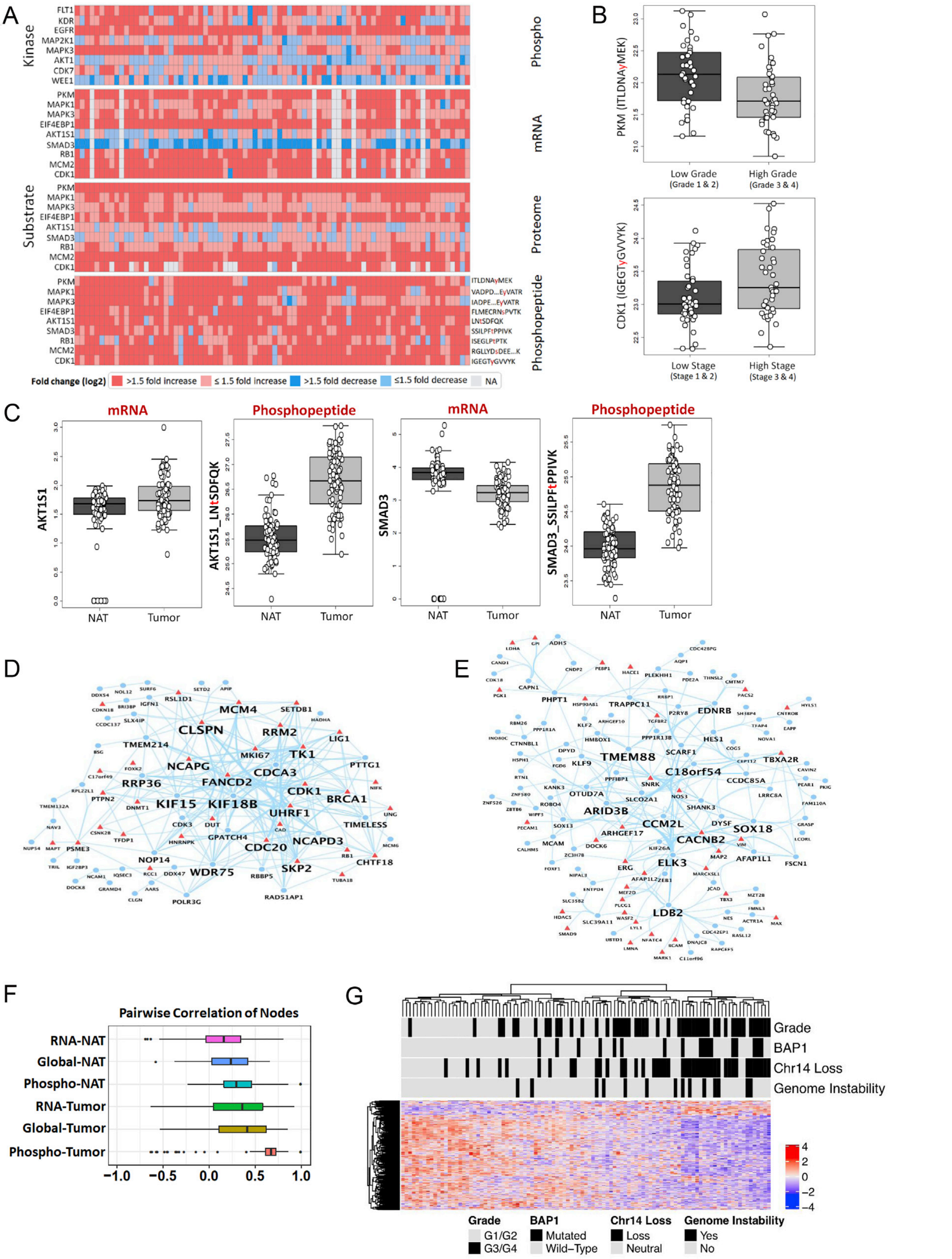

(legend on next page)

Figure S5. Expression Changes of Phospho-Substrates in Multiple Omics Data and the Network Module of Cell Cycle, Related to Figure 5

(A) Fold change (log2 scale) of 80 tumor/NAT pairs in phospho-substrates based on mRNA and global proteome expression. (B) Comparison of phosphorylation levels of PKM (top) and CDK1 (bottom) phosphopeptides between low grade/stage tumors and high grade/stage tumors (Mann–Whitney U test, $p < 0.05$). (C) Two phospho-substrates, AKT1S1 and SMAD3 showed substantial differences in mRNA and phosphopeptide expression when comparing tumors to NATs. (D) Phosphopeptide co-expression network module displaying “Cell Cycle” pathway enrichment. (E) Phosphopeptide co-expression network module displaying “Angiogenesis” pathway enrichment. (F) Pairwise correlation of nodes at multi-omics levels of the “Angiogenesis” co-expression network module. (G) Heatmap of “Angiogenesis” module expression with grade, BAP1 and chromosome 14 loss, and genome instability distribution annotated.

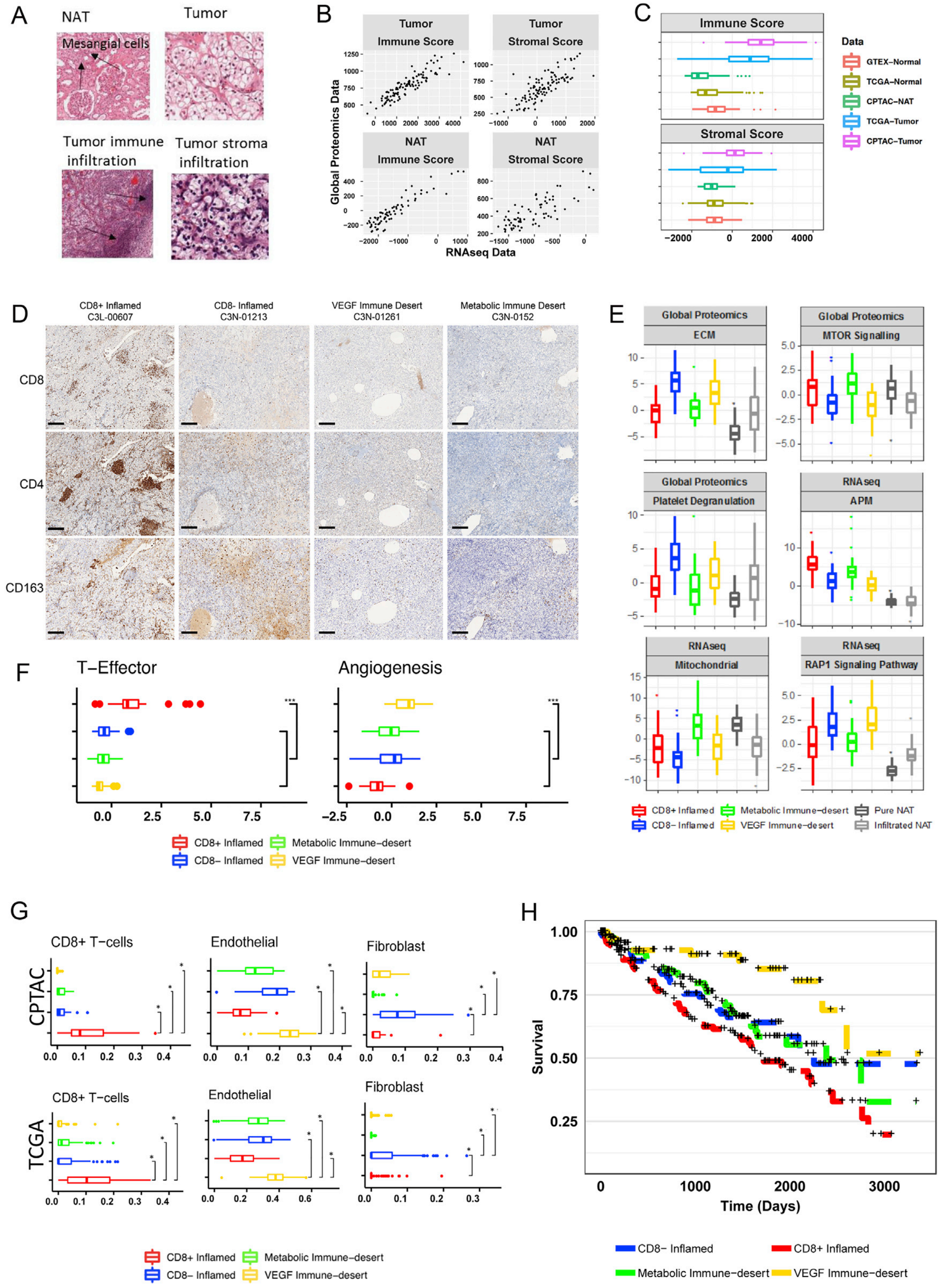

Figure S6. Immune-Based Subtyping of ccRCC Tumors, Related to Figure 6

(A) Histologic assessment of xCell-generated cell signatures. Representative H&E stained tissue sections verifying mesangial cell, immune infiltration, and stromal infiltration signatures. (B) Comparison between RNA-seq and global proteomics in estimating immune and stromal cell infiltration based on 103 ccRCC tumor samples and 72 NAT samples. Correlation between scores based on RNA-seq and global proteomics data was higher than 0.75 for both tissue types.

(legend continued on next page)

Stromal and immune scores were computed via ESTIMATE. (C) Comparison of stromal and immune scores between CPTAC, TCGA, and GTEx data. Stromal and immune scores were computed via ESTIMATE using RNA-seq data. (D) IHC profiling of immune cell populations in ccRCC. FFPE sections were stained for CD8, CD4, and CD163 protein markers to determine CD8+ T cell, CD4+ T cell, and macrophage abundance in ccRCC tumor tissues. The scale bar indicates 200 μm. (E) Boxplot of pathway scores for different immune groups based on RNA-seq and proteomics data from 103 CPTAC ccRCC tumor samples and 80 CPTAC NAT samples. Pathway scores were computed via combined z-score. Pathways upregulated in CD8+ Inflamed tumors included "APM," while pathways upregulated in CD8- Inflamed tumors included "ECM" and "Platelet Degranulation." Pathways upregulated in VEGF Immune Desert tumors included "RAP1 Signaling," while pathways upregulated in Metabolic Immune Desert tumors included "MTOR Signaling" and "Mitochondrial." (F) Z-Score based on Angiogenesis (*VEGFA*, *KDR*, *ESM1*, *PECAM1*, *ANGPTL4*, *CD34*) and T-Effector (*CD8A*, *EOMES*, *PRF1*, *IFNG*, *CD274*) signatures from McDermott et al. (2018). Upregulation of each signature in a particular immune group was assessed via multivariate model (*** adjusted $p < 0.001$). (G) Distribution of CD8+ T cells, endothelial, and fibroblast cell concentrations for different immune groups based on 103 CPTAC ccRCC samples and 495 TCGA KIRC samples; RNA-seq-derived cell signatures. (H) Kaplan-Meier curves of survival data stratified by immune groups derived based on 495 TCGA KIRC samples. VEGF Immune Desert samples displayed increased overall compared to other groups (p value = 0.0004). CD8+ Inflamed subgroup samples displayed decreased overall survival compared to all the other groups (p value = 5e-04).

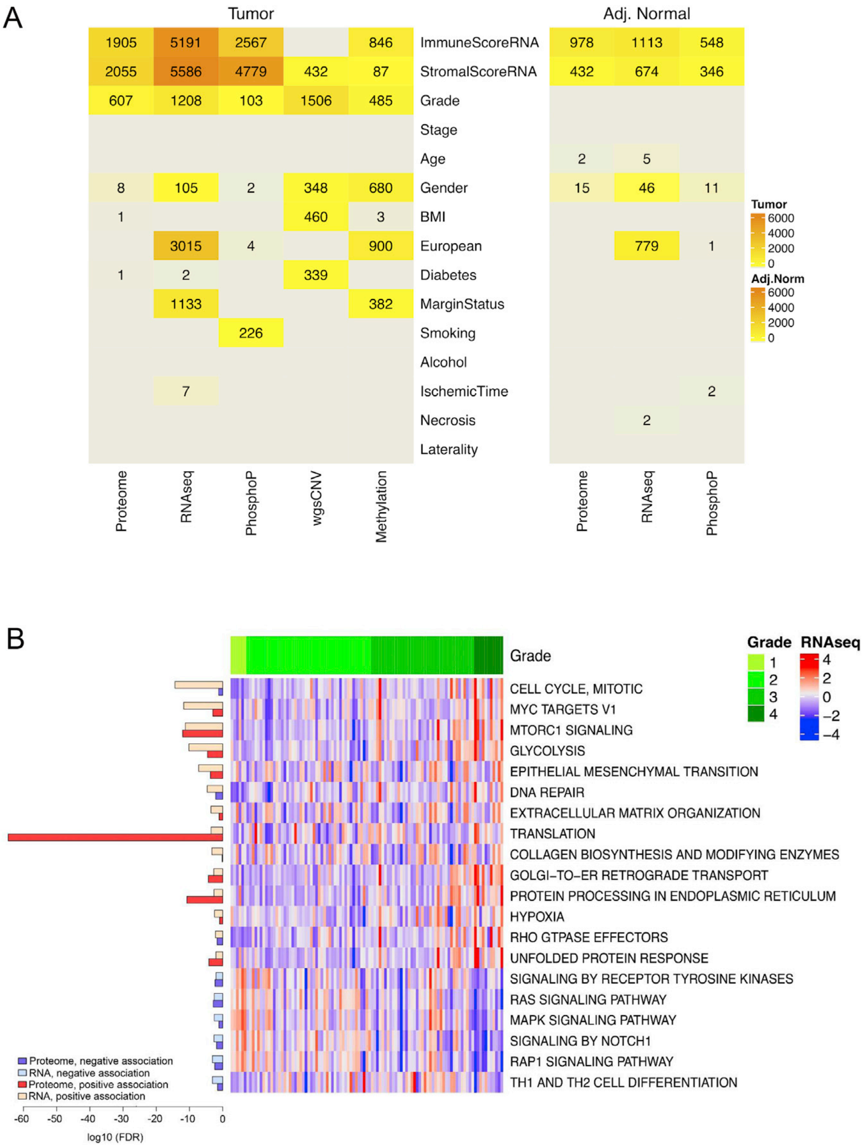

Figure S7. Proteomic Inter-Tumor Heterogeneity of ccRCC and Associated Functional Pathways, Related to Figure 7

(A) Results from multivariate analysis association of multiple "omics" levels. RNA-based Immune Score, RNA-based Stromal Score, and grade associated with multiple data types (B) Cellular pathways (right) with positive (red) or negative (blue) associations (adjusted $p < 0.05$) at protein or mRNA level (left). Heatmap of mRNA transcript expression associated with high and low grade tumors (center) (Benjamini-Hochberg adjusted $p < 0.05$).
